# Supplementary material for: RNA Docking and Local Translation Regulate Site-Specific Axon Remodeling In Vivo
Source: Neuron. 2017 Aug 16;95(4):852–868.e8. doi: 10.1016/j.neuron.2017.07.016 (PMC5563073; doi:10.1016/j.neuron.2017.07.016)
Supplement: Document S1. Figures S1–S7 [file mmc1.pdf]

Neuron, Volume *95*

Supplemental Information

RNA Docking and Local Translation

Regulate Site-Specific Axon Remodeling In Vivo

Hovy Ho-Wai Wong, Julie Qiaojin Lin, Florian Ströhl, Cláudio Gouveia Roque, Jean-Michel Cioni, Roberta Cagnetta, Benita Turner-Bridger, Romain F. Laine, William A. Harris, Clemens F. Kaminski, and Christine E. Holt

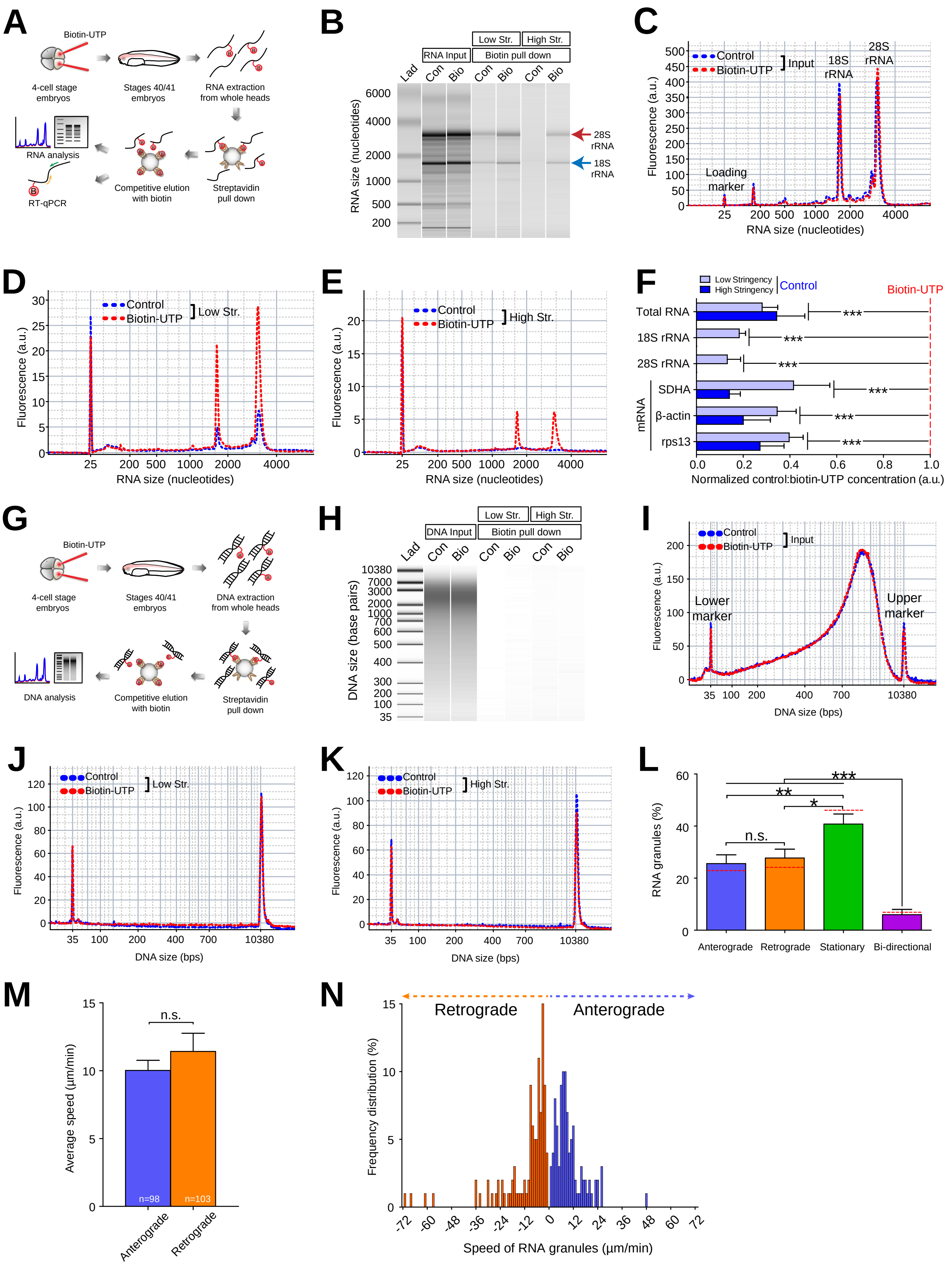
A
Biotin-UTP
4-cell stage embryos
Stages 40/41 embryos
RNA extraction from whole heads
RNA analysis
RT-qPCR
Competitive elution with biotin
Streptavidin pull down
B
RNA Input
Low Str.
High Str.
Biotin pull down
Lad
Con
Bio
RNA size (nucleotides)
28S rRNA
18S rRNA
C
Control
Biotin-UTP
Input
Loading marker
18S rRNA
28S rRNA
Fluorescence (a.u.)
RNA size (nucleotides)
D
Low Str.
E
High Str.
F
Low Stringency
High Stringency
Control
Biotin-UTP
Total RNA
18S rRNA
28S rRNA
SDHA
β-actin
rps13
mRNA
Normalized control:biotin-UTP concentration (a.u.)
G
DNA extraction from whole heads
DNA analysis
H
DNA Input
DNA size (base pairs)
I
Lower marker
Upper marker
DNA size (bps)
J
K
L
n.s.
RNA granules (%)
Anterograde
Retrograde
Stationary
Bi-directional
M
Average speed (µm/min)
n=98
n=103
N
Retrograde
Anterograde
Frequency distribution (%)
Speed of RNA granules (µm/min)

Figure S1

Figure S1. Related to Figure 1. UTP Analogue Incorporates into mRNA and rRNA, but Not DNA, *in vivo*

(A) Introduction of biotin-UTP into the CNS prior to RNA extraction and streptavidin pull down for Bioanalyzer analysis and RT-qPCR. (B) RNA gel image from the Bioanalyzer analysis. Str., Stringency; Lad, ladder; Con, control; Bio, biotin-UTP. (C) Electropherogram showing that the global RNA patterns are similar for the input of control (blue) and biotin-UTP (red) conditions. (D and E) Electropherograms displaying the enrichment of RNA in the biotin-UTP condition with both the low (D) and high (E) stringency pull down protocols. (F) Bar charts presenting the enrichment of total RNA ($F_{1,8}$=99.67, p<0.0001), 18S rRNA ($F_{1,8}$=4601, p<0.0001), 28S rRNA ($F_{1,8}$=1087, p<0.0001), SDHA mRNA ($F_{1,7}$=158.9, p<0.0001), β-actin mRNA ($F_{1,8}$=105.4, p<0.0001) and rps13 mRNA ($F_{1,8}$=125.1, p<0.0001) in the biotin-UTP condition over control condition with both the low and high stringency pull down protocols. (G) Introduction of biotin-UTP into the CNS prior to genomic and mitochondrial DNA extraction and streptavidin pull down for Bioanalyzer analysis. (H) DNA gel image from the Bioanalyzer analysis. (I) Electropherogram showing the global DNA patterns are similar for the input of control (blue) and biotin-UTP (red) conditions. (J and K) Electropherograms showing no obvious DNA peak was detected in either control or biotin-UTP condition with both the low (J) and high (K) stringency pull down protocols. (L) The proportions of RNA motion in 20 individual axons are displayed as bars ($F_{3,76}$=19.7, p<0.0001). The proportions of RNA motion pooled together from all RNA granules (n=319) are displayed as red dotted lines. (M) Average speed of anterograde and retrograde trafficking of RNA granules (U=4463, p=0.16, Mann Whitney test). (N) Frequency distribution of the speed of RNA granules trafficked in the anterograde and retrograde directions. Error bars represent SEM. *p<0.05, **p<0.01, ***p<0.001 (two-way ANOVA for (F)) (one-way ANOVA with Tukey multiple comparisons test for (L)).

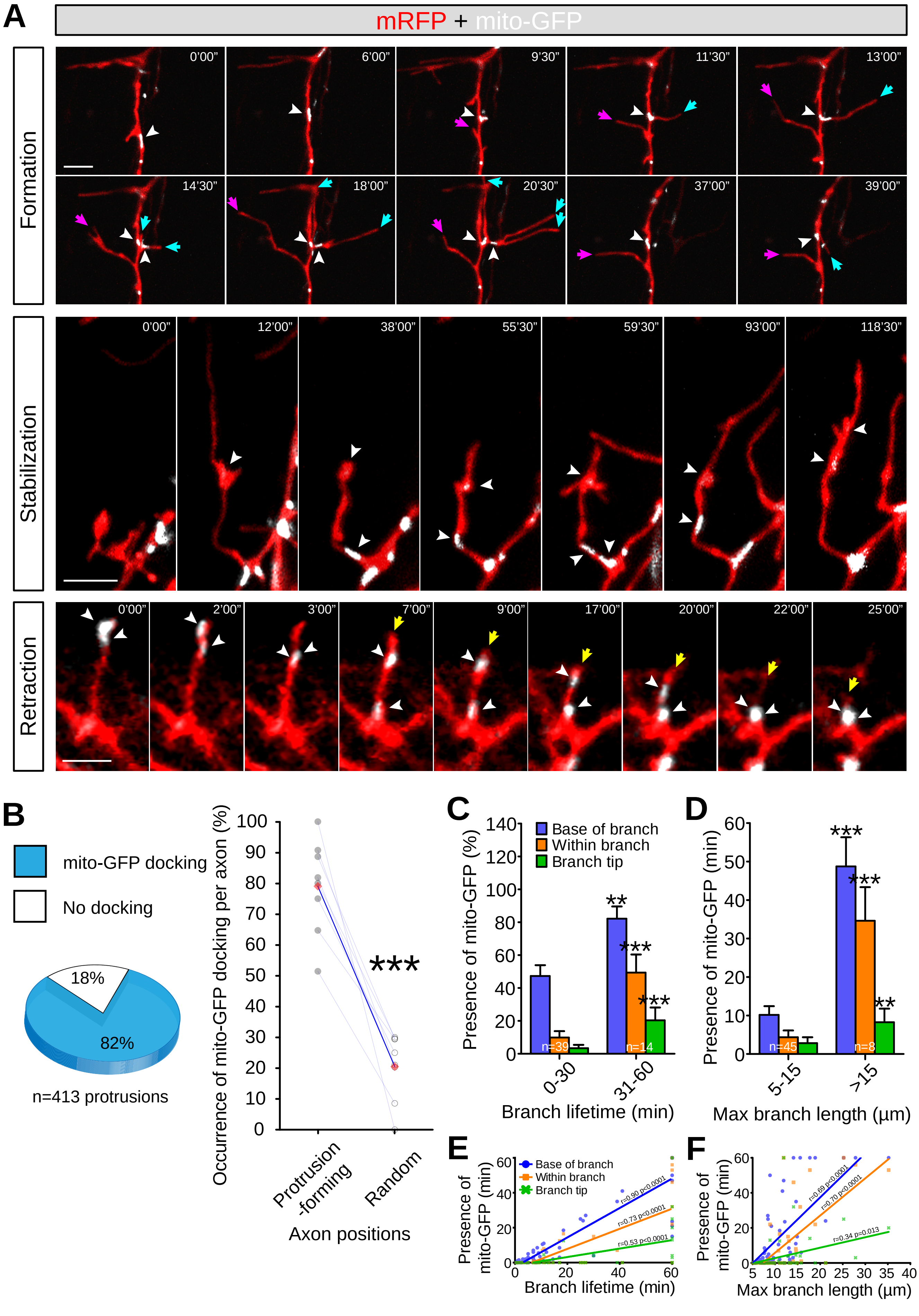

A
mRFP + mito-GFP
Formation
0'00"
6'00"
9'30"
11'30"
13'00"
14'30"
18'00"
20'30"
37'00"
39'00"
Stabilization
0'00"
12'00"
38'00"
55'30"
59'30"
93'00"
118'30"
Retraction
0'00"
2'00"
3'00"
7'00"
9'00"
17'00"
20'00"
22'00"
25'00"
B
mito-GFP docking
No docking
18%
82%
n=413 protrusions
Occurrence of mito-GFP docking per axon (%)
***
Protrusion -forming
Random
Axon positions
C
Base of branch
Within branch
Branch tip
Presence of mito-GFP (%)
n=39
n=14
0-30
31-60
Branch lifetime (min)
D
Presence of mito-GFP (min)
n=45
n=8
5-15
>15
Max branch length (µm)
E
Presence of mito-GFP (min)
Branch lifetime (min)
F
Presence of mito-GFP (min)
Max branch length (µm)

Figure S2

Figure S2. Related to Figure 1. Mitochondria Localization at Specific Axonal Sites Correlates with Branching *in vivo*

(A) Time-lapse images illustrating mitochondria labeled with mito-GPF (white arrowheads) docking in RGC axons during branching. (Upper panel) A branch was formed (magenta arrows) shortly after the departure of the mitochondrion. The accumulation of mitochondria at specific axonal sites also promoted the formation of branches (cyan arrows). (Middle panel) Mitochondria moved into branches and to branch tips during protrusion stabilization. (Bottom panel) Branch retraction (yellow arrow) occurred shortly after mitochondria had moved out of the branch. (B) (Left panel) Proportion of protrusions with mitochondria docking at the base ≥1min preceding protrusion formation. (Right panel) Occurrence of mitochondria docking in protrusion-forming or random positions in the same axons (t_7=8.3, $p<0.0001$, paired t test). Red diamonds represent the averages. (C) Time of mitochondria present at different branch positions (normalized to branch lifetime) were higher in branches with longer lifetime (base of branch: U=146, p=0.0088; within branch: U=116, p=0.0002; branch tip: U=145, p=0.0004). (D) Time of mitochondria present at different branch positions were higher in branches with longer maximal branch length (base of branch: U=26.5, p<0.0001; within branch: U=46, p<0.0001; branch tip: U=95.5, p=0.0044). Error bars represent SEM. **p<0.01, ***p<0.001 (Mann Whitney test for C-D)). (E) Correlation between time of mitochondria present at different branch positions and lifetime of branch (base of branch: r=0.90; within branch: r=0.73; branch tip: r=0.53, p<0.0001 for all groups). (F) Correlation between time of mitochondria present at different branch positions and maximal branch length (base of branch: r=0.69, p<0.0001; within branch: r=0.70, p<0.0001; branch tip: r=0.34, p=0.013, Pearson's correlation for (E and F)). Scale bars, 5μm.

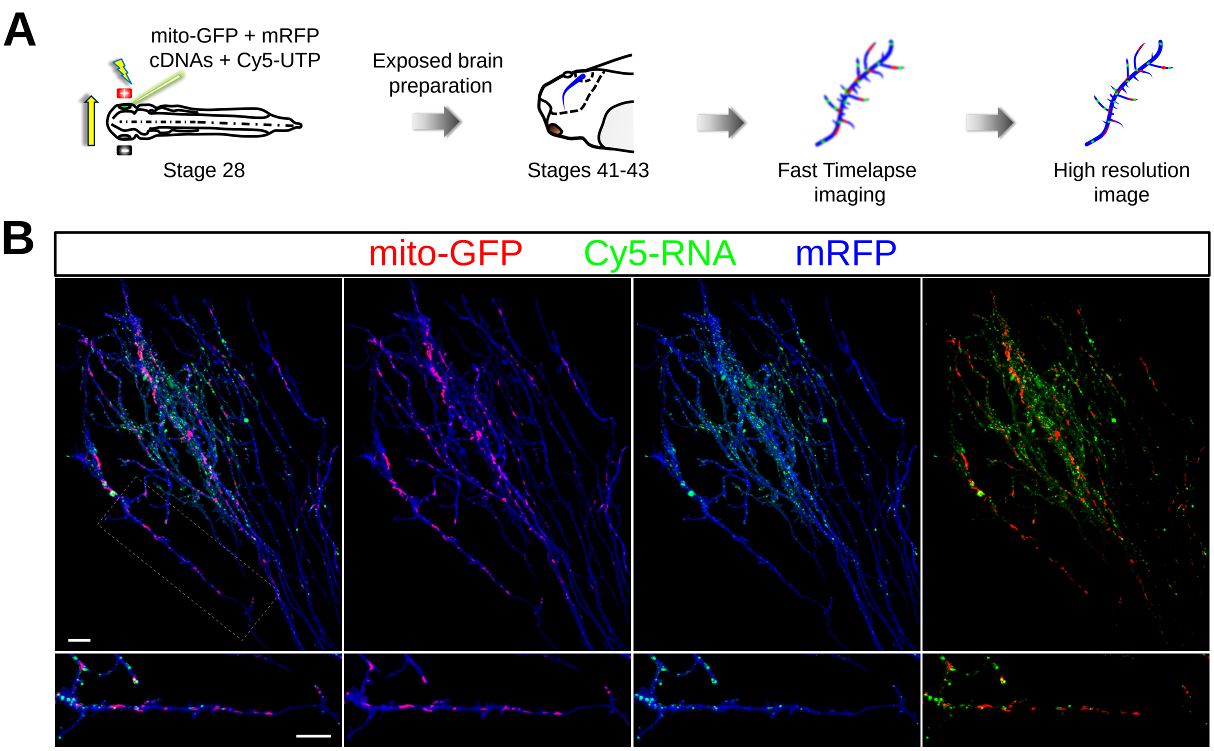

C

		Image scrambling		
Brain #	R(obs)	Iterations	R(rand) mean±sd	R(rand)>R(obs)
1	-0.016	1000	0.468±0.001	1000/1000
2	0.004	1000	0.504±0.001	1000/1000
3	0.113	1000	0.401±0.001	1000/1000
4	0.176	1000	0.368±0.001	1000/1000
5	0.048	1000	0.458±0.001	1000/1000
6	-0.094	1000	0.478±0.001	1000/1000
7	-0.039	1000	0.443±0.001	1000/1000
8	0.058	1000	0.367±0.001	1000/1000
9	-0.033	1000	0.456±0.001	1000/1000
10	0.051	1000	0.440±0.001	1000/1000
11	0.136	1000	0.420±0.001	1000/1000

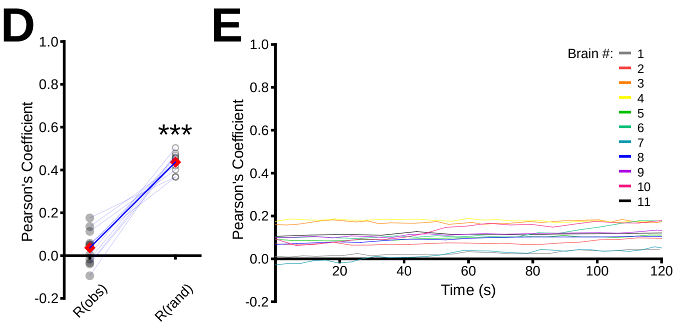

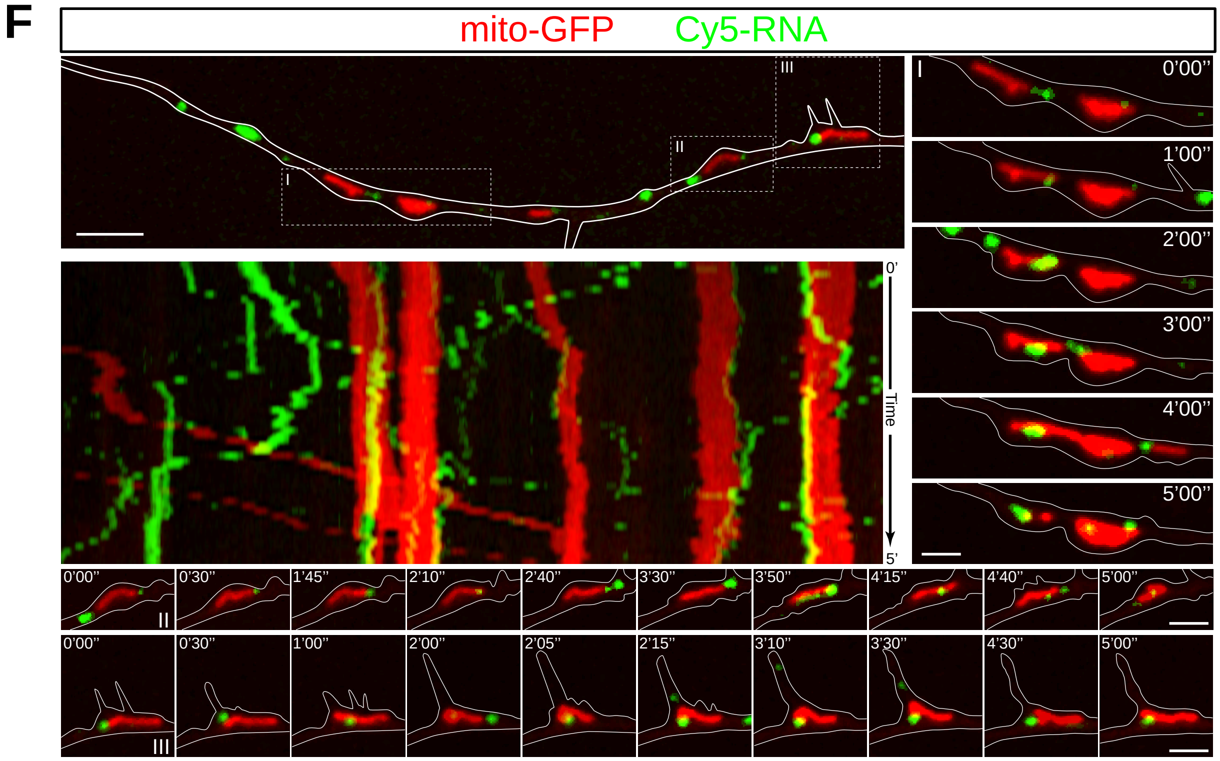

Figure S3

Figure S3. Related to Figure 1. Cy5-RNA and Mitochondria Are Distinct Structures

(A) Schematic of introducing Cy5-UTP, mito-GFP cDNA and mRFP cDNA into the RGCs with targeted eye electroporation. Fast *in vivo* time-lapse imaging of RNA granules, mitochondria and cell morphology was carried out for 2 minutes. This was followed by capturing a high resolution image stack of the axon arbors *in vivo*. (B) A high resolution image stack showing the localization of RNA granules and mitochondria in axon arbors. The RNA granules and mitochondria seldom showed colocalization but are often seen to juxtapose each other. (C) A table showing the Pearson's correlation coefficient (R) for Cy5-RNA and mito-GFP in the high resolution image stacks of 11 brains. Each image stack was individually scrambled to create 1000 random images. In all cases, the R(rand) yielded from the 1000 scrambled images were higher than the observed R(obs), robustly indicating that none of the samples showed a higher colocalization of the Cy5-RNA and mito-GFP than expected from chance. (D) The averages of R(rand) yielded from 1000 scrambled images were significantly higher than their R(obs) from the original high resolution image stacks (t_{10}=11.25, $p<0.0001$, paired t test), supporting that the Cy5-RNA and mitochondria are distinct structures. (E) A line graph illustrating the Costes Pearson's correlation coefficient remains low and similar across time from the time-lapse images (average image intervals= 5.8±0.6s per z-stack), supporting the observed low R(obs) in the high resolution image stack were not "artifacts" from a snapshot. (F) Time-lapse images showing the localizing of RNA granules and mitochondria in an RGC axon of eye explant. The kymograph and time series I, II and III show that Cy5-RNA and mitochondria are distinct structures and often juxtapose each other, reflecting potential interaction may exist. Error bars represent SEM. ***$p<0.001$. Scale bars, 10μm for (B), 5μm for (F) overview image and 2μm for (F) time series I, II and III.

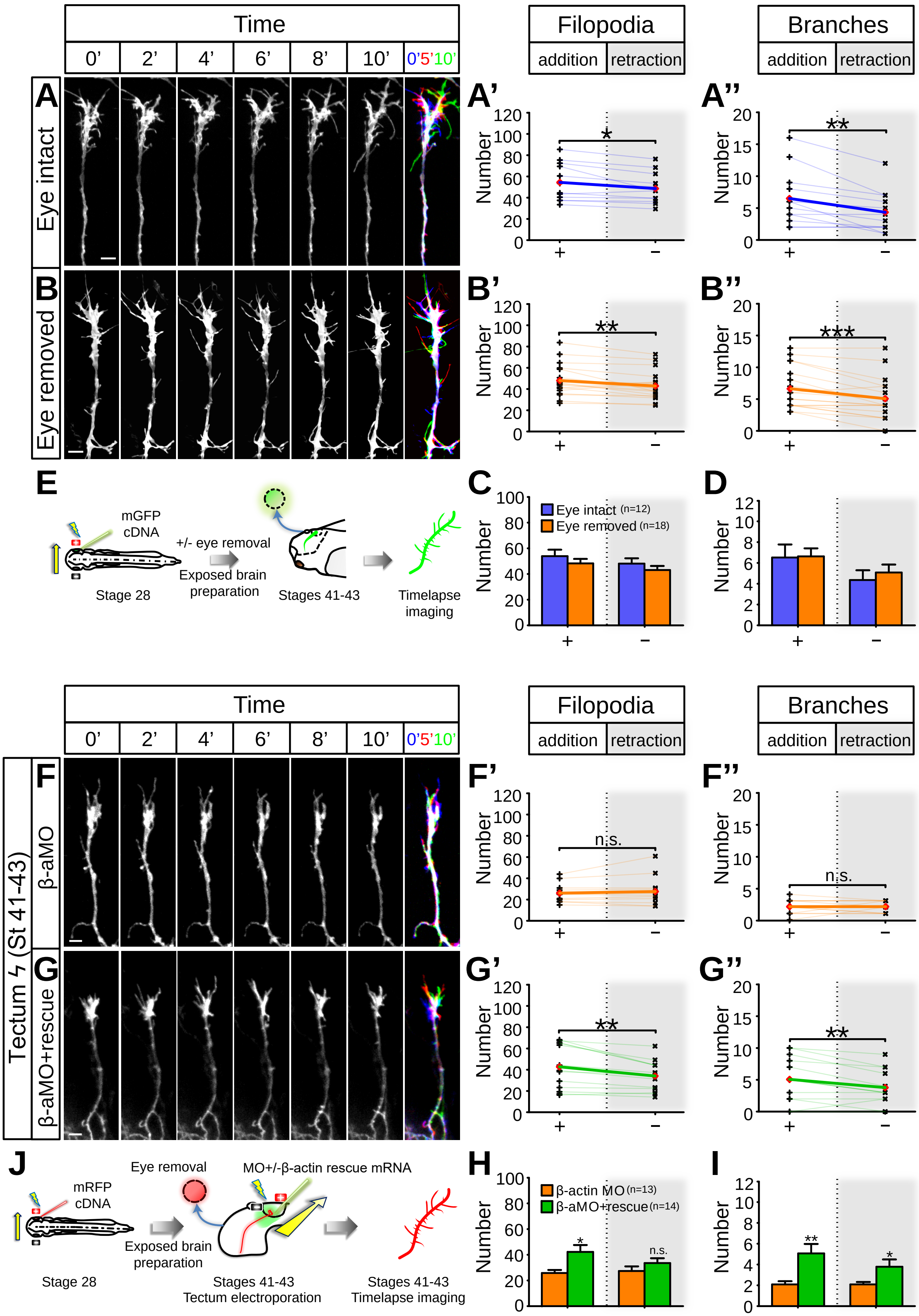
Time
0' 2' 4' 6' 8' 10' 0'5'10'
Filopodia
addition retraction
Branches
addition retraction
A
Eye intact
B
Eye removed
A'
A''
B'
B''
Number
*
**
***
E
mGFP cDNA
+/- eye removal
Exposed brain preparation
Stage 28
Stages 41-43
Timelapse imaging
C
D
Eye intact (n=12)
Eye removed (n=18)
F
β-aMO
G
β-aMO+rescue
Tectum ϟ (St 41-43)
F'
F''
G'
G''
n.s.
J
mRFP cDNA
Eye removal
MO+/-β-actin rescue mRNA
Exposed brain preparation
Stage 28
Stages 41-43 Tectum electroporation
Stages 41-43 Timelapse imaging
H
I
β-actin MO (n=13)
β-aMO+rescue (n=14)

Figure S4

Figure S4. Related to Figure 2 and 5. Axonal Branching Dynamics Remains Normal after Eye Removal and Branching Dynamics Defects Resulted from Local Inhibition of β-actin Translation are Rescued by Co-delivery of β-actin mRNA

(A and B) Time-lapse images of axonal branching in embryos with eye (soma of RGC axons) intact (A) and removed (B). (A' and A") Higher number of filopodia and branches were added than removed in embryos with eye intact (filopodia: t_{11}=3.1, p=0.01; branches: t_{11}=3.8, p=0.003). (B' and B") Higher number of filopodia and branches were added than removed in embryos with eye removed filopodia: t_{17}=3.4, p=0.003; branches: t_{17}=4.6, p=0.0002). (C and D) The addition and removal of filopodia (C; addition: t_{28}=0.95, p=0.35; removal: t_{28}=0.97, p=0.34) and branches (D; addition: t_{28}=0.08, p=0.94; removal: t_{28}=0.60, p=0.56) were unaffected after the removal of the eye. (E) Schematic of live imaging experiment on branching dynamics of RGC axons with eye intact/removed. (F and G) Time-lapse images of axon branching in the tectum after local delivery of β-actin MO (+/- β-actin rescue mRNA [0.5μM]) at stages 41-43. (F' and F") No significant differences were observed in the number of filopodia and branches that were added and removed after local delivery of β-actin MO (β-aMO) (addition: t_{12}=1.1, p=0.30; removal: t_{12}=0, p=1). (G' and G") The loss of the addition:removal biases of filopodia and branches after local delivery of β-aMO were rescued with local co-delivery of β-aMO resistant rescue mRNA. (filopodia: t_{13}=3.9, p=0.002; branches: t_{13}=3.3, p=0.005). (H) The addition of filopodia was rescued with β-actin rescue mRNA (t_{25}=2.7, p=0.01). Despite a trend of rescue was seen for the removal of filopodia, it was not statically significant (removal: t_{25}=1.2, p=0.24). (I) The addition and removal of filopodia and branches were rescued with β-actin rescue mRNA (D; addition: t_{25}=3.0, p=0.006; removal: t_{25}=2.2, p=0.04). (J) Schematic showing the local delivery of β-actin MO (+/- β-actin rescue mRNA) into RGC axons by tectum electroporation and imaged immediately thereafter. Electroporated eye was removed to eliminate somatic contribution of cytoplasmic material. Error bars represent SEM. *p<0.01, **p<0.01, ***p<0.001 (paired t test for (A, B, F and G) and unpaired t test for (C, D, H and I). Scale bars, 5μm. Red diamonds represent the averages (A, B, F and G).

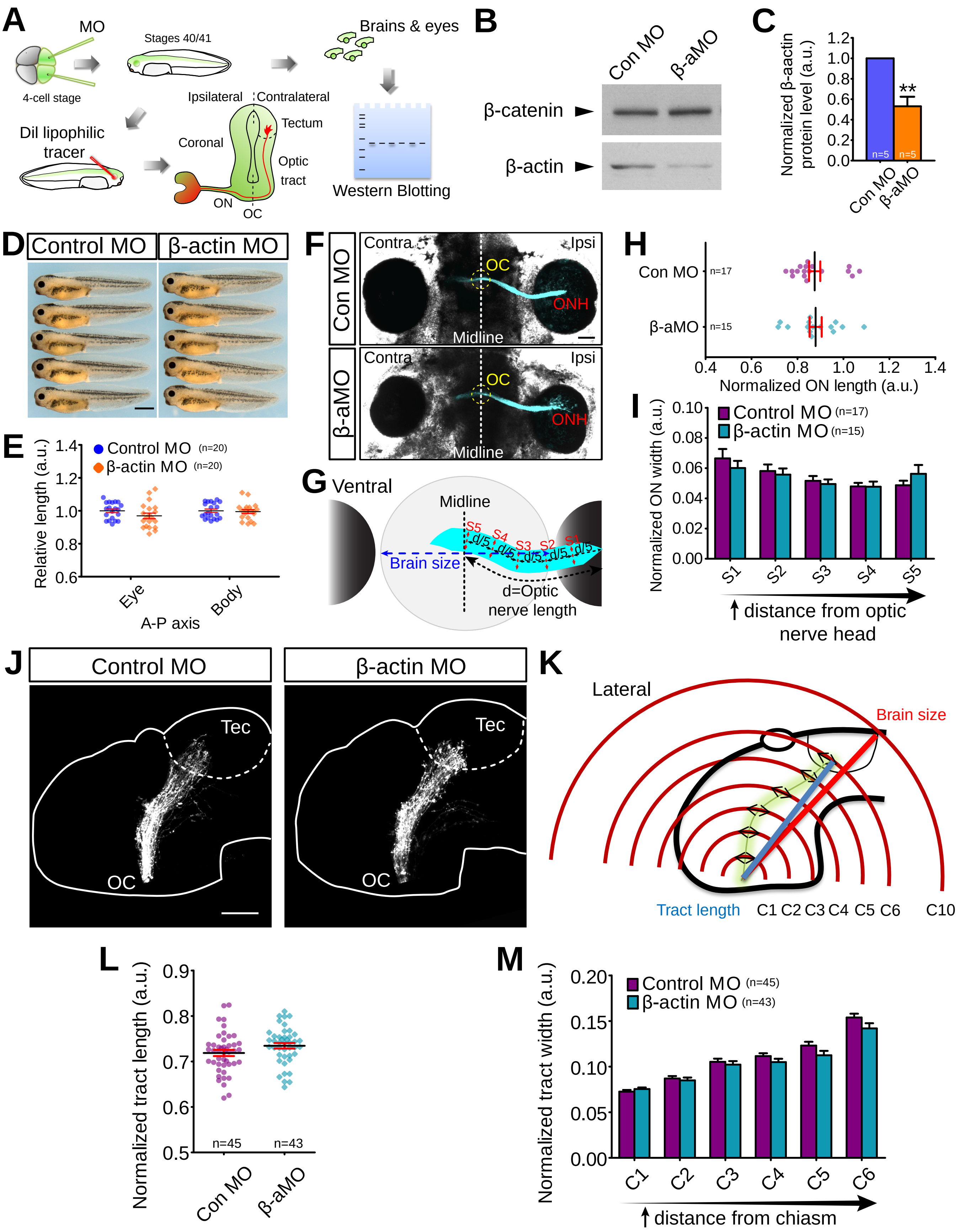
A
MO
4-cell stage
Stages 40/41
Brains & eyes
DiI lipophilic tracer
Ipsilateral
Contralateral
Tectum
Coronal
Optic tract
ON
OC
Western Blotting
B
Con MO
β-aMO
β-catenin
β-actin
C
Normalized β-aactin protein level (a.u.)
n=5
**
Con MO
β-aMO
D
Control MO
β-actin MO
E
Relative length (a.u.)
Control MO (n=20)
β-actin MO (n=20)
Eye
Body
A-P axis
F
Con MO
β-aMO
Contra
Ipsi
OC
ONH
Midline
G
Ventral
Midline
Brain size
d=Optic nerve length
H
Con MO
β-aMO
n=17
n=15
Normalized ON length (a.u.)
I
Normalized ON width (a.u.)
Control MO (n=17)
β-actin MO (n=15)
S1
S2
S3
S4
S5
distance from optic nerve head
J
Control MO
β-actin MO
Tec
OC
K
Lateral
Brain size
Tract length
C1 C2 C3 C4 C5 C6
C10
L
Normalized tract length (a.u.)
n=45
n=43
Con MO
β-aMO
M
Normalized tract width (a.u.)
Control MO (n=45)
β-actin MO (n=43)
C1
C2
C3
C4
C5
C6
distance from chiasm

Figure S5

Figure S5. Related to Figure 4. Embryo Remains Grossly Normal after β-actin Knockdown

(A) Schematic of MO injection into dorsal animal blastomeres at 4-cell stage. Injected embryos at Stage 41 were then used for anterograde tracing of RGC axons with lipophilic dye or protein harvesting of brain and eyes for western blot analysis. (B) Western blot showing β-actin was markedly reduced in the β-actin knockdown condition compared to control. β-catenin served as a loading control. (C) Normalized ratiometric measure of β-actin knockdown efficiency from western blot analyses (t_4=5.0, p=0.0073, paired t test). (D) Brightfield images showing that the morphology of β-actin morphants and their eyes being grossly normal. (E) Quantification of the length of eye (t_{38}=1.5, p=0.14) and body (t_{38}=0.3, p=0.76) in the anterior-posterior axis displaying no significant differences. (F) Images from ventral perspective of the brain showing the RGC axon bundles projecting from the optic nerve head (ONH) to the optic chiasm (OC) were indiscernible between control and β-actin morphants. (G) Schematic illustrating the quantification of the length and width of optic nerve. The optic nerve length (d) is defined by the length of the optic nerve (ON) from the ONH to the midline. The length was then divided into 5 sections (S1-S5), which are points where the width of the optic nerve was measured perpendicularly to the length axis. Both the length and width of the ON were then normalized to the brain size, which is defined by the straight line from the point of the brain adjacent to the center of the eye to the midline of the brain. (H) Scatter graph showing that the length of the ON from the ONH to the midline did not differ between the control and β-actin morphants (t_{30}=0.1, p=0.91). (I) The ON width didnot differ between the control and β-actin morphants across the length of the ON from the ONH to the midline (S1: t_{30}=0.8, p=0.43; S2: t_{30}=0.4, p=0.70; S3: t_{30}=0.5, p=0.64; S4: t_{30}=0.04, p=0.97; S5: t_{30}=1.2, p=0.24). (J) Images from the lateral perspective of the contralateral hemisphere of brain showing that retinotectal projection from the OC to the optic tectum were indiscernible between control and β-actin morphants. (K) Schematic illustrating the quantification of the length and width of optic tract. The optic tract length is defined by a straight line from the OC to the distal axon terminals of the optic tract. Ten equally spaced concentric circles (C1-C10) were overlaid on the tract images with the center of the circles overlying the OC and C10 overlaying the dorsal-posterior tectal boundary. Both the length and width of tract were then normalized to the brain size, which is defined by the straight line from the OC to the dorsal-posterior tectal boundary. (L) Scatter graph showing that the length of the optic tract did not differ between the control and β-actin morphants (t_{86}=1.7, p=0.086). (M) The optic tract width did not differ between the control and β-actin morphants across the length of the optic tract (C1: t_{86}=1.1, p=0.27; C2: t_{86}=0.5, p=0.62; C3: $t8_6$=0.6, p=0.56; C4: t_{86}=1.4, p=0.17; C5: t_{86}=1.7, p=0.10; C6: t_{86}=1.7, p=0.092). Error bars represent SEM. **p<0.01 (unpaired t test for (E, H, I, L and M)). Scale bars: 1mm for (D) and 100μm for (F and J).

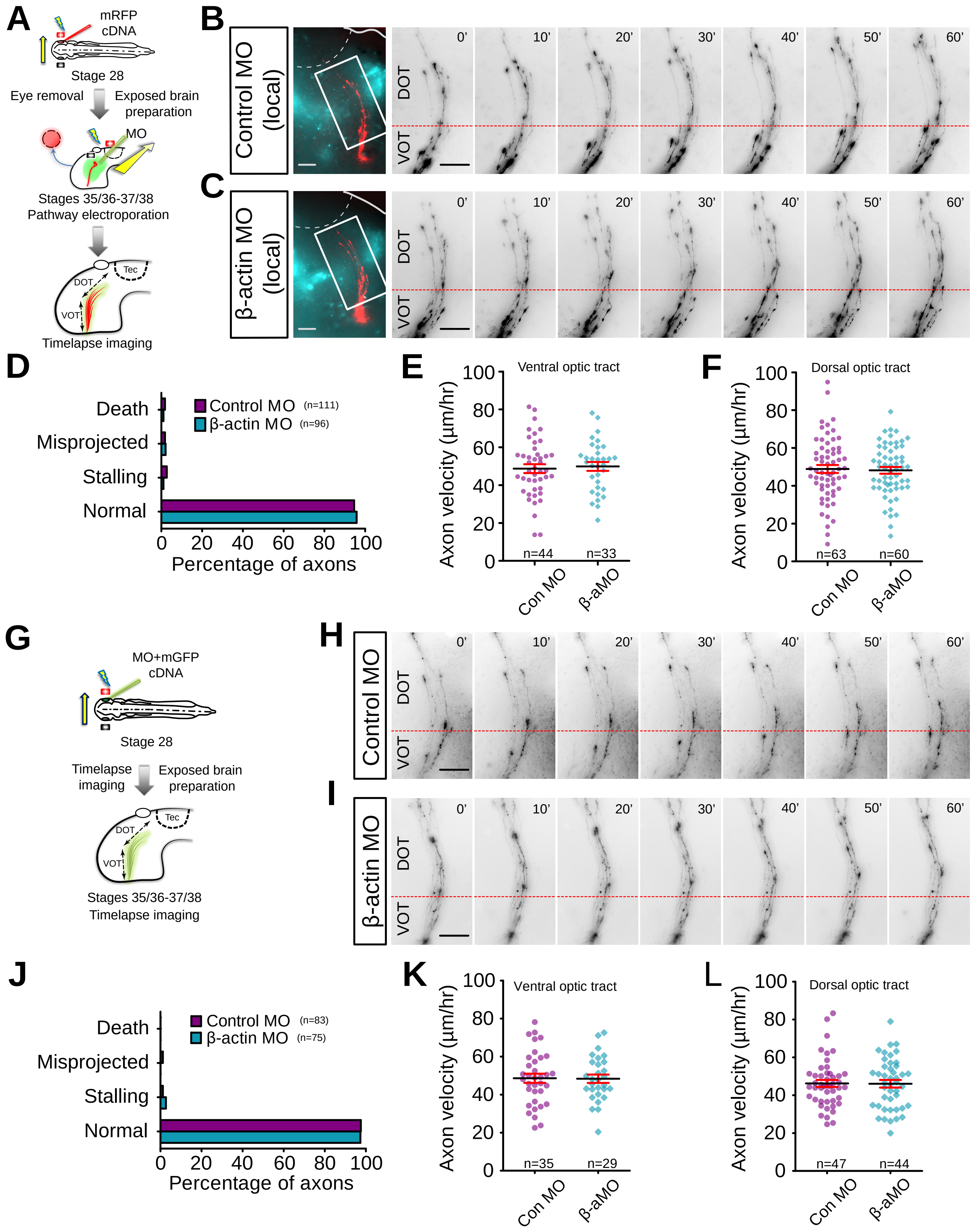
A
mRFP cDNA
Stage 28
Eye removal
Exposed brain preparation
MO
Stages 35/36-37/38
Pathway electroporation
Tec
DOT
VOT
Timelapse imaging
B
Control MO (local)
C
β-actin MO (local)
0'
10'
20'
30'
40'
50'
60'
D
Death
Misprojected
Stalling
Normal
Control MO (n=111)
β-actin MO (n=96)
Percentage of axons
E
Ventral optic tract
Axon velocity (μm/hr)
n=44
n=33
Con MO
β-aMO
F
Dorsal optic tract
n=63
n=60
G
MO+mGFP cDNA
Stage 28
Timelapse imaging
Exposed brain preparation
Stages 35/36-37/38
Timelapse imaging
H
Control MO
I
β-actin MO
J
Control MO (n=83)
β-actin MO (n=75)
K
Ventral optic tract
n=35
n=29
L
Dorsal optic tract
n=47
n=44

Figure S6

Figure S6. Related to Figure 5. Local Inhibition of β-actin Translation Does Not Grossly Affect Axon Navigation in the Optic Tract

(A) Schematic showing the local delivery of MO into RGC axons by pathway electroporation. The eye electroporated with mRFP plasmids was removed to eliminate somatic contribution of cytoplasmic material. The embryos were imaged immediately after local delivery of MO to ensure any observed effect would be a result of acute blockage of local β-actin translation. (B and C) Time-lapse images of RGC axons navigating through the ventral optic tract (VOT) and dorsal optic tract (DOT) after local Con MO (B) or β-actin MO (C) delivery. MO is shown in cyan and axons are shown in red on the left. (D) Axon behaviors during navigation were unaffected in axons with local β-actin MO delivery (death: p=1; misprojected: p=1; stalling: p=0.63; normal: p=0.75). (E and F) Axon navigation velocities in both the VOT (E) and DOT (F) were unaffected local β-actin MO delivery. (VOT: t_{75}=0.3, p=0.73; DOT: t_{121}=0.3, p=0.80). (G) Schematic showing eye electroporation and live imaging of axon navigation. (H and I) Time-lapse images of axon navigation through the ventral optic tract (VOT) and dorsal optic tract (DOT) of Con MO- and β-actin MO-positive RGC axons. (J) Axon behaviors during navigation were unaffected in axons from the β-actin MO condition. (misprojected: p=1 stalling: p=0.6; normal: p=1)(K and L) Axon navigation velocities in both the VOT (K) and DOT (L) were unaffected after β-actin knockdown in RGCs (VOT: t_{62}=0.07, p=0.94; DOT: t_{89}=0.09, p=0.95). Error bars represent SEM. (Fisher's exact test for (D and J)). (unpaired t test for (E, F, K and L)). Scale bars, 50μm for (B, C, H and I).

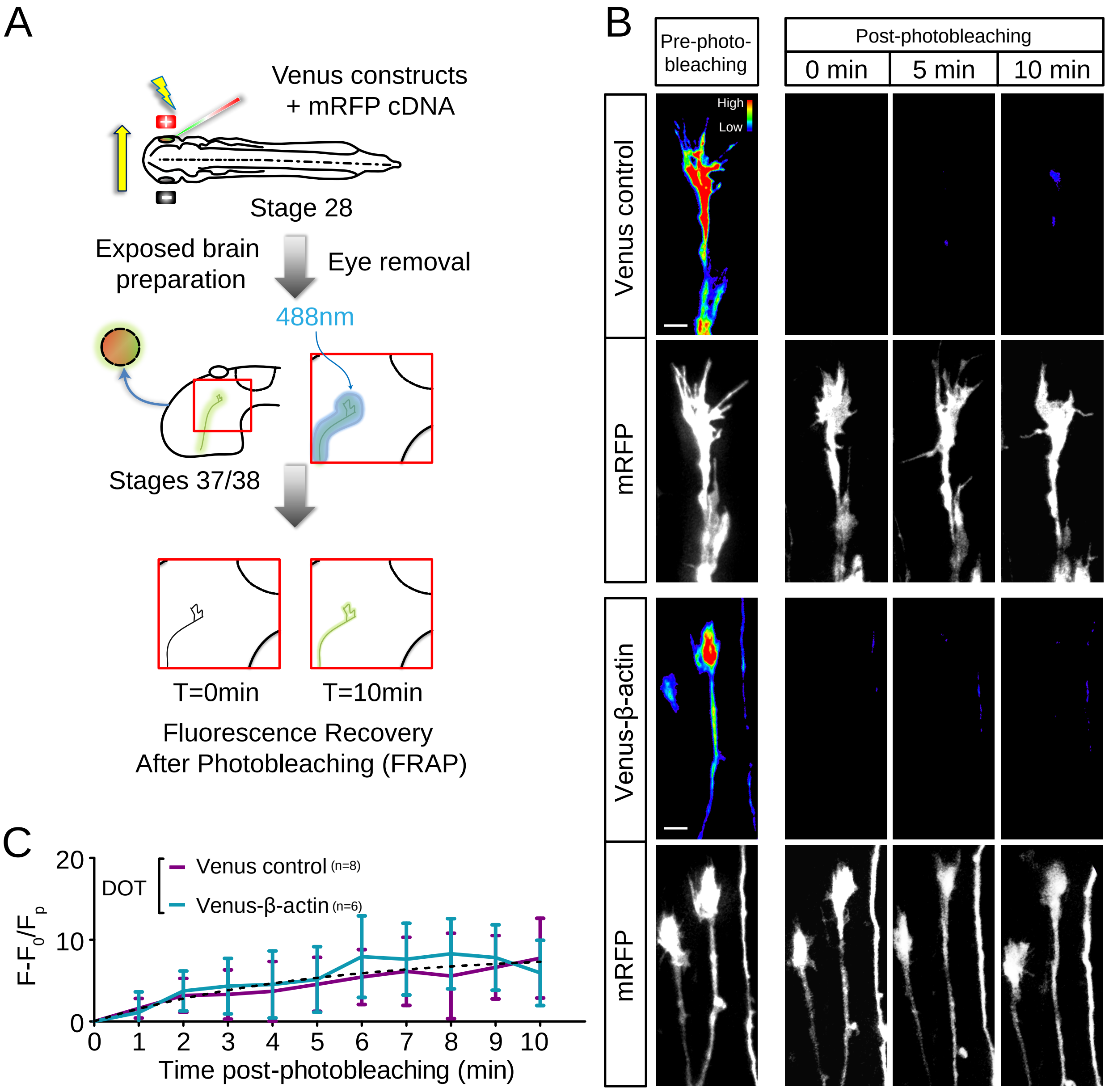
A
Venus constructs
+ mRFP cDNA
Stage 28
Exposed brain
preparation
Eye removal
488nm
Stages 37/38
T=0min
T=10min
Fluorescence Recovery
After Photobleaching (FRAP)
B
Pre-photo-
bleaching
Post-photobleaching
0 min
5 min
10 min
High
Low
Venus control
mRFP
Venus-β-actin
mRFP
C
DOT
Venus control (n=8)
Venus-β-actin (n=6)
F-F0/Fp
20
10
0
0
1
2
3
4
5
6
7
8
9
10
Time post-photobleaching (min)

Figure S7

Figure S7. Related to Figure 6. Local β-actin Synthesis Is Not Detected in Growth Cones Navigating the Optic Tract

(A) Experimental design of fluorescence recovery after photobleaching experiment of Venus-constructs in the optic tract *in vivo*. Electroporated eye was removed to eliminate somatic contribution of cytoplasmic material. OT, optic tract. (B) Fluorescence heatmaps illustrating that limited recoveries were detected with both the Venus control and Venus-β-actin constructs. (C) Line graph showing the recovery of Venus signals after photobleaching over the course of 10 minutes. Dotted line represents least-squares fit to a single-exponential function; note that a single model is highly adequate for fitting both conditions (Venus control vs. Venus-β-actin: $F_{3,148}$=0.15, p=0.93, Extra sum-of-squares F test). Error bars represent SEM. Scale bars, 5μm.
