# Supplementary figures and images for: RNA Docking and Local Translation Regulate Site-Specific Axon Remodeling In Vivo
Source: Neuron. 2017 Aug 16;95(4):852–868.e8. doi: 10.1016/j.neuron.2017.07.016 (PMC5563073; doi:10.1016/j.neuron.2017.07.016)

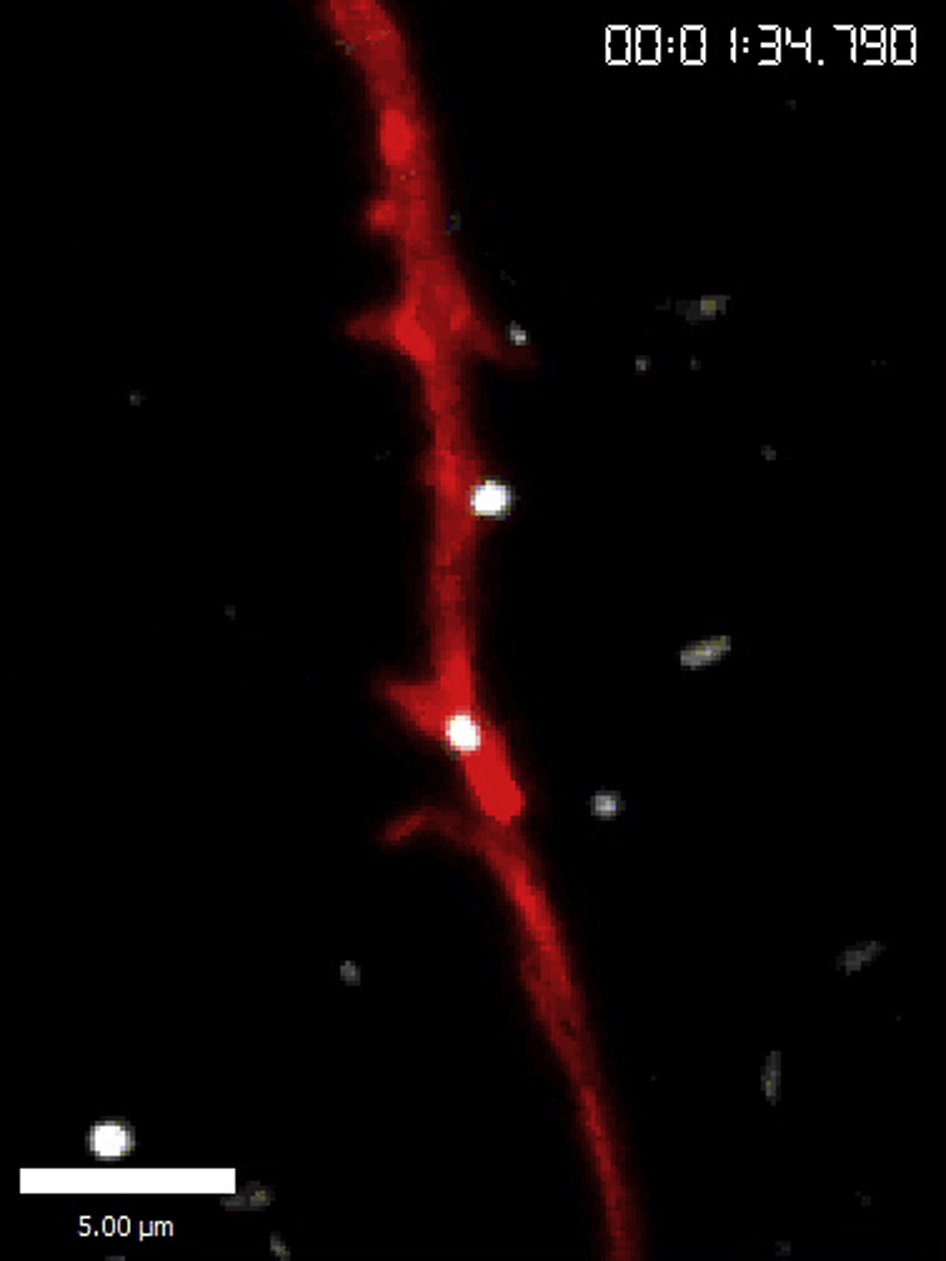

Supplement: Movie S1. Related to Figure 1. RNA granules dock at axonal sites in advance of branch emergence in vivo — The movie was captured at 3.3 seconds per frame (spf) and the rate of movie is 40 frames per second (fps). [file mmc2.jpg]

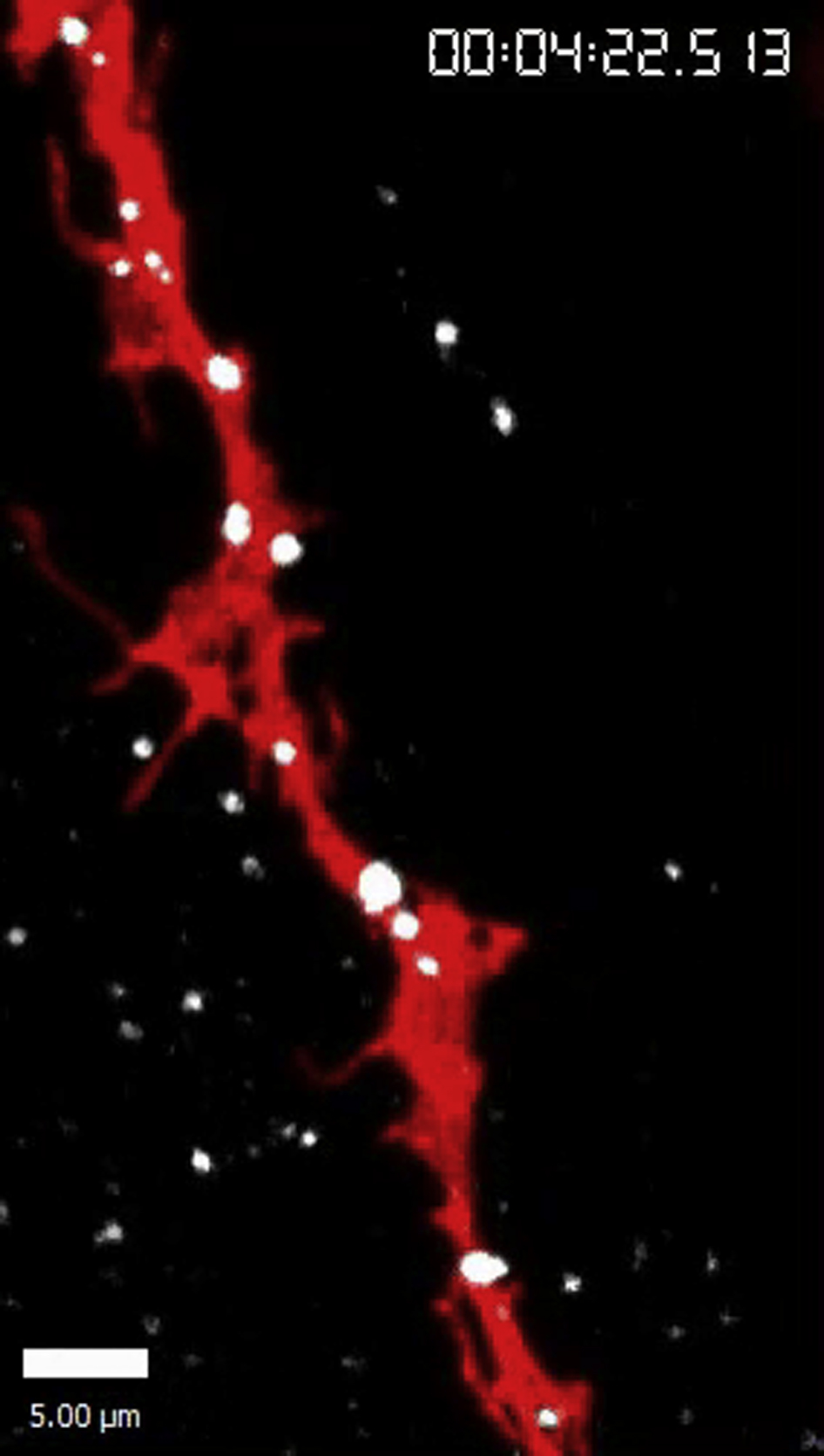

Supplement: Movie S2. Related to Figure 1. RNA granules invade stabilizing branches in vivo — The movie was captured at 3.8spf and the rate of movie is 100 fps. [file mmc3.jpg]

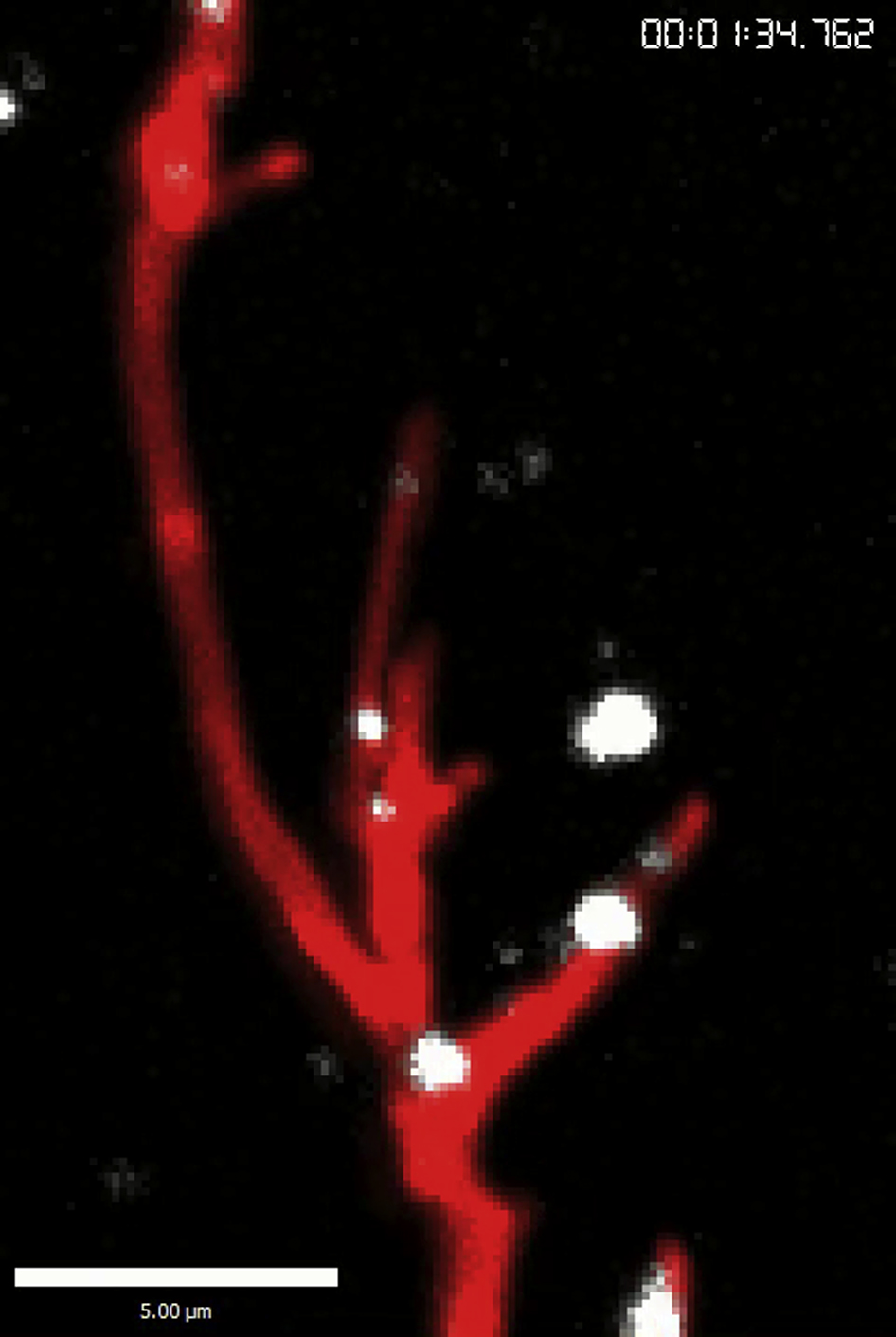

Supplement: Movie S3. Related to Figure 1. Trafficking of RNA granules out of branch before branch retraction in vivo — The movie was captured at 5.6 spf and the rate of movie is 30 fps. [file mmc4.jpg]

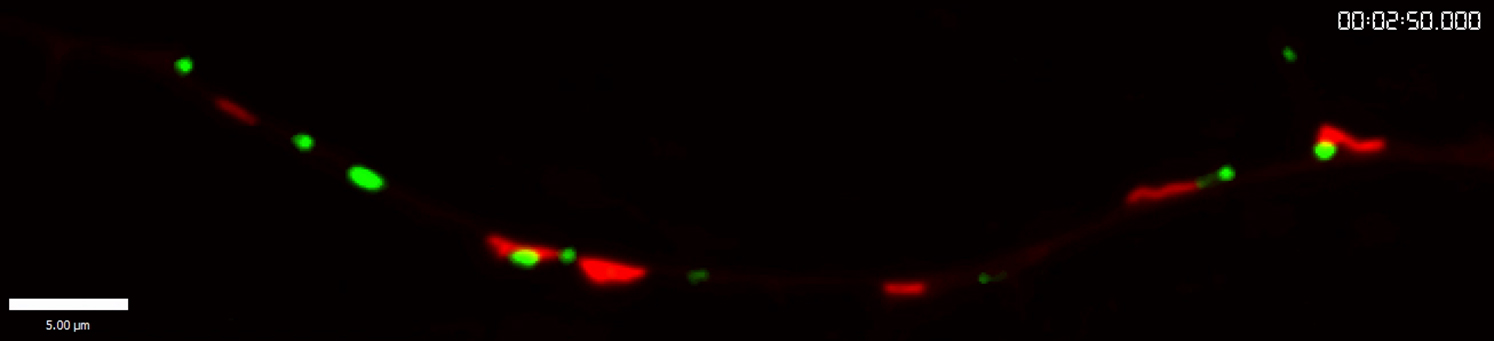

Supplement: Movie S4. Related to Figure 1, S1, S2 and S3. RNA granules and mitochondria dynamics — The movie was captured at 5 spf and the rate of movie is 10 fps. [file mmc5.jpg]

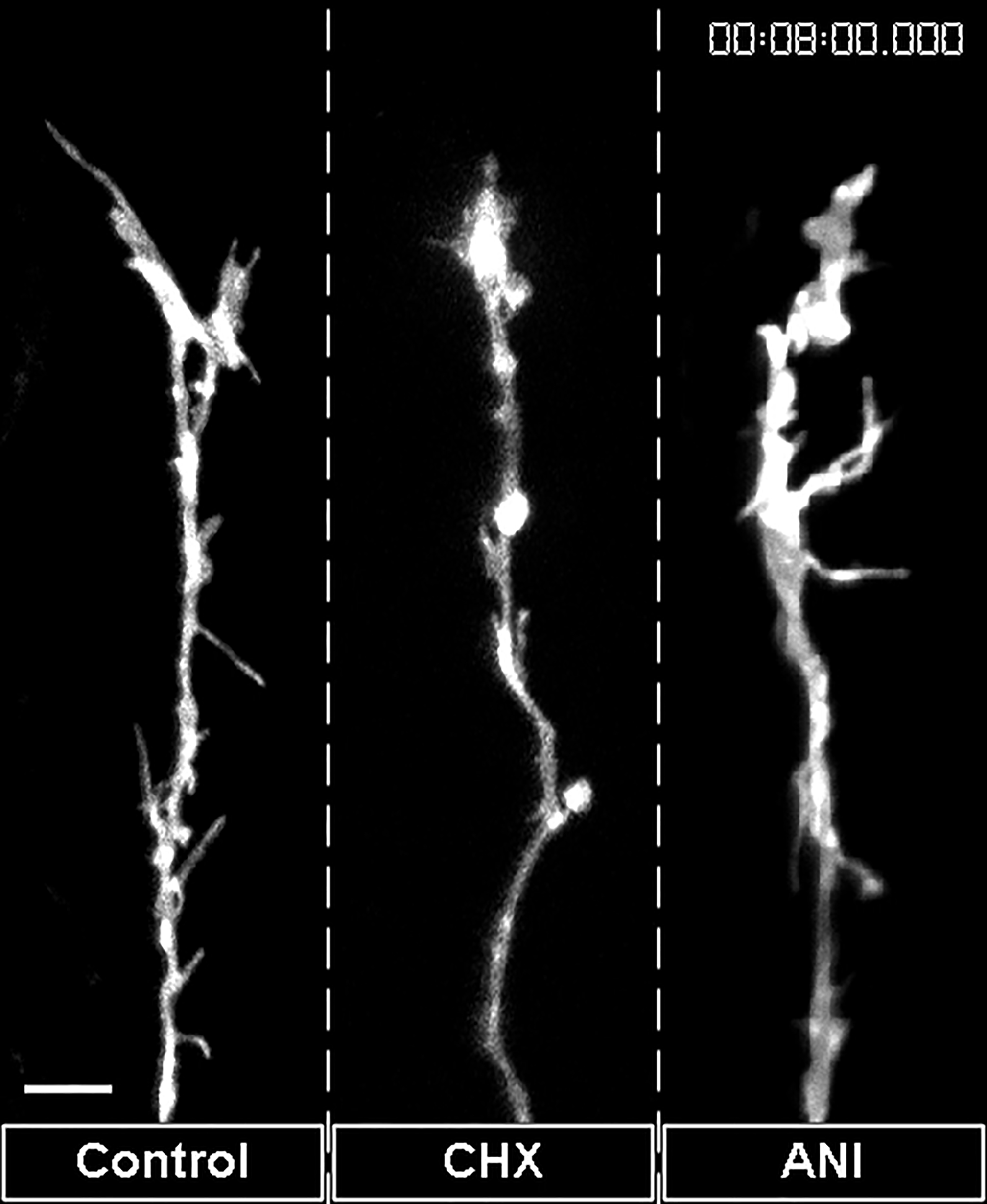

Supplement: Movie S5. Related to Figure 2. Acute translation inhibition reduces branching dynamics of somaless RGC axons in vivo — The movie was captured at 30 spf and the rate of movie is 8 fps. [file mmc6.jpg]

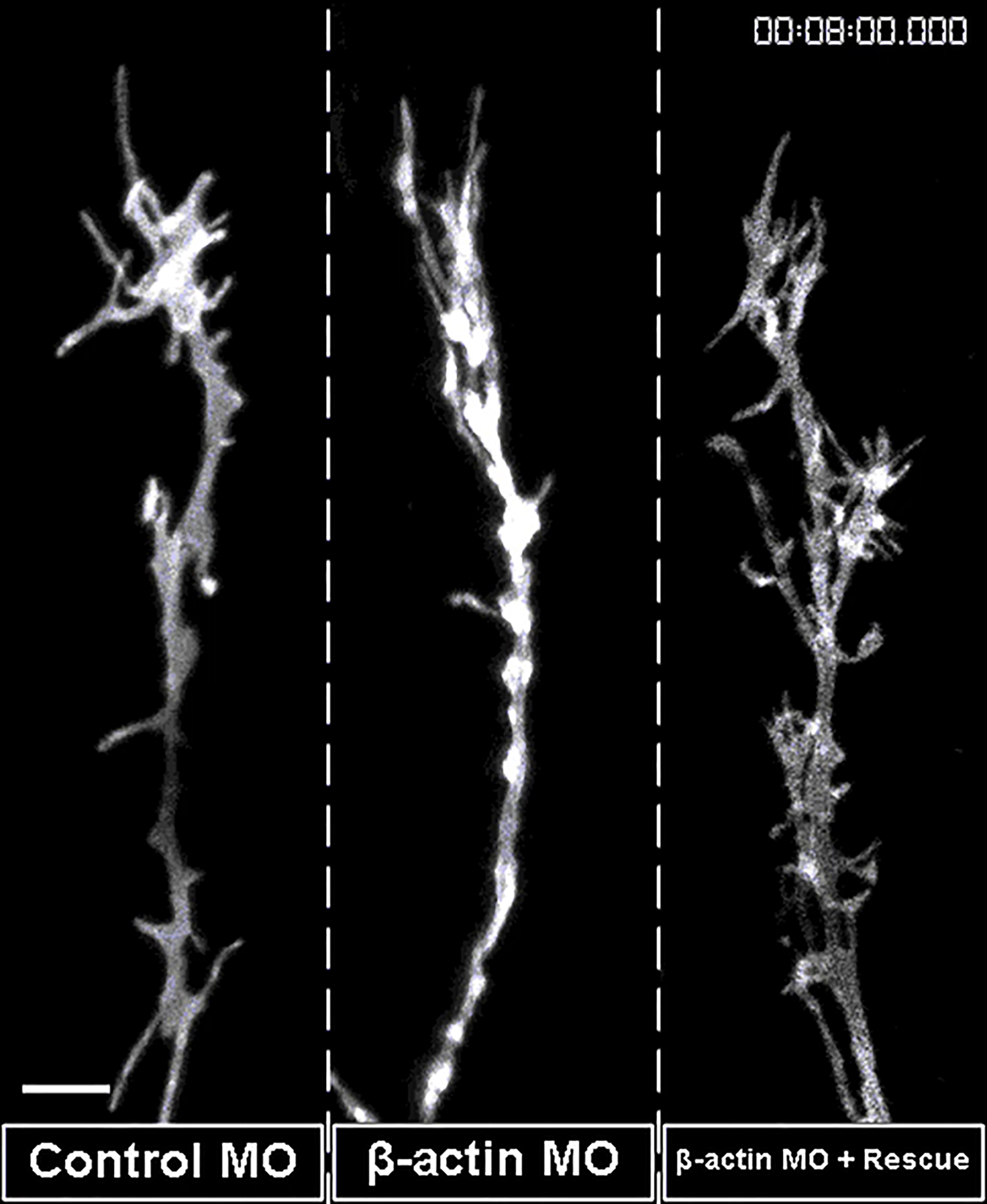

Supplement: Movie S6. Related to Figure 4. β-actin knockdown inhibits branching dynamics of RGC axons in vivo — The movie was captured at 30 spf and the rate of movie is 8 fps. [file mmc7.jpg]

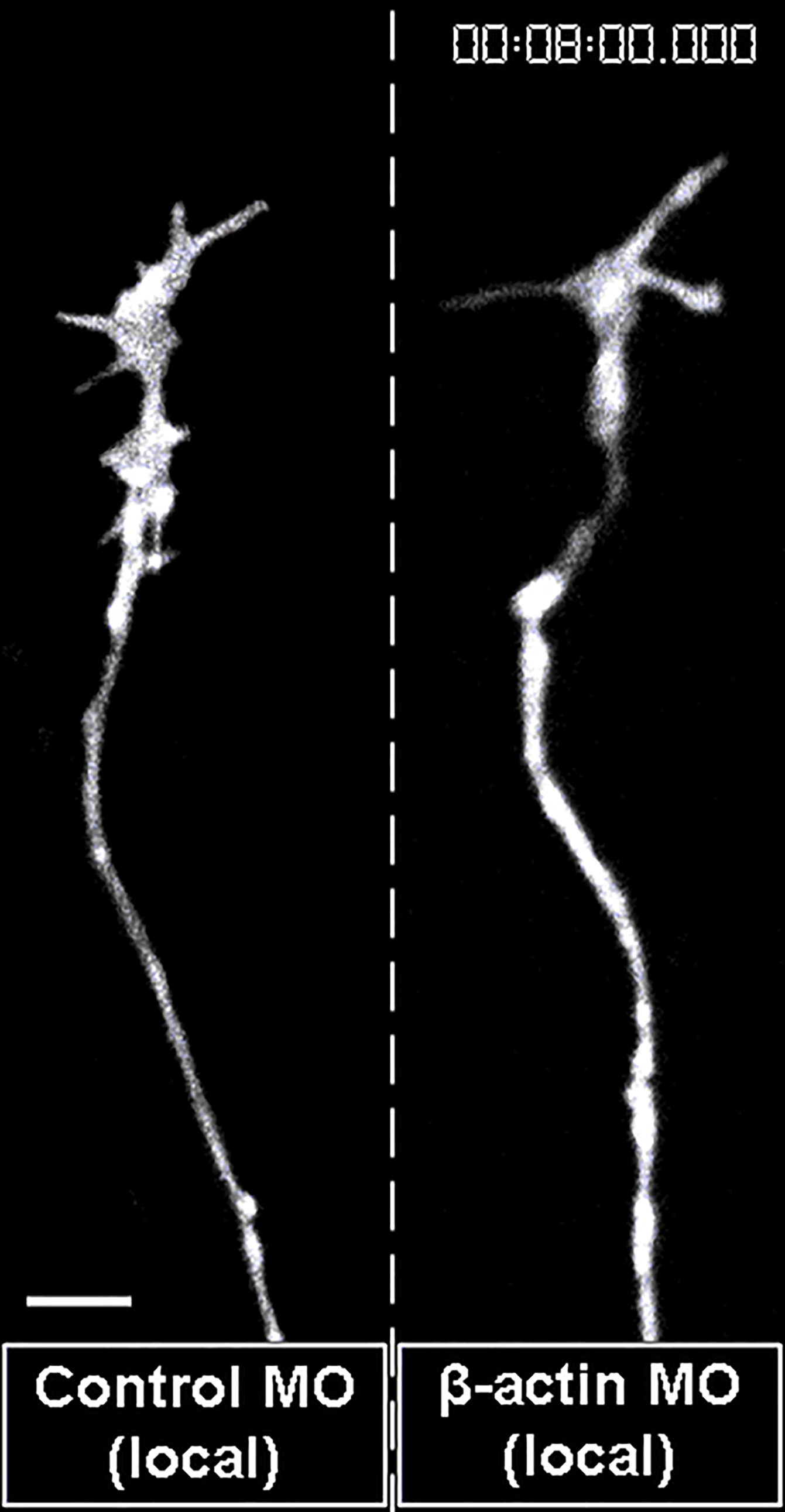

Supplement: Movie S7. Related to Figure 5. Local inhibition of β-actin translation inhibits branching dynamics in somaless RGC axons in vivo — The movie was captured at 30 spf and the rate of movie is 8 fps. [file mmc8.jpg]

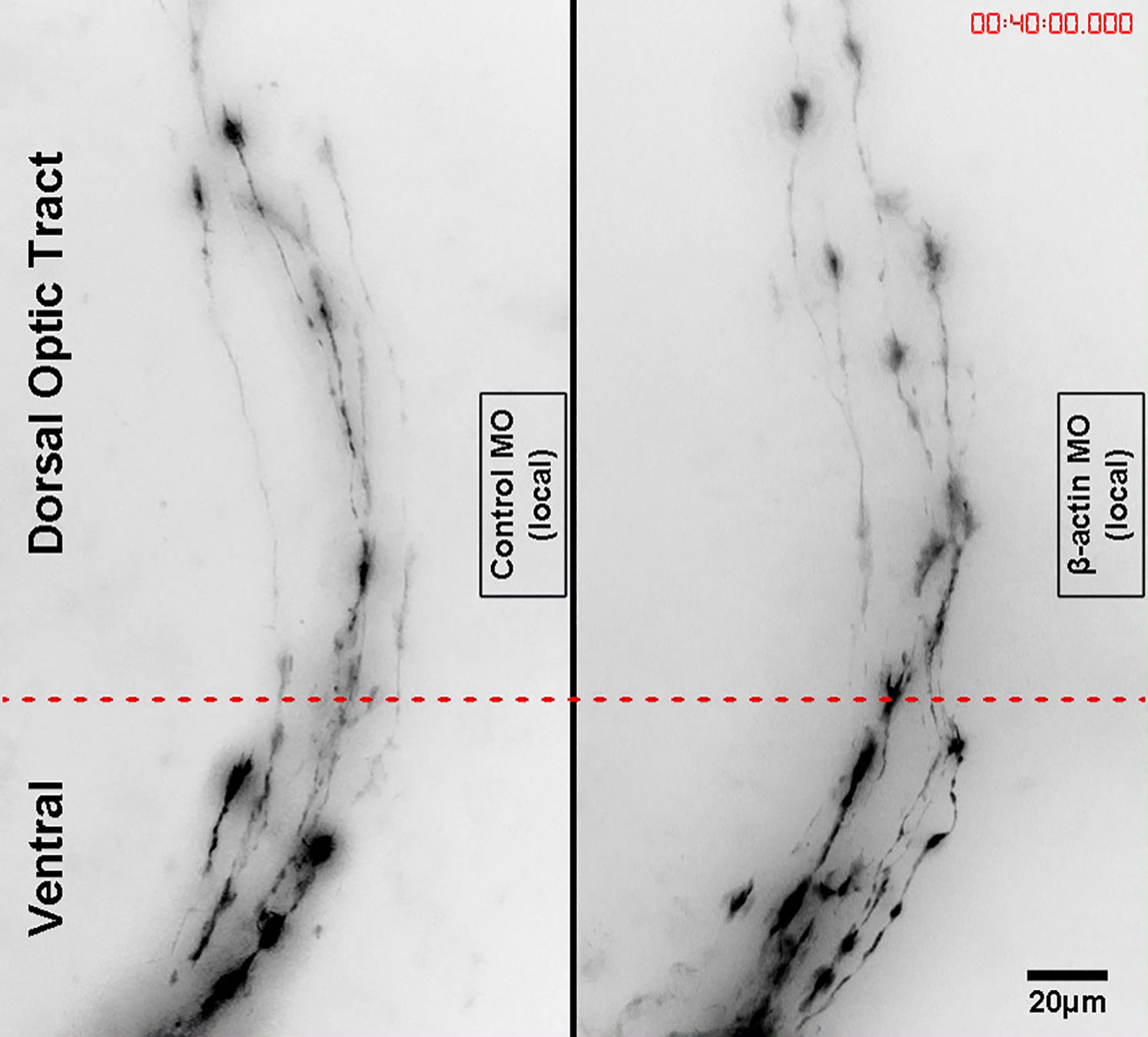

Supplement: Movie S8. Related to Figure 5 and S6. Local inhibition of β-actin translation does not result in observable defects in RGC axon navigation in vivo — The movie was captured at 300 spf and the rate of movie is 6 fps. [file mmc9.jpg]

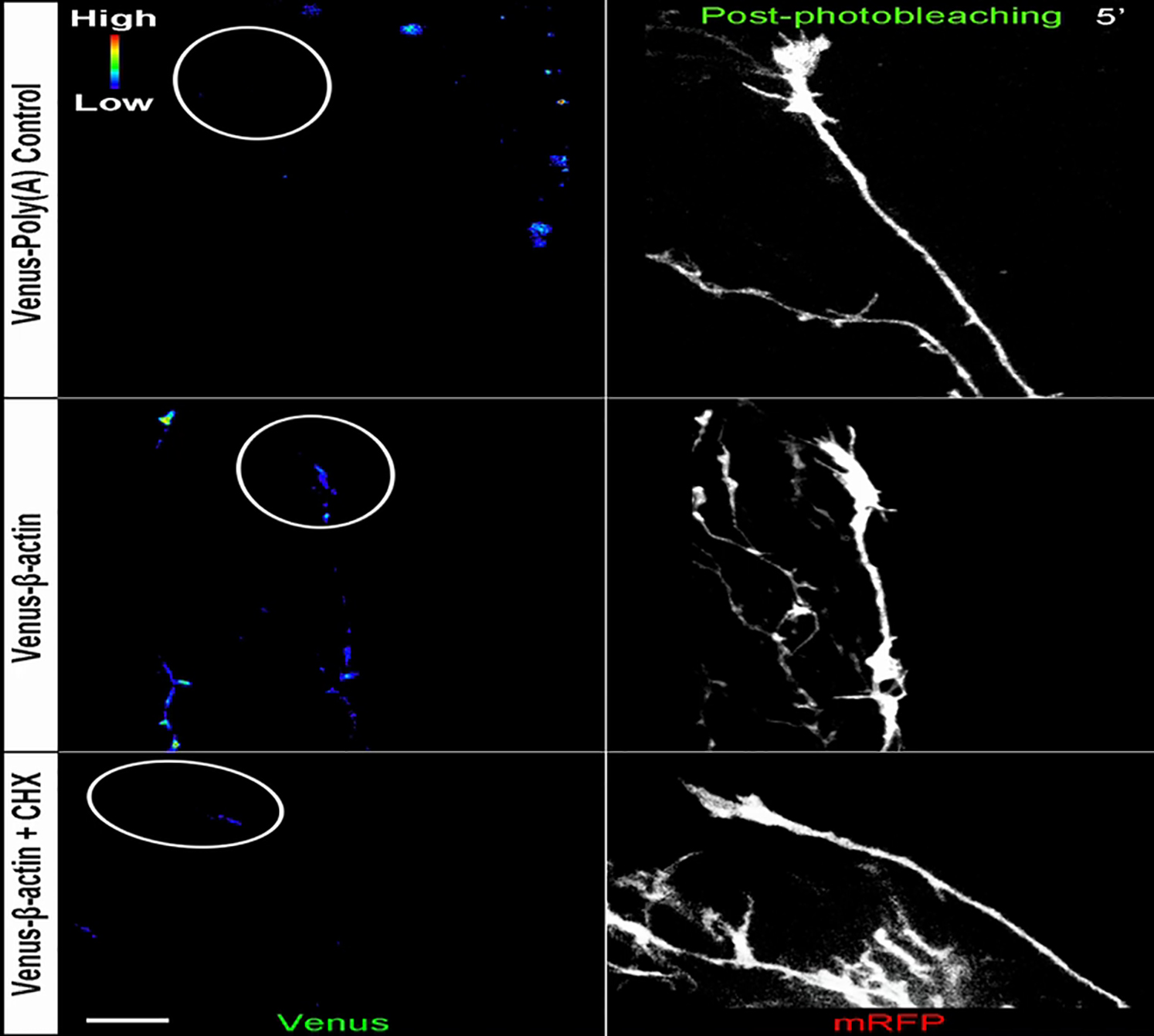

Supplement: Movie S9. Related to Figure 6. FRAP of Venus control and Venus-β-actin in RGC axon terminals in vivo — The movie was created at 60 spf and played at the rate of 2 fps. [file mmc10.jpg]

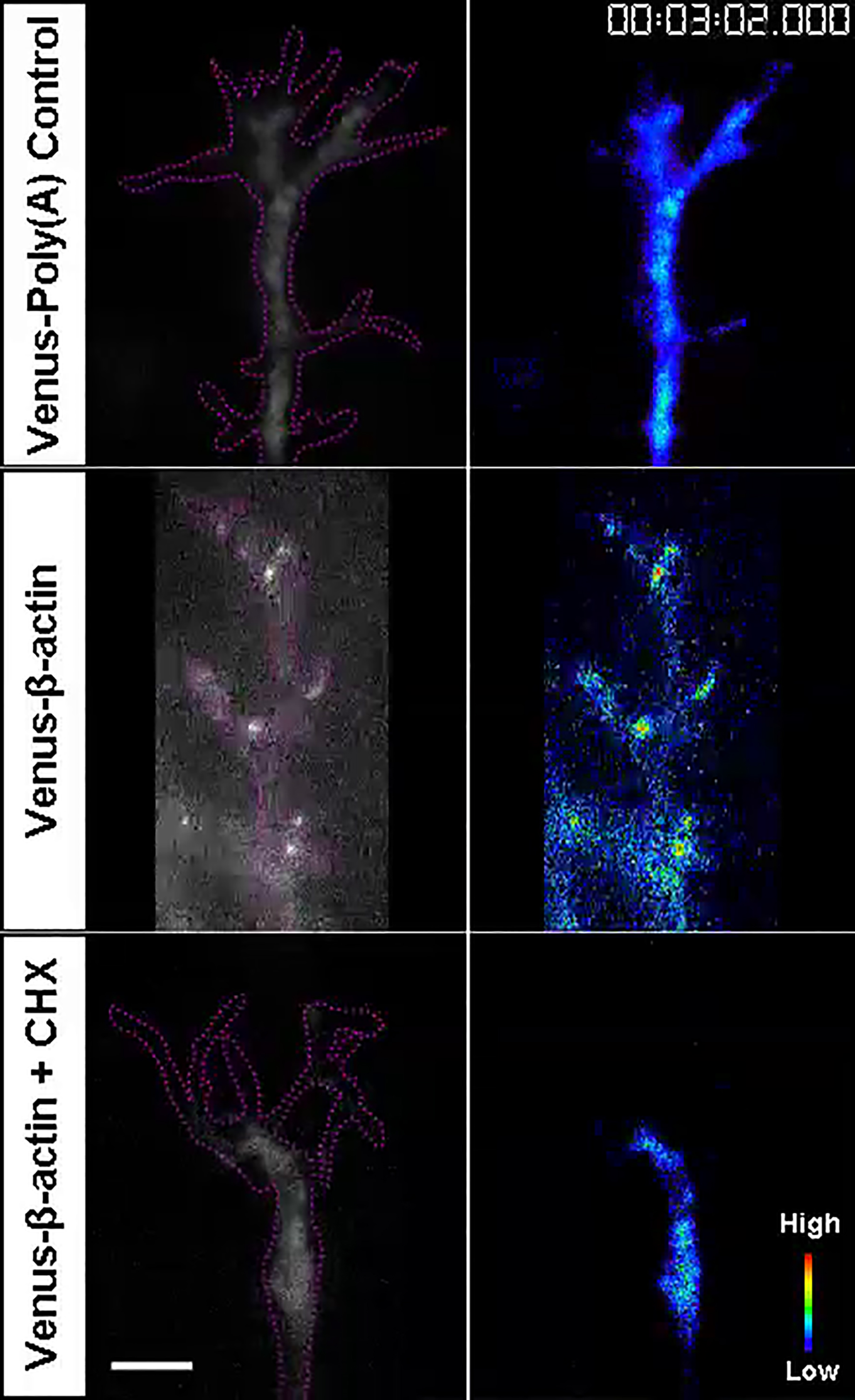

Supplement: Movie S10. Related to Figure 7. Focal translation of Venus-B-actin promotes hotspot formation at branches in vivo — The movie was captured at 1 spf and the rate of movie is 60 fps. [file mmc11.jpg]

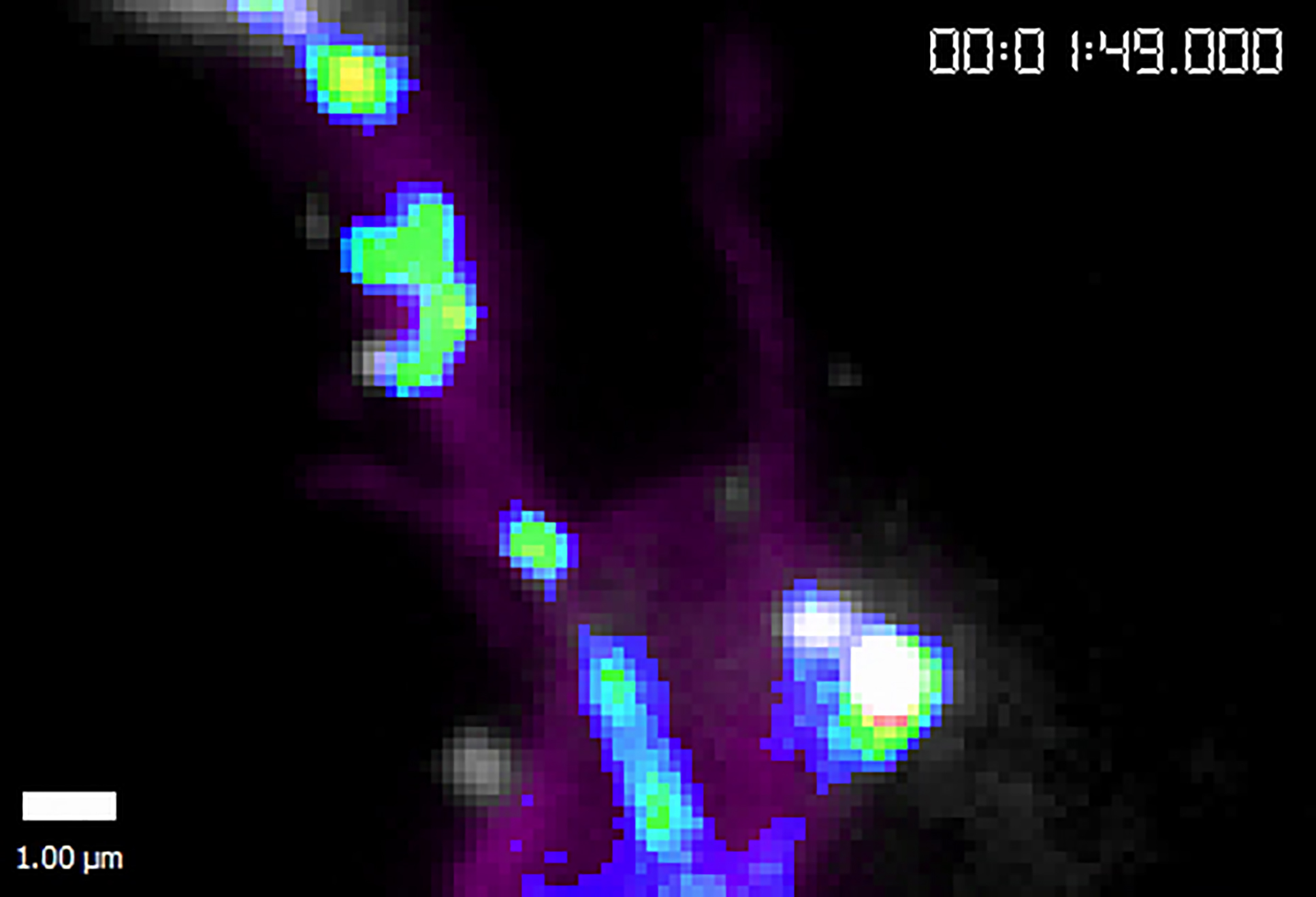

Supplement: Movie S11. Related to Figure 8. Focal translation of Venus-B-actin takes place in close proximity to docked RNA granules in vivo — The movie was captured at 1 spf and the rate of movie is 30 fps. [file mmc12.jpg]
