# Supplementary material for: RNA Docking and Local Translation Regulate Site-Specific Axon Remodeling In Vivo
Source: Neuron. 2017 Aug 16;95(4):852–868.e8. doi: 10.1016/j.neuron.2017.07.016 (PMC5563073; doi:10.1016/j.neuron.2017.07.016)
Supplement: Document S2. Article plus Supplemental Information [file mmc13.pdf]

Article

Neuron

RNA Docking and Local Translation Regulate Site-Specific Axon Remodeling In Vivo

Highlights

- Tracking endogenous RNA shows that RNA docking predicts axon branch emergence in vivo
- Axon arbor complexity in vivo depends on local protein synthesis
- Axonal β-actin synthesis regulates branching by increased branch initiation
- Live imaging reveals de novo synthesis of β-actin hotspots during branch formation

Authors

Hovy Ho-Wai Wong, Julie Qiaojin Lin, Florian Ströhl, ..., William A. Harris, Clemens F. Kaminski, Christine E. Holt

Correspondence

ceh33@cam.ac.uk

In Brief

Wong et al. track endogenous RNA granules in single axons in vivo and show that they predict positions of axonal branch formation in the tectum. They also show that perturbation of local protein synthesis in these axons autonomously regulates branch formation.

Wong et al., 2017, Neuron *95*, 852–868
August 16, 2017 © 2017 The Authors. Published by Elsevier Inc.
http://dx.doi.org/10.1016/j.neuron.2017.07.016

Neuron
Article

RNA Docking and Local Translation Regulate Site-Specific Axon Remodeling In Vivo

Hovy Ho-Wai Wong,[1] Julie Qiaojin Lin,[1,3] Florian Ströhl,[2,3] Cláudio Gouveia Roque,[1] Jean-Michel Cioni,[1] Roberta Cagnetta,[1] Benita Turner-Bridger,[1] Romain F. Laine,[2] William A. Harris,[1] Clemens F. Kaminski,[2] and Christine E. Holt[1,4,*]
[1]Department of Physiology, Development and Neuroscience, University of Cambridge, Cambridge CB2 3DY, UK
[2]Department of Chemical Engineering and Biotechnology, University of Cambridge, Cambridge CB3 0AS, UK
[3]These authors contributed equally
[4]Lead Contact
*Correspondence: ceh33@cam.ac.uk
http://dx.doi.org/10.1016/j.neuron.2017.07.016

SUMMARY

Nascent proteins can be positioned rapidly at precise subcellular locations by local protein synthesis (LPS) to facilitate localized growth responses. Axon arbor architecture, a major determinant of synaptic connectivity, is shaped by localized growth responses, but it is unknown whether LPS influences these responses in vivo. Using high-resolution live imaging, we examined the spatiotemporal dynamics of RNA and LPS in retinal axons during arborization in vivo. Endogenous RNA tracking reveals that RNA granules dock at sites of branch emergence and invade stabilized branches. Live translation reporter analysis reveals that de novo β-actin hotspots colocalize with docked RNA granules at the bases and tips of new branches. Inhibition of axonal β-actin mRNA translation disrupts arbor dynamics primarily by reducing new branch emergence and leads to impoverished terminal arbors. The results demonstrate a requirement for LPS in building arbor complexity and suggest a key role for pre-synaptic LPS in assembling neural circuits.

INTRODUCTION

CNS axons typically form highly branched terminal arbors in their synaptic target area. The branching complexity of an arbor defines the number and extent of post-synaptic partners a neuron can have and is a critical determinant of neural circuit assembly (Alsina et al., 2001; Meyer and Smith, 2006; Ruthazer et al., 2006). Previous studies have shown that retinal axon arbors are built in vivo through a highly dynamic process of branch extension, retraction, and stabilization (O'Rourke et al., 1994; Witte et al., 1996). Arbor size and dynamics are influenced by extrinsic stimuli, such as brain-derived neurotrophic factor (BDNF) and Netrin-1 (Cohen-Cory and Fraser, 1995; Manitt et al., 2009), and intrinsic factors, such as RNA-binding proteins (RBPs) (Hörnberg et al., 2013; Kalous et al., 2014). Branching is fundamental to functioning neural circuits, yet, although well described in dendrites (Dong et al., 2015), relatively little is known about the molecular mechanisms underlying axonal terminal branching in vivo.

Many guidance cues that trigger local protein synthesis (LPS) in axons, such as Netrin-1, BDNF, Sema3A, and Slit2 (Campbell and Holt, 2001; Piper et al., 2006), are also axon branch regulators (Kalil and Dent, 2014), suggesting a link between LPS and axonal branching. Indeed, recent evidence shows that knockdown of specific RBPs—Vg1RBP and Hermes—reduces retinal axon terminal arborization in *Xenopus* (Hörnberg et al., 2013; Kalous et al., 2014). Conversely, downregulation of the RBP fragile X mental retardation protein (FMRP), a negative translation regulator, increases axonal branching in *Drosophila* (Pan et al., 2004) and zebrafish (Tucker et al., 2006) neurons, indicating that precise RBP-regulated mRNA translation is required for appropriate branching. These studies disrupted gene function across all neuronal compartments (soma, dendrites, and axons), however, leaving open the question of whether axonally localized LPS has a role in branching.

Culture studies have uncovered an association between axonal branching and LPS. For example, newly synthesized green fluorescent protein (GFP) puncta localize to the base of collateral spikes in cultured retinal axons (Brittis et al., 2002), and the translational machinery localizes to branch points in cultured dorsal root ganglion (DRG) neurons (Spillane et al., 2012). Moreover, axonally synthesized regulators of the actin-nucleating Arp2/3 complex are involved in nerve growth factor (NGF)-induced collateral branching (Spillane et al., 2012) and targeting β-actin mRNA to axons supports collateral branching in an injury-conditioned paradigm (Donnelly et al., 2013). Consistent with these findings, mRNAs encoding proteins associated with branching are actively translated in arborizing mouse retinal ganglion cell (RGC) axon terminals in vivo (Shigeoka et al., 2016). These findings suggest that LPS may provide a critical link between extrinsic (branch-regulating) signals and branching, but the precise spatiotemporal dynamics of mRNA and LPS and their roles in axonal branching in vivo have not been examined.

In this study, we investigated the spatiotemporal dynamics of RNA movements, LPS, and axon terminal arborization in vivo. We developed a method to visualize *endogenous* RNA granules for prolonged periods (>1 hr) in single axons in the *Xenopus*

 © 2017 The Authors. Published by Elsevier Inc.
This is an open access article under the CC BY license (http://creativecommons.org/licenses/by/4.0/).

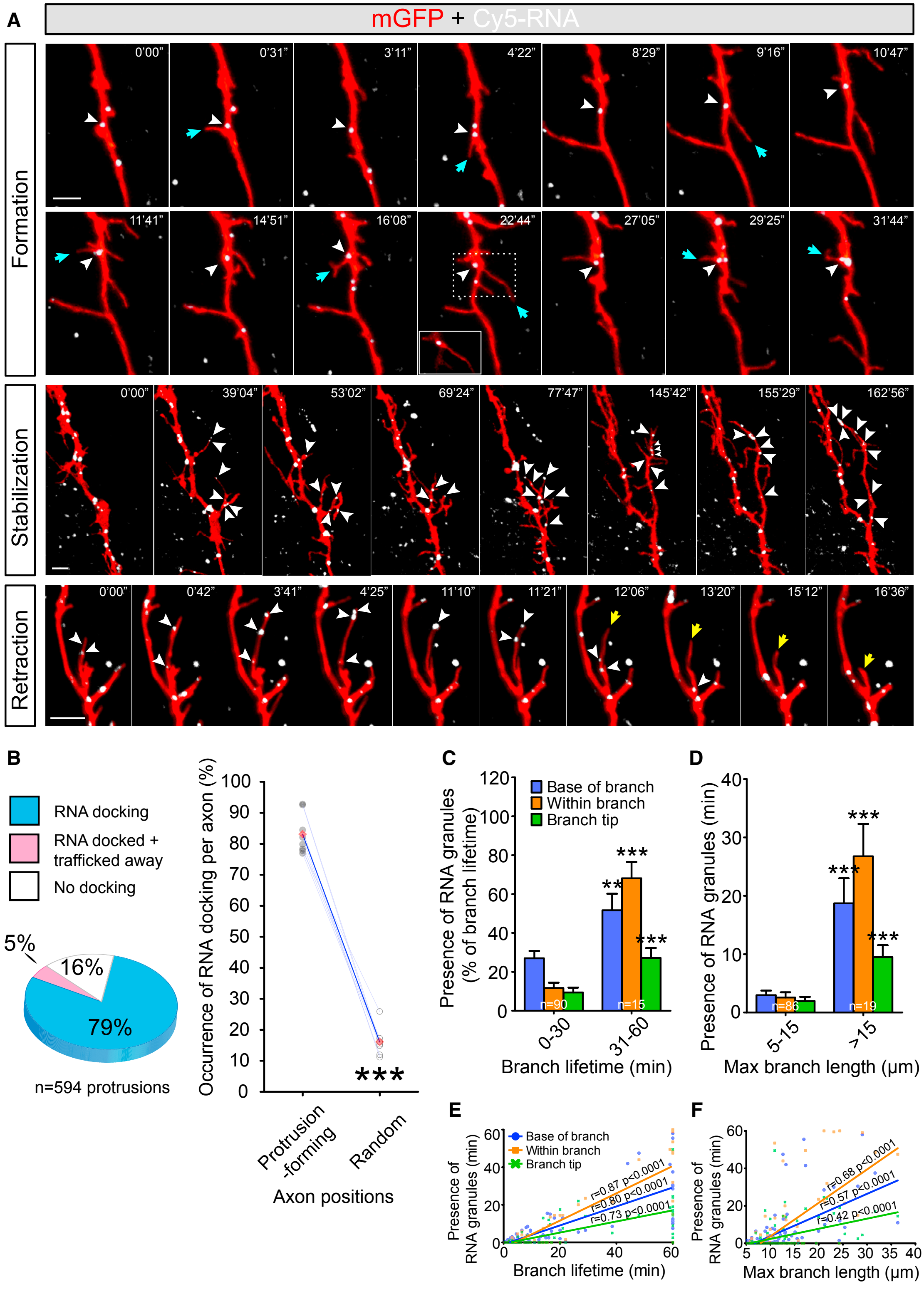

Figure 1. Dynamics of Endogenous RNA Granules Correlate with Distinct Aspects of Axon Branching In Vivo

(A) RNA granule (white arrowheads) docking in RGC axons during branching. Top: a single RNA granule docks at multiple branch point sites before the formation of protrusions (cyan arrows). The single z plane inset demonstrates localization of the RNA granule at the base of the protrusion. Middle: multiple RNA granules move into branches and to branch tips during protrusion stabilization. Bottom: branch retraction (yellow arrow) occurs shortly after RNA granules exit the branch.

(legend continued on next page)

visual system and performed live imaging to simultaneously capture arbor dynamics and RNA trafficking in vivo. Our results reveal a close relationship between arbor dynamics and RNA trafficking and show that RNA docking predicts sites of branch emergence. Live visualization of β-actin synthesis reveals the rapid accumulation of nascent β-actin in discrete "hotspots" in branches and at branch points. Functional experiments show that LPS is required for proper axon arbor formation in vivo. Knockdown of local β-actin synthesis causes a marked reduction in the emergence of new branches and results in impoverished axon terminal arbors. Collectively, the findings provide evidence of a pivotal role of LPS in determining axon arbor architecture in vivo.

RESULTS

Labeling Endogenous RNA for Live Imaging in Axons In Vivo

To label endogenous RNA, we delivered labeled uridine-5'-triphosphate (UTP) analogs, Cy5-UTP or biotin-UTP, intracellularly by eye electroporation or blastomere injection in *Xenopus* embryos. UTP analogs become incorporated into RNA during its synthesis and can then be monitored by live fluorescence imaging in putative ribonucleoproteins (RNPs) in retinal axons in vitro (Piper et al., 2015). The UTP analog was confirmed to be incorporated exclusively into RNA (including mRNA and rRNA) using qRT-PCR and bioanalyzer analysis of streptavidin/biotin-UTP pull-down following biotin-UTP blastomere injection and was not detected in genomic or mitochondrial DNA (Figures S1A–S1K). The method therefore provides an unbiased approach to label and track endogenous RNAs.

Next, we examined the general characteristics of endogenous RNA motility in RGC axon terminals in the tectum in vivo. Cy5-UTP was delivered into developing RGCs along with a membrane-targeted GFP (mGFP) reporter by targeted eye electroporation at stage 28, the beginning of RGC axonogenesis. Cy5-RNA and GFP-labeled axons were imaged in the optic tectum with time-lapse microscopy (10–20 frames/min for >1 hr) during the early phase of arborization and map formation (stages 41–43). Cy5-RNA appeared as punctate granules, often highly mobile, indicative of RNPs. We refer to these as "RNA granules." Motile RNA granules were observed in the majority of GFP-labeled retinal axon terminals with an average density of 2.6 ± 0.29 granules/10 μm (Movies S1 and S2). During an average 1 min period, 59.2% of the RNA granules were mobile and moved in anterograde (25.6% ± 3.4%) or retrograde (27.7% ± 3.4%) directions or bi-directionally (5.9% ± 2.0%), while 40.8% ± 3.9% remained stationary (Figure S1L). An analysis of granule speed along the main axon shaft (excluding branches) showed an average anterograde speed of 10.0 ± 0.7 μm/min and retrograde speed of 11.4 ± 1.3 μm/min (Figures S1M and S1N).

RNA Granules Dock at Sites of New Branch Emergence

We next investigated whether distinct aspects of branching, such as branch emergence and stabilization, are associated with the spatial and temporal positioning of RNA granules. New "branches" first appear as filopodial protrusions of less than 5 μm in length that emerge from the main axon shaft. Many of these are short lived, but some elongate to >5 μm and persist. We refer to these longer structures as "branches" to distinguish them from the shorter filopodial protrusions. Motile RNA granules were often observed to pause briefly (>10 s), or "dock," at sites of branch emergence in the axon shaft immediately preceding the appearance of a new protrusion. 84% of the filopodial protrusions exhibited docked RNA granules at their bases in the 10 s time window preceding their emergence. The docked RNA granules often persisted at the bases of filopodia during emergence, although in some cases (5%), the dwell time was transient (<10 s), and the granules moved away before the filopodia became visible (Figure 1B). Individual RNA granules occasionally exhibited repetitive docking at different sites along an axon where each dock site preceded the emergence of a new protrusion (Figure 1A; Movie S1), suggesting that specific RNA granules may be highly potent in their ability to initiate filopodial protrusions. Interestingly, while we rarely observed filopodial emergence, retraction, and re-emergence at the same sites, we often saw repeated cycles of partial retraction and extension (Movie S2) and instances of multiple filopodia emanating in different directions in 3D from the site of some docked RNA granules (green arrowheads from ~10 min onward in Movie S1).

To estimate what fraction of docking events lead to protrusion events, we generated 20 sets of time points and axon positions randomly for each axon (n = 5). The closest RNA granule to the randomized position was tracked across time to find the first instance of docking >10 s, and we then scored whether a protrusion emerges at this position within the 10 s of docking. We found that 22.0% ± 5.1% of RNA granules displaying this docking motion were followed by protrusion emergence. To test the possibility that these correlations are meaningful and not simply coincidental, we compared how frequently RNA granules docked at random positions along the axons versus an equivalent number of *bone fide* protrusion sites. Even though some of the random positions fell indiscriminately on protrusion-forming sites, we found that RNA granules docked at just 16% of random sites, compared to 84% at protrusion sites. (Figure 1B), showing that the correlation between the site of branch initiation and RNA granule docking is not simply coincidental. Moreover, the

(B) Left: proportion of protrusions with RNA docking at the base for >10 s preceding protrusion formation. Right: occurrence of RNA docking in protrusion-forming or random positions in the same axons ($t_7 = 21.2$, $p < 0.0001$, paired t test). Red diamonds represent the averages.

(C) Time of RNA granules presence was longer in branches with longer lifetime (base of branch: U = 369, p = 0.004; within branch: U = 137.5, p < 0.0001; branch tip: U = 225, p < 0.0001).

(D) Time of RNA granules presence was longer in branches with longer maximal branch length (base of branch: U = 240, p < 0.0001; within branch: U = 297, p < 0.0001; branch tip: U = 369, p < 0.0001). Error bars represent SEM. **p < 0.01, ***p < 0.001 (Mann-Whitney test for C and D).

(E and F) Pearson's correlation between time of RNA presence and lifetime of branch (E) or maximal branch length (F).

Scale bars, 5 μm. See also Figures S1–S3.

temporal order in which these events occur (RNA docking followed by branch emergence) is consistent with a causal role of RNA granule docking in protrusion initiation.

RNA Dynamics Correlate Positively with Branch Stabilization

Previous work has shown that only a small subset of newly formed branches persist to form stabilized mature branches, while the majority are short lived and retract within 10 min (Witte et al., 1996). Therefore, we next asked whether branches with lifetimes > 30 min, which we define as ‘‘persistent,’’ exhibited any distinct RNA granule behavior. RNA granules were seen to invade persistent branches and often localized to their distal tips (>15 μm; Figure 1A; Movie S2). By contrast, RNA granules were rarely observed making excursions into short-lived (<30 min) branches. Notably, RNA granules inside persistent branches often docked at sites from which a new protrusion or secondary branch emerged (arrowheads in Movie S2). The association between branch persistence and RNA invasion suggests a link between the two.

Occasionally, branches harboring RNA granules were seen to retract abruptly. Retrospective image analysis of these events showed, strikingly, that RNA granules were rapidly trafficked retrogradely out of the branch preceding retraction (yellow arrow in Figure 1A). Thus, branch formation, stabilization, and retraction appear to be closely coupled to the localization of RNA granules (Figure 1A; Movie S3).

To evaluate whether RNA localization correlates with branch lifetime and branch length, we categorized the branches into two groups on the basis of their lifetime—short lived (0–30 min) and persistent (31–60 min)—and length. The total time of the presence/absence of RNA granules at each of the three branch locations (base, tip, and intervening mid-region) was scored. RNA granules were present for significantly longer times in persistent branches compared with short-lived branches (Figure 1C). RNA granules were also present more of the time in longer branches (>15 μm) than in shorter branches (5–15 μm) (Figure 1D). In accordance with these results, we found that branch lifetime positively correlated with the duration of RNA localization at different branch positions (Figure 1E). In addition, the maximal branch length also correlated with the duration of RNA presence (Figure 1F). Thus, the spatial and temporal dynamics of RNA granules correlate with branch lifetime and length, consistent with the observation of increased RNA localization in stabilized branches.

Mitochondria Localize to Branch Points and Exhibit Parallel Behavior to RNA Granules

Mitochondria supply the energy for organelle trafficking and mRNA translation and have previously been shown to localize to branch points in axons in vitro (Courchet et al., 2013; Spillane et al., 2013). To visualize mitochondrial dynamics in axons in vivo, we introduced mitochondria-targeted GFP (mito-GFP) cDNA into RGCs by electroporation and conducted time-lapse imaging on axon arbors at stages 41–43. Mitochondria were commonly observed to accumulate at sites of axonal branch emergence (Figures S2A and S2B). They rarely entered transient branches but often moved into stabilized persistent branches (Figures S2A and S2C–S2F). Inside the branch, they commonly remained motile moving back and forth along the length of the branch and stalled at sites of new (secondary) branch formation. Like RNA granules, mitochondria moved out of branches immediately preceding retraction (Figure S2A). Dual imaging confirmed that Cy5-UTP and mito-GFP label distinct structures (Figure S3; Movie S4) and revealed that motile RNA granules frequently visited and stopped on mitochondria, remaining juxtaposed for significant periods (>3 min), and exhibited synchronous movements indicative of close interactions. Overall, our data indicate that endogenous RNA and mitochondria dock at sites associated with axonal branching in vivo, and their dynamics suggest a coupling of energy supply to RNA regulation.

Translation Inhibition Disrupts Axonal Branching Dynamics In Vivo

RNA transport and localization is intimately linked to LPS in neuronal compartments (Aakalu et al., 2001; Cosker et al., 2016; Kim et al., 2013; Leung et al., 2006; Tatavarty et al., 2012; Wu et al., 2016). To test the functional role of LPS in axon branching in vivo, we first used a pharmacological approach on the exposed brain preparation. The intact larval brain was exposed by simple removal of the overlying skin epidermis and protein synthesis (PS) inhibitors (cycloheximide/CHX and anisomycin/ANI) were added to the medium. Following electroporation of fluorescent reporters in the eye optimized for single RGC labeling, the in vivo arborization dynamics of RGC axons were imaged in the optic tract and/or the optic tectum every 30 s over a period of 10 min (stages 41–43; Figure 2A). To confirm that the treatment effectively inhibited PS in live brains, we developed a puromycylation-based translation assay on whole-mount brains to obtain a quantitative measure of PS. Exposed brains were treated for 30 min with PS inhibitors, followed by puromycin (puro) treatment for 10 min and subsequent anti-puro immunocytochemistry in whole-mount brains. Puro mimics tRNA and, at the low concentration used, is incorporated into the C termini of polypeptide chains, releasing them from ribosomes. PS inhibitor treatment greatly reduced the puro-immuno signal, confirming the effectiveness of the PS inhibition (Figures 3B and 3C).

In the control condition, single axon arbors were highly dynamic, with an average of 50 filopodia and 8 branches added and removed in 10 min (Figure 2B; Movie S5). By contrast, in brains treated with PS inhibitors, the arbor dynamics were reduced by 40%–70% (Figures 2C–2F; Movie S5). A detailed analysis revealed that the balance of addition and retraction of protrusions was also affected. In the control condition, slightly more filopodia (52 versus 47) and branches (7.8 versus 5.3) were added than were retracted (Figure 2B). This bias leads to a small, but consistent, net increase in the number of protrusions. Interestingly, upon acute inhibition of PS, the number of protrusions being added or removed became indistinguishable, tipping the normal balance from a net increase to an equilibrium (Figures 2C and 2D). The observed deficits were not due to PS inhibition in the RGC soma as we removed the eye prior to drug treatment. Previously, it was shown that somaless RGC axons continue to navigate to the tectum and arborize in a grossly normal manner in vivo for up to 3 hr (Harris et al.,

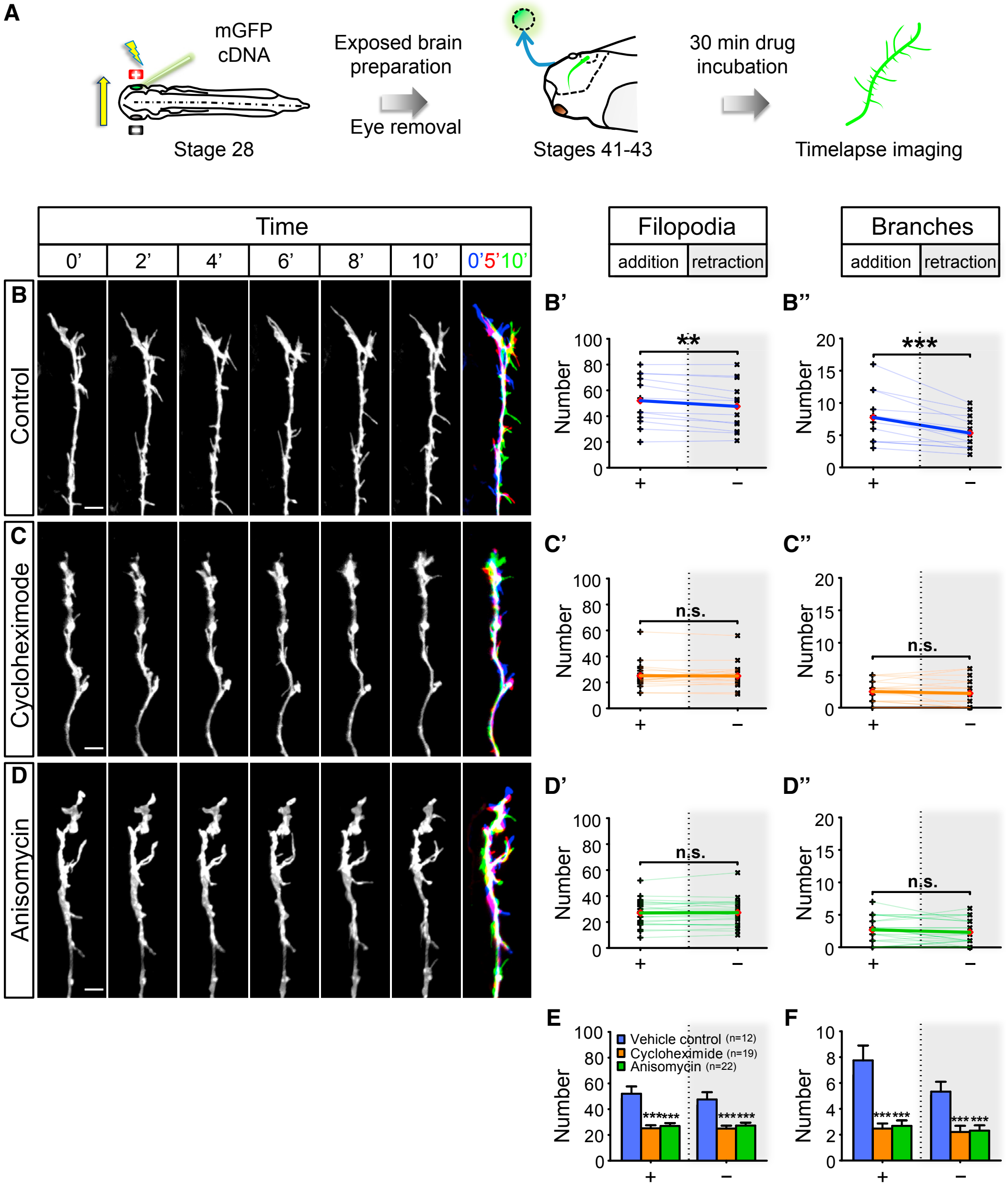

Figure 2. Acute Inhibition of Translation Disrupts Axonal Branching Dynamics In Vivo

(A) Live imaging experiment on branching dynamics of somaless RGC axons in the tectum in vivo. Electroporated eye was removed to eliminate somatic contribution.

(B–D) Axonal branching in control condition (B) and after incubation in translation inhibitors cycloheximide (C; CHX) and anisomycin (D; ANI). A merged overlay of three time points (0′, 5′, and 10′ in blue, red, and green, respectively) is shown for each condition (far right). More protrusions were added than removed in control condition (filopodia: $t_{11} = 3.8$, $p = 0.003$; branches: $t_{11} = 4.6$, $p = 0.0008$) (B′ and B″). No significant differences were observed in the number of protrusions that were added and removed in CHX condition (filopodia: $t_{18} = 0.2$, $p = 0.82$; branches: $t_{18} = 1.1$, $p = 0.29$) (C′ and C″). No significant differences were observed in the number of protrusions that were added and removed in ANI condition (filopodia: $t_{21} = 0.5$, $p = 0.66$; branches: $t_{21} = 1.4$, $p = 0.18$) (D′ and D″).

(E and F) The dynamics of filopodia (E; addition: $F_{2,50} = 18.7$, $p < 0.0001$; removal: $F_{2,50} = 13.0$, $p < 0.0001$) and branches (F; addition: $F_{2,50} = 20.2$, $p < 0.0001$; removal: $F_{2,50} = 9.5$, $p = 0.0003$) were inhibited by CHX or ANI treatment.

Error bars represent SEM. **$p < 0.01$, ***$p < 0.001$ (paired t test for B–D) versus Control ***$p < 0.001$ (one-way ANOVA with Tukey multiple comparisons test for E and F). Red diamonds represent the averages (B–D). Scale bars, 5 μm. See also Figure S4.

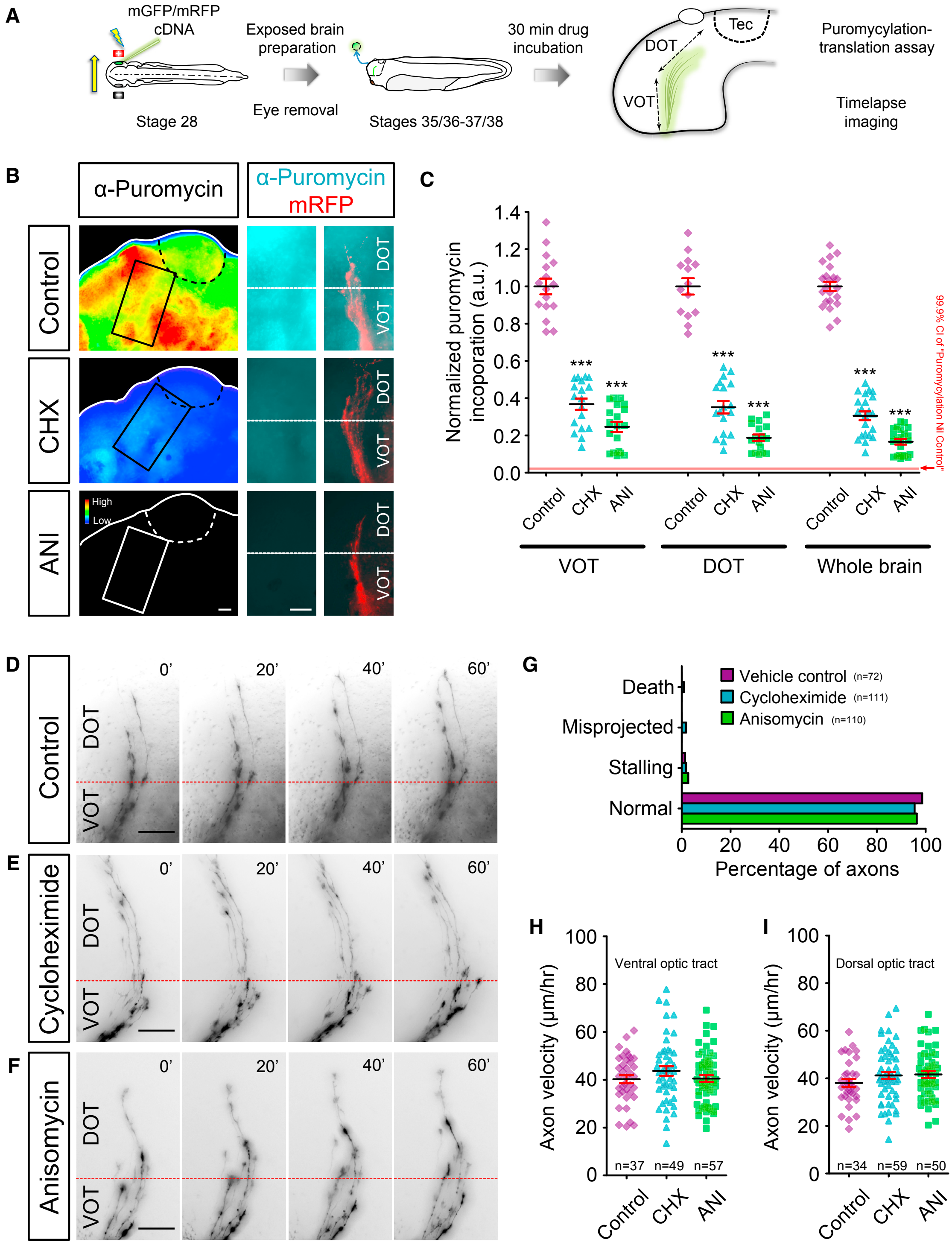

Figure 3. Axon Navigation in the Optic Tract Is Not Affected by Acute Inhibition of Translation

(A) Live imaging experiment on somaless RGC axon navigation in the optic tract in vivo and translation assay on whole brains. Electroporated eye was removed to eliminate somatic contribution.

(B) Anti-puromycin immunolabeling of whole-mount brains, shown as fluorescent intensity heatmaps, illustrates the incorporation of puromycin after 10 min, as readout of translation. Cycloheximide (CHX) and anisomycin (ANI) treatments greatly reduce puromycin immunolabeling.

(C) The incorporation of puromycin was reduced in the ventral optic tract (VOT) ($F_{3,67} = 204.6$, $p < 0.0001$), dorsal optic tract (DOT) ($F_{3,61} = 213.4$, $p < 0.0001$), and whole brain ($F_{3,80} = 501.9$, $p < 0.0001$) after CHX and ANI treatments.

(legend continued on next page)

1987). We extend these findings to show that within the time window of our experimental protocols (<1 hr), eye removal does not affect branching dynamics (Figure S4). These results are consistent with a possible role for LPS in axonal branching in vivo.

Embryos with eyes removed and treated with PS inhibitors for 30 min during the period of axon elongation in the optic tract (stages 35/36–37/38) did not exhibit abnormal pathfinding or stalling (Figure 3). Moreover, the speed of axon advance did not differ from control brains in either the ventral or the dorsal optic tract (Figures 3H and 3I). These findings indicate that acute PS inhibition does not cause gross defects in axon growth or navigation in the optic tract, whereas axonal branching is particularly sensitive to such treatment.

Knockdown of β-actin Synthesis Reduces Axon Arbor Complexity

β-actin mRNA localizes to axons (Bassell et al., 1998) and is locally translated in vitro in response to BDNF and Netrin-1 (Leung et al., 2006; Yao et al., 2006). These two cues are expressed in the optic tectum, and both act as key branch regulators of retinal axons in the tectum in vivo (Cohen-Cory and Fraser, 1995; Manitt et al., 2009). Furthermore, targeting of β-actin mRNA to injury-conditioned axons promotes branch formation in vitro (Donnelly et al., 2013), and the local remodeling of the actin network has been shown to regulate axonal branching in vivo (Chia et al., 2014). We thus focused on β-actin mRNA to further examine the role of LPS in the dynamics of axonal branching in vivo.

To block β-actin mRNA translation, a β-actin antisense morpholino (MO) was injected into the blastomeres fated to give rise to the CNS at the four-cell stage. This resulted in a 47% reduction in β-actin levels, in agreement with previous studies (Leung et al., 2006), and did not cause gross changes in embryogenesis (Figures S5A–S5E).

To target the β-actin knockdown to RGCs and to visualize the trajectories of single axons, we electroporated the β-actin MO together with a reporter mGFP plasmid into the eye at stage 28. At stage 45, when RGC axon arbors have reached maturity and become relatively stable, we imaged single arbors in the tectum and performed quantitative analysis. While highly complex arbors were seen in the control MO (Con MO) embryos, β-actin MO-axons exhibited much simpler arbors (Figure 4A). Quantitative branching analysis showed that the branch numbers decreased across different branch orders, leading to an overall drop of 56% (Figure 4B) and a 50% reduction of the total branch length (Figure 4C). The distribution of branches shifted toward lower branch orders in the β-actin MO condition compared to the Con MO condition, indicating that β-actin-depleted axons elaborate proportionally fewer high-order branches (Figure 4D). The axon complexity index (ACI) was used to assess the complexity of individual arbors (Figures 4E–4G), and arbors were classified as simple (ACI < 1.4) or complex (ACI ≥ 1.4). The results showed an average ACI of 1.83 for the Con MO condition that dropped dramatically to 1.38 in the β-actin MO condition. The majority of arbors (86%) in Con MO samples were in the complex category, compared to only 30% of those in the β-actin MO condition. Together, the data demonstrate that β-actin synthesis in RGCs is important for the elaboration of complex axon arbors in vivo.

β-actin Synthesis Promotes New Branch Emergence and Shifts Addition/Retraction Bias

The loss of arbor complexity could arise by a reduction in the emergence of new branches, a failure to stabilize new branches, or a combination of both. To understand the dynamic processes underlying axonal branching upon β-actin knockdown, we carried out live in vivo imaging following MO electroporation into the eye at stage 28 (Figure 4M). At stages 41–43, the dynamics of both filopodia and branches were significantly reduced after knocking down β-actin translation (Figures 4H and 4I; Movie S6). In the Con MO axons, there were, on average, 41 filopodia added and 35 retracted, while 21 filopodia were added and retracted in the β-actin MO axons (Figure 4K). For the branches, an average of 6.7 was added and 4.7 retracted in the control, compared to 1.3 and 1.5 for addition and retraction, respectively, in the β-actin MO condition (Figure 4L). Analogous to the trend observed with acute translation inhibition (Figure 2), the net increases in the control for both filopodia and branches were abolished in the β-actin knockdown (Figures 4H and 4I). To test the specificity of the β-actin MO, we electroporated an MO-insensitive β-actin construct into the eye along with the MO. The MO-insensitive β-actin was co-expressed with mGFP in a dual promoter construct to ensure that all observed GFP-positive axons also expressed the rescue construct. This rescued the branching deficits (Figures 4A–4M). Collectively, these results indicate that de novo synthesis of β-actin regulates axon branching dynamics in RGCs by promoting (1) the emergence of new branches and (2) a small bias in favor of branch stabilization over retraction.

Local β-actin mRNA Translation Is Autonomously Required in Axon Terminals for Branching

The above results point to a requirement for de novo β-actin synthesis for arborization but do not address whether the synthesis is localized to the axon because the translation-blocking MO was delivered into the cell somas in the eye. To confirm a local (axonal) effect, we delivered the MO directly into arborizing RGC axons in the tectum by electroporation at stages 41–43 (Figure 5E) and conducted live imaging immediately thereafter. In the Con MO condition, the arbors were dynamic with highly motile filopodia and branches, whereas the motility was severely reduced in the β-actin MO condition (Figures 5A–5D; Movie S7). In addition, we also recorded the expected biases in dynamics that resulted in net increases for both filopodia and branches

(D–F) Axon navigation through the VOT and DOT in control (D) and after incubation in translation inhibitors CHX (E) and ANI (F).

(G) Axon behaviors were unaffected in axons after CHX or ANI incubation (death: $p = 0.44$; misprojected: $p = 0.19$; stalling: $p = 0.80$; normal: $p = 0.47$, chi-square test).

(H and I) Axon elongation velocities were unaffected by CHX or ANI incubation (H, VOT: $F_{2,140} = 1.3$, $p = 0.29$; I, DOT: $F_{2,140} = 1.3$, $p = 0.27$).

Error bars represent SEM versus Control ***$p < 0.001$ (one-way ANOVA with Tukey multiple comparison's test for C, H, and I). Scale bars, 50 μm.

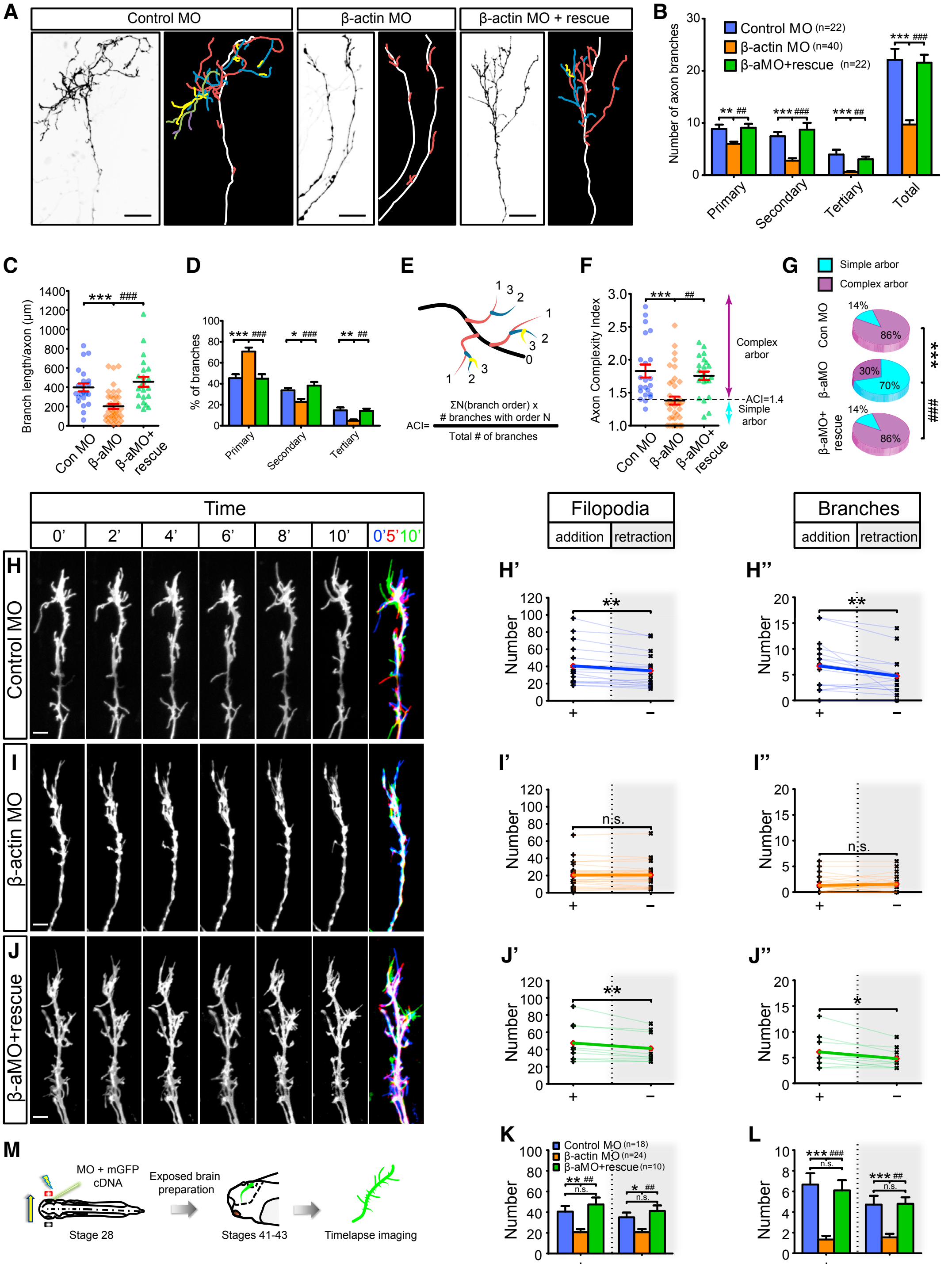

Figure 4. Knockdown of β-actin Reduces Axon Branching Dynamics and Arbor Complexity In Vivo

(A) Lateral view of single RGC axons in the tectum. Line drawings are shown with the branch order color coded: white, axon shaft; branches: red, primary; blue, secondary; yellow, tertiary; purple, quaternary.

(B) Reduction in number of branches in β-actin morphants (primary: $F_{2,81} = 8.9$, $p = 0.0003$; secondary: $F_{2,81} = 17.6$, $p < 0.0001$; tertiary: $F_{2,81} = 13.0$, $p < 0.0001$; total: $F_{2,81} = 29.3$, $p < 0.0001$).

(C) Branch length decreased in the β-actin MO (β-aMO) condition ($F_{2,81} = 14.69$, $p < 0.0001$).

(D) The proportion of branches in the β-aMO condition shifts toward lower branch orders (primary: $F_{2,81} = 2.1$, $p < 0.0001$; secondary: $F_{2,81} = 4.7$, $p = 0.0006$; tertiary: $F_{2,81} = 4.2$, $p = 0.0002$).

(legend continued on next page)

in the Con MO condition (Figure 5A). Similar to the global inhibition of β-actin translation in RGCs (Figure 4I), the local inhibition of β-actin translation abolished the net increases (Figure 5B). The defects in branching dynamics were partially rescued by locally co-electroporating the β-actin MO with a MO-insensitive β-actin mRNA (Figure S4). Because the electroporation protocol delivers the β-actin MO into surrounding tectal cells, as well as the RGC axons, it is possible that the axon branching defects arose due to non-autonomous effects. To eliminate this, we delivered the MO into the tectum *before* the arrival of RGC axons (stages 35/36–37/38) and subsequently visualized the branching dynamics of axons after tectal entry at stages 41–43 (Figure 5J). We found no difference in the branching dynamics between the Con MO and β-actin MO conditions (Figures 5F–5I), indicating that β-actin translation in tectal cells is not required for RGC axonal branching, at least in the short term. Thus, the data demonstrate that local β-actin synthesis promotes axonal branching in RGC axons in vivo.

In contrast to the severe effect on arborization, the trajectories of β-actin MO axons in the optic tract did not exhibit any major guidance defects or abnormal extension rates (Figures S5 and S6; Movie S8), indicating that axon pathfinding is not sensitive to the level of β-actin translation knockdown achieved.

De Novo β-actin Synthesis Visualized by FRAP in Axon Terminals In Vivo

We next sought to visualize newly synthesized β-actin during branching in axon terminals in the tectum in vivo. Fluorescence recovery after photobleaching (FRAP) of GFP has been used in vitro to demonstrate the local synthesis of proteins (Aakalu et al., 2001; Job and Eberwine, 2001). We expressed a fast-folding fluorescent β-actin translation reporter, Venus-β-actin (Figure 6A), and mRFP (general cell marker) in RGCs via targeted eye electroporation and conducted in vivo FRAP on RGC axons in the tectum (Figure 6B). The eye was removed to prevent diffusion of soma-derived, Venus-tagged proteins into the axons. Axon terminals expressing the Venus control showed a minimal amount of signal recovery (4.8%) 10 min post-photobleaching. By contrast, Venus-β-actin expressing axons exhibited rapid fluorescence recovery reaching 18.6% in just 5 min. Cycloheximide suppressed the recovery of Venus-β-actin (Figures 6C and 6D; Movie S9). These data demonstrate that Venus-β-actin is rapidly and locally synthesized in axon terminals in the tectum.

Surprisingly, when the same 10 min FRAP experiment was carried out on Venus-β-actin-expressing growth cones in the optic tract, no signal recovery was observed (Figure S7). This result indicates that β-actin mRNA translation is relatively low in axon tips while navigating in the optic tract, consistent with our results showing that β-actin knockdown does not affect optic tract pathfinding. This contrasts with axon tips once they reach the optic tectum, where β-actin synthesis is significantly upregulated, consistent with a functional role in arborization.

Focal β-actin Translation Promotes the Formation of β-actin Microdomains in Axonal Branches In Vivo

De novo synthesis of β-actin could occur uniformly along the entire axon and/or branch providing a continuous supply of new actin monomers for branching axons. Alternatively, it could take place in focal hotspots, potentially providing new nucleation sites for actin polymerization. To investigate the spatial distribution of newly synthesized β-actin, we used FRAP with high-resolution time-lapse microscopy to map the subcellular location of newly synthesized β-actin in arborizing axons in vivo (Figure 7A).

Venus-β-actin showed a markedly different subcellular localization to the control Venus reporter. The control Venus reporter exhibited low intensity and fairly ubiquitous levels of signal recovery as expected for free diffusion, whereas the Venus-β-actin exhibited more concentrated spots of high intensity that we refer to as LPS hotspots (Figures 7B and 7C; Movie S10). The post-FRAP hotspots commonly appeared within 30 s and intensified over time, suggesting that the newly synthesized Venus-β-actin is retained focally at the site of translation, where it can potentially participate in nucleating F-actin polymerization. Moreover, consistent with the RNA granule docking behaviors observed during branching (Figure 1), these nascent protein hotspots were found at the branch points, within branches, and at branch tips (Figure 7C), which may reflect a role in elongating branches. A single branch can exhibit multiple hotspots, as shown in the kymograph (Figure 7C), where four distinct hotspots can be seen forming in different locations along the same branch with different kinetics. The remarkably rapid detection of the FRAP Venus-β-actin signal (10–20 s) was likely aided by the high sensitivity of the microscope custom built for single-molecule fluorescence and the exogenous nature of expression. The result indicates the existence of spatially and temporally distinct translation microdomains and suggests that locally synthesized β-actin may fuel different aspects of branch remodeling.

To perform an unbiased quantitative analysis of the hotspots, we measured the variation in the fluorescence signal, which is predicted to increase with the presence of hotspots. The standard deviation (SD) of fluorescence values was used to generate

(E) Formulation of axon complexity index (ACI).
(F) The ACI was reduced in the β-aMO condition ($F_{2,81} = 12.0$, $p < 0.0001$).
(G) The percentage of complex arbor (ACI ≥ 1.4) was reduced in β-aMO condition (***$p < 0.0001$, ###$p < 0.0001$, Fisher's exact test).
(H–J) Axon branching in Con MO- (H) and β-aMO-positive (I) (+/– rescue construct; J) axons in the tectum. More protrusions were added than removed in control morphants (filopodia: $t_{17} = 3.9$, $p = 0.0011$; branches: $t_{17} = 3.2$, $p = 0.0049$) (H′ and H″). No significant differences were observed in the number of protrusions that were added and removed in β-actin morphants (filopodia: $t_{23} = 0$, $p = 1$; branch: $t_{17} = 0.8$, $p = 0.42$) (I′ and I″). More protrusions were added than removed in β-actin morphants that were rescued with β-aMO resistant construct (filopodia: $t_9 = 3.5$, $p = 0.007$; branches: $t_9 = 2.8$, $p = 0.022$) (J′ and J″).
(K and L) The dynamics of filopodia (K; addition: $F_{2,49} = 9.3$, $p = 0.0004$; removal: $F_{2,49} = 6.6$, $p = 0.003$) and branches (L; addition: $F_{2,49} = 16.1$, $p < 0.0001$; removal: $F_{2,49} = 10.2$, $p = 0.0002$) were inhibited in β-actin morphants.
(M) Eye electroporation and live imaging of axonal branching.
Error bars represent SEM. *$p < 0.05$, **$p < 0.01$, ***$p < 0.001$, ##$p < 0.01$, ###$p < 0.001$ (one-way ANOVA with Tukey multiple comparisons test for B–F, K, and L) and paired t test for H–J). Red diamonds represent the averages (H–J). Scale bars, 20 μm for (A) and 5 μm for (H–J). See also Figure S5.

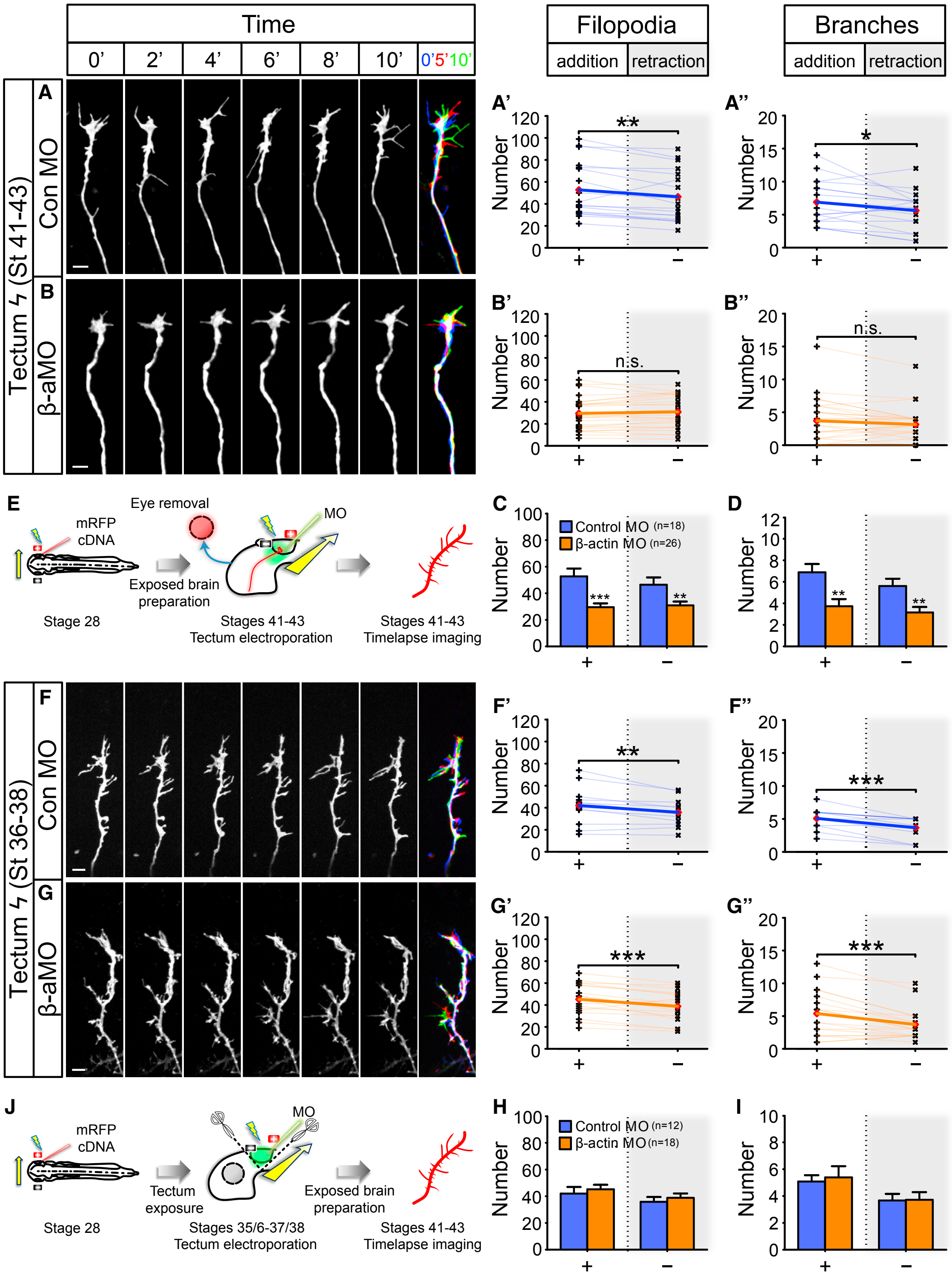

Figure 5. Local Synthesis of β-actin Is Required for Axon Branching In Vivo

(A and B) Axon branching in the tectum after local delivery of MO at stages 41–43. More protrusions were added than removed in Control MO (Con MO) condition (filopodia: $t_{17} = 3.1$, $p = 0.007$; branches: $t_{17} = 2.4$, $p = 0.03$) (A′ and A″). No significant differences were observed in the number of protrusions that were added and removed in β-actin MO (β-aMO) condition (addition: $t_{25} = 1.4$, $p = 0.16$; removal: $t_{25} = 1.9$, $p = 0.07$) (B′ and B″).

(C) Filopodia dynamics were inhibited in β-aMO condition (addition: $t_{42} = 3.9$, $p = 0.0004$; removal: $t_{42} = 2.7$, $p = 0.01$).

(D) Branch dynamics were inhibited in β-aMO condition (addition: $t_{42} = 3.1$, $p = 0.004$; removal: $t_{42} = 3.0$, $p = 0.005$).

(legend continued on next page)

a fluorescence variation index (FVI). The SD value of the fluorescence for the branches were internally normalized to the SD of the fluorescence for the axon shaft, where a ubiquitous recovery pattern would yield a FVI ≈ 1 and the presence of hotspots would result in a FVI > 1 (Figure 7D). In agreement with our qualitative analysis, Venus-β-actin presented an increasingly larger FVI value, whereas the FVI remained largely the same for the Venus control (Figure 7E). Pre-treatment with cycloheximide abolished the rise of FVI for Venus-β-actin, confirming that this is PS dependent. Taken together, our data support a model whereby focally translated β-actin is retained in concentrated spots, where it may promote the local assembly of F-actin required for axonal branching.

Nascent β-actin Microdomains Form in Close Proximity to Docked RNA Granules In Vivo

To discover whether β-actin is synthesized at docked RNA granules, we carried out high-resolution FRAP experiments and recorded the recovery of Venus-β-actin signal simultaneously with Cy5-RNA localization in branching axons in vivo. Hotspots of β-actin recovery were found highly associated with docked RNA granules, beginning 10–20 s post-FRAP (Figures 8A and 8B; Movie S11). To quantify this relationship, we compiled each Cy5-RNA time series into a z stack and computed the median signal intensities as a representation of RNA dwell time at different positions in the axon. The resulting image was then used to calculate Pearson's correlation coefficient (R) with the Venus-β-actin cumulative recovery signal. We obtained a high R of 0.76 ± 0.04 (Figure 8C), supporting a strong association. To test for the significance of the observed association, we scrambled each image to create 1,000 random images and yielded an average R of 0.10 ± 0.02. It is interesting to note that the Venus-β-actin hotspots frequently persisted for several minutes, suggesting that newly synthesized β-actin can be concentrated in microdomains.

DISCUSSION

We used live imaging to show that new axon branches commonly emerge at sites where RNA granules and mitochondria dock. We found that branch lifetime correlates positively with sustained RNA and mitochondrial invasion. Our functional analysis shows that PS is required for the elaboration of complex arbors and that local β-actin synthesis contributes to the addition and stabilization of new branches. Translation reporter experiments show that β-actin is focally synthesized and retained in concentrated microdomains at the bases and interiors of axonal branches. Our findings are consistent with a model in which RNA localization and nascent β-actin help to direct branch emergence and expand arbor complexity in response to target post-synaptic cell contact.

Intracellular delivery of fluorescent UTP labels endogenous RNAs and has enabled us to perform tracking of the intra-axonal movements of RNA granules. These granules are highly fluorescent and photostable and can be followed for prolonged periods (>420 min). Because the fluorescent UTP is incorporated into RNA during transcription in vivo, the RNAs may associate with normal regulatory proteins inside the nucleus to form specific RNP complexes before export to the cytoplasm. The dynamic behavior of these granules therefore should provide an accurate reflection of their endogenous activities. The MS2 genetic system is a valuable alternative for labeling endogenous RNAs for live imaging (Beach et al., 1999; Bertrand et al., 1998) and has the advantage of being able to label specific RNAs. However, the multiple MS2 binding stem-loops in the RNA and the binding of multiple fluorescent proteins required for live tracking may hamper RNA-protein interactions and alter some aspects of motility and localization. Our finding that RNA granules frequently dock at sites immediately preceding filopodia emergence suggests that translation may occur at these sites and is supported by dual time-lapse imaging, showing a strong association between the positions of newly synthesized Venus-β-actin and docked RNA granules. Since our biochemical analysis showed that rRNA, similar to non-labeled RNA, makes up a large fraction of labeled RNA in larval brains, it is likely that the docked RNA granules represent accumulations of ribosomes as well as mRNAs. It is notable that the RNA docking behavior described here is similar to that reported for Vg1RBP (the major β-actin mRNA-binding protein) (Kalous et al., 2014), where GFP-Vg1RBP puncta dock at sites immediately preceding the emergence of filopodia from the axon shafts. Collectively, these data suggest that numerous translation-associated organelles and complexes (RNA granules, ribosomes, mitochondria, RBPs) co-dock at sites to promote branch emergence. In the future, it will be interesting to investigate the dynamics of multiple complexes simultaneously.

Our fast image-capture analysis enabled us to simultaneously track RNA, translation, and branching events and revealed the highly dynamic nature of developing axon arbors with multiple branches emerging and retracting over minutes. This is in broad agreement with previous studies using slower capture rates (Alsina et al., 2001; Cohen-Cory and Fraser, 1995; Harris et al., 1987; Hu et al., 2005; Meyer and Smith, 2006; O'Rourke et al., 1994; Ruthazer et al., 2006; Witte et al., 1996). We found a slight

(E) Local delivery of MO into RGC axons by tectum electroporation and imaged immediately thereafter. Electroporated eye was removed to eliminate somatic contribution.

(F and G) Time-lapse images of axonal branching in the tectum at stages 41–43 after local delivery of MO at stages 35/36–37/38. More protrusions were added than removed in Con MO condition (filopodia: $t_{11} = 3.4$, $p = 0.006$; branches: $t_{11} = 4.9$, $p = 0.0005$) (F′ and F″). More protrusions were added than removed in β-aMO condition (addition: $t_{17} = 4.2$, $p = 0.0006$; removal: $t_{17} = 4.0$, $p = 0.0008$) (G′ and G″).

(H and I) The dynamics of filopodia (H; addition: $t_{28} = 0.6$, $p = 0.58$; removal: $t_{28} = 0.6$, $p = 0.55$) and branches (I; addition: $t_{28} = 0.3$, $p = 0.78$; removal: $t_{28} = 0.07$, $p = 0.95$) were unaffected in β-aMO condition.

(J) Local delivery of MO into the tectum before tectal entry of RGC axons (stages 35/36–37/38) and live imaging of axonal branching after tectal entry of RGC axons (stages 41–43). Scissors and dashed line denote that only the skin overlying the tectal area was removed to minimize damage to the brain.

Error bars represent SEM. *$p < 0.01$, **$p < 0.01$, ***$p < 0.001$ (paired t test for A, B, F, and G and unpaired t test for C, D, H, and I). Scale bars, 5 μm. Red diamonds represent the averages (A, B, F, and G). See also Figures S4 and S6.

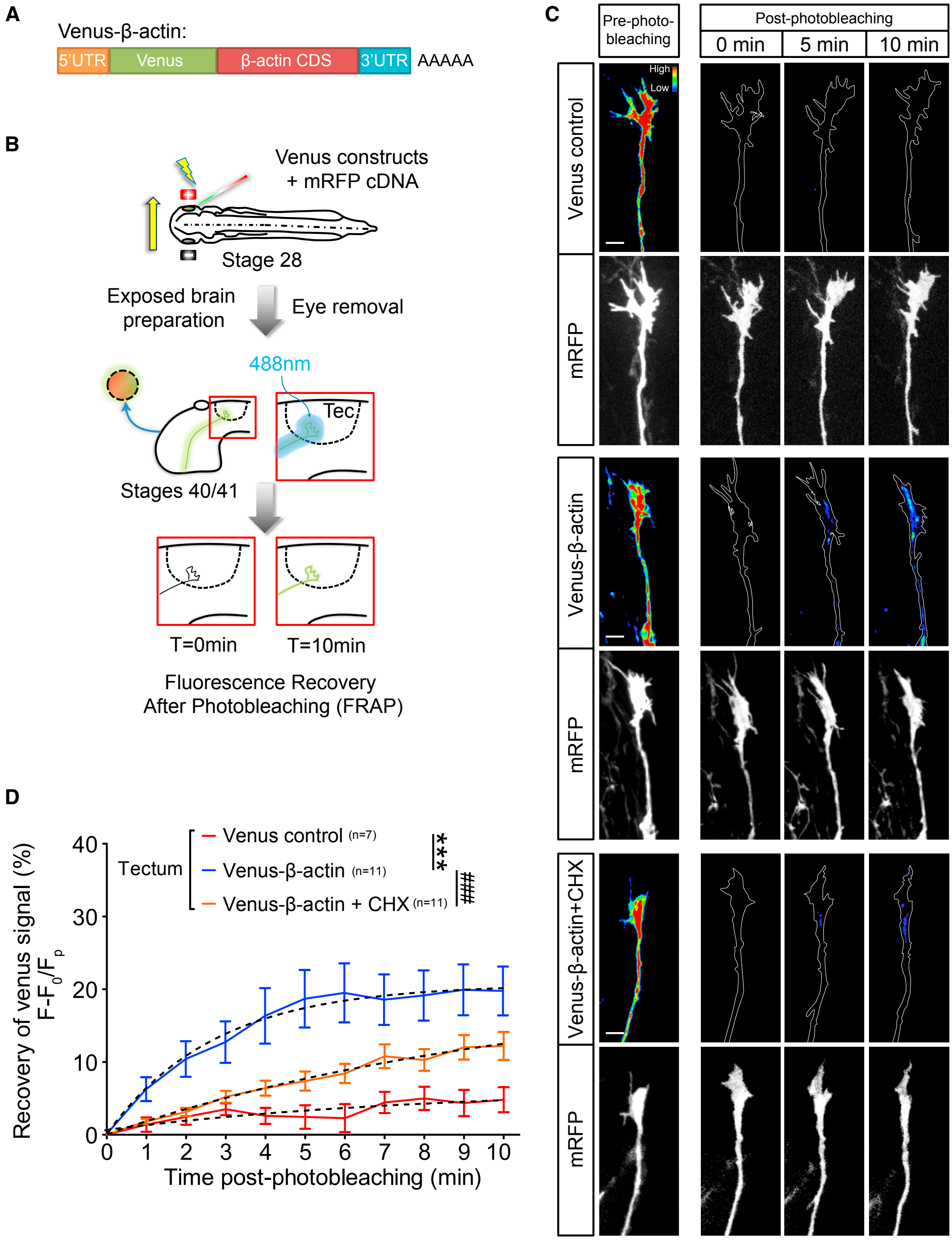

Figure 6. Visualization of De Novo β-actin Synthesis in Axon Terminals In Vivo

(A) Venus-β-actin construct as a reporter for β-actin translation.

(B) Fluorescence recovery after photobleaching experiment of Venus constructs in vivo. Electroporated eye was removed to eliminate somatic contribution. Tec, tectum.

(C) Fluorescence heatmaps illustrating that limited recovery was detected with the Venus control construct. In contrast, Venus-β-actin signal recovered soon after photobleaching and was inhibited by the translation inhibitor cycloheximide (CHX), indicating de novo synthesis of β-actin in RGC axon terminals in the tectum.

(D) FRAP over the course of 10 min. Dotted lines represent least-squares fits to a single-exponential function. (Venus control versus Venus-β-actin: $F_{3,191} = 36.0$, $p < 0.0001$; Venus-β-actin versus Venus-β-actin + CHX: $F_{3,236} = 21.8$, $p < 0.0001$; extra sum-of-squares F test). Error bars represent SEM. Scale bars, 5 μm. See also Figure S7.

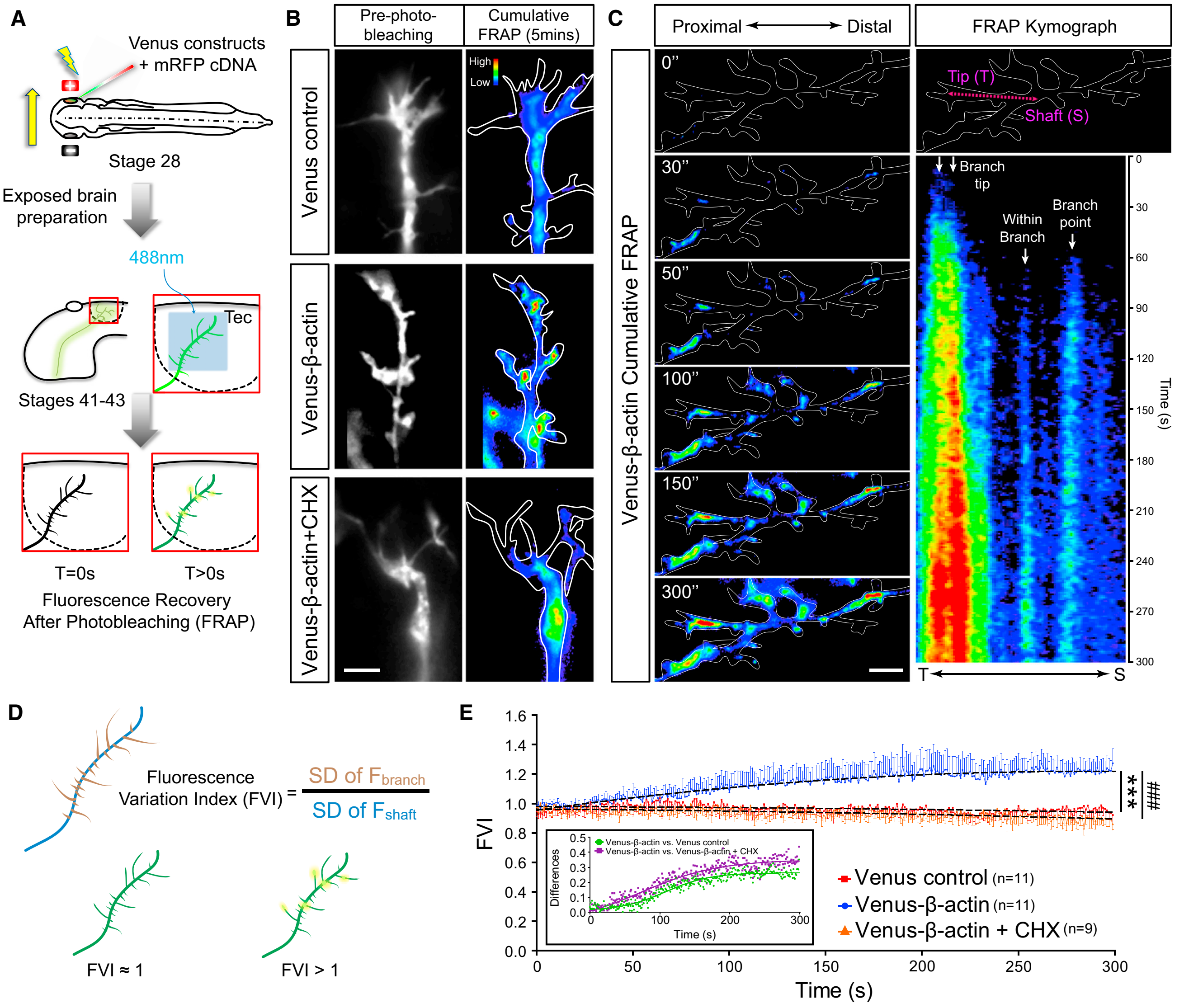

Figure 7. Focal Hotspots of Nascent β-actin at Axonal Branch Points and within Branches In Vivo

(A) Fluorescence recovery after photobleaching experiment of Venus constructs in vivo.

(B) Fluorescence heatmaps illustrating a ubiquitous recovery pattern for the Venus control construct. In contrast, Venus-β-actin signal recovered in hotspots at branch points and within branches. The formation of these hotspots was inhibited by cycloheximide (CHX), indicating de novo synthesis and accumulation of β-actin in highly specific focal points in RGC axons.

(C) An example of multiple Venus-β-actin hotspots forming at different sub-compartments of a branch. Kymograph displays the 300 s FRAP from the branch tip to axon shaft as indicated by the magenta arrow. At least four distinct hotspots can be identified in this single branch.

(D) The fluorescence variation index (FVI) is defined by normalizing the standard deviation (SD) of fluorescence in branches (F_{branch}) to the SD of fluorescence in axon shaft (F_{shaft}).

(E) FVI after FRAP over the course of 5 min. Dotted lines represent least-squares fits to a quadratic function. (Venus control versus Venus-β-actin: $F_{3,6594} = 396$, $p < 0.0001$; Venus-β-actin versus Venus-β-actin + CHX: $F_{3,5994} = 466.7$, $p < 0.0001$). Inset displays the differences between the conditions. Error bars represent SEM. ***$p < 0.001$, ###$p < 0.001$ (extra sum-of-squares F test for E). Scale bars, 5 μm.

bias in favor of emergence over retraction, which leads to a progressive increase in arbor size and complexity. New branches emerging from the axon shaft typically appear at sites of transiently docked RNA granules and are often short lived, retracting within seconds, with the more stable branches invaded by RNA. Interestingly, the RNA docking sites closely resemble locations of presynaptic puncta, which are similarly correlated with branch emergence and lifetime (Alsina et al., 2001; Hu et al., 2005; Meyer and Smith, 2006; Ruthazer et al., 2006). This suggests that translation and presynaptic components might coordinately control arbor remodeling and transmission. Many presynaptic proteins are axonally translated during arborization (Shigeoka et al., 2016), suggesting the possibility that the positioning of presynaptic proteins and puncta might be aided by RNA localization.

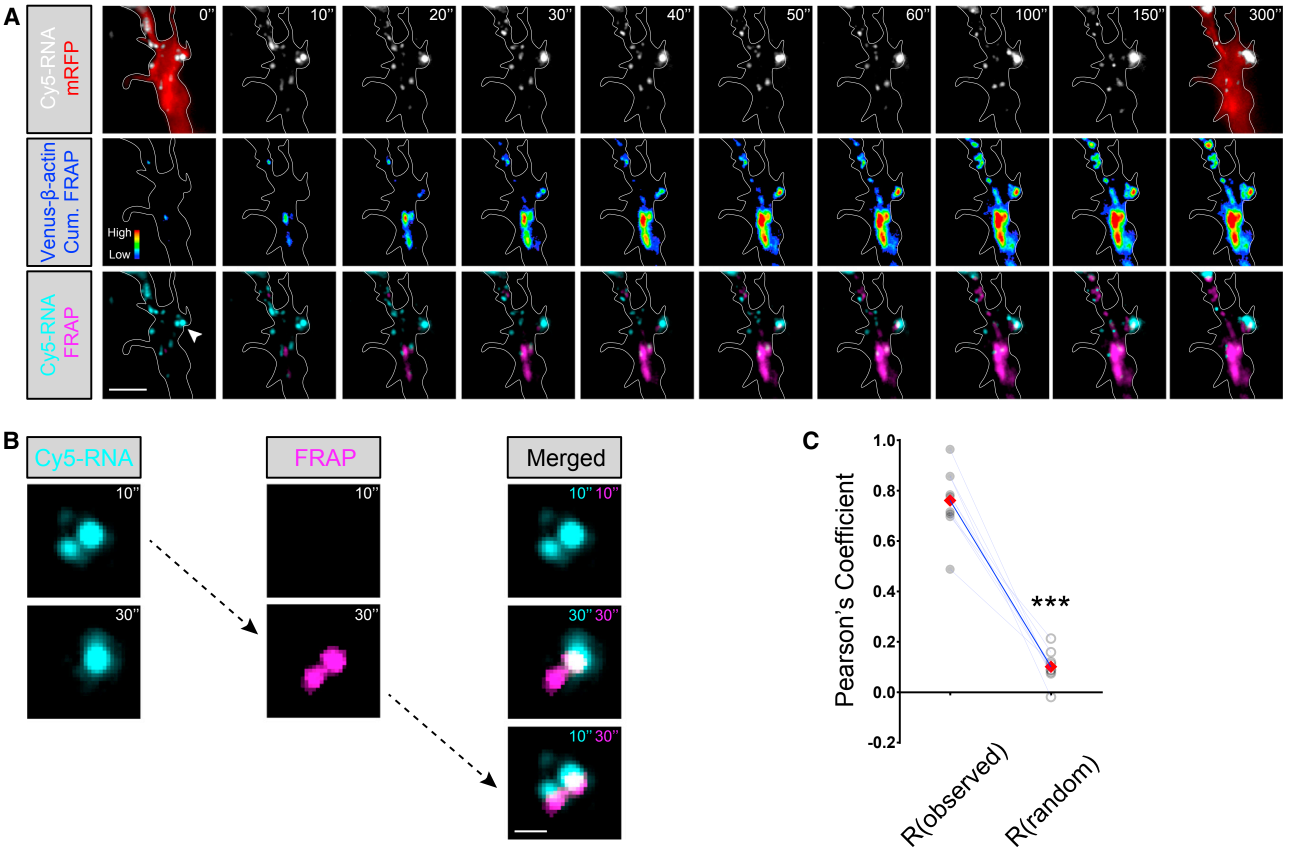

Figure 8. Nascent β-actin Microdomains Form in Close Proximity to Docked RNA Granules In Vivo

(A) Dual-channel simultaneous time-lapse images of Cy5-RNA and Venus-β-actin FRAP. The axon morphology was estimated by capturing a mRFP image before photobleaching and after time-lapse acquisition, which are overlaid on the Cy5-RNA images (gray scale, top) at time points 0″ and 300″, respectively. Axon outline of the mRFP image captured after time-lapse acquisition was used as an approximation for time points 10″ to 300″. Venus-β-actin signal recovery after photobleaching is illustrated by the fluorescence heatmaps (middle). The bottom row presents the overlays of Cy5-RNA (cyan) and Venus-β-actin FRAP (magenta).

(B) Enlarged images of area signified by the arrowhead in (A). The images of Cy5-RNA (cyan) and Venus-β-actin FRAP (magenta) are individually presented on the left and in the middle columns. Images on the right display image overlays. The FRAP signal positions at 30 s closely resemble the localization of RNA at 10 s.

(C) Cy5-RNA time series were compiled into z stacks and computed for the median signal intensities as a representation of RNA dwell time. The resulting images were then used to compute Pearson's correlation coefficient (R) with the Venus-β-actin cumulative recovery signal. The averages of R(observed) were significantly higher than averages of R(random) yielded from 1,000 random images scrambled from each original axon image ($t_8 = 11.55$, $p < 0.0001$, paired t test).

Scale bars, 5 μm for (A) and 1 μm for (B).

Previous work has shown that LPS is required for growth cone turning in vitro in response to various factors, including Netrin-1 and BDNF (Campbell and Holt, 2001; Yao et al., 2006). Interestingly, Netrin-1 and BDNF are expressed in the tectum, but not in the optic tract, and are known to induce β-actin synthesis in retinal axons (Leung et al., 2006). In addition, the Netrin-1 receptor DCC, being bound to ribosomes, is directly linked to the translation machinery (Tcherkezian et al., 2010). Thus, it can be envisaged that on arrival in the tectum, contact with Netrin-1-bearing tectal dendrites triggers localized β-actin translation (via DCC activation) in retinal axons, which promotes branch emergence.

Defects in axon pathfinding were not detected in the current study after β-actin translation knockdown. This is consistent with the finding that Vg1RBP knockdown does not cause pathfinding defects but disrupts axon arborization (Kalous et al., 2014). It is puzzling that β-actin translation knockdown affects growth cone turning in vitro, but not pathfinding in vivo. One possibility is that the growth-cone turning assay, which measures small shifts in the direction of axon growth over a 1 hr period, is not a faithful gauge of long-range axon pathfinding behavior in vivo. The limited behavioral responses possible in the reduced conditions of this in vitro assay system may, in fact, be more akin to an arborizing axon, although overt branching cannot occur because of the absence of appropriate cellular substrate and branch-inducing factors. The finding that RGC axonal growth cones can navigate accurately in optic tract despite reduced levels of β-actin mRNA translation is consistent with our observation of scant levels of new β-actin synthesis in the growth cones of navigating axons using a Venus-β-actin translation reporter. By contrast, the translation reporter revealed robust β-actin synthesis in the tips of axons that have entered the

tectum. Thus, our data indicate that growing retinal axons upregulate β-actin synthesis on entering the tectum, where it is required for branching. The results raise the interesting possibility that distinct aspects of axon development, such as axon pathfinding and branching, are differentially sensitive to local demands for de novo protein synthesis. Our results do not exclude the possibility that axon pathfinding requires some level of LPS. In commissural axons, although a requirement for LPS has not been demonstrated in pathfinding, a similar upregulation of EphA2 translation and Robo3.2 mRNA occurs after axons pass the midline of the spinal cord (Brittis et al., 2002; Colak et al., 2013). Moreover, >1,000 mRNAs were identified in the translatome of retinal axons elongating in the superior colliculus in embryonic day 17.5 (E17.5) mice (Shigeoka et al., 2016). LPS could aid short-range (within the target) pathfinding and promote the accuracy of connectivity. This is supported by the misrouting of retinal axons selectively in the optic tectum after the knockdown of specific microRNAs miR-124 (Baudet et al., 2011) or the axon-localizing miR-182 (Bellon et al., 2017), which modulate the translation of subsets of mRNA.

How might a small quantity of newly synthesized β-actin play a physiologically significant role in branch dynamics, promoting branch formation and stabilization, especially when there is a large pool of pre-existing actin? Locally synthesized β-actin has been estimated to constitute <1% of the actin in sympathetic neuron axons (Eng et al., 1999) and 7% of the actin needed for polymerization in migrating fibroblasts (Condeelis and Singer, 2005). Additionally, actin reportedly has a long half-life (2–3 days) (Antecol et al., 1986). In fibroblasts and growth cones, it has been proposed that microdomains of β-actin translation give rise to spatially confined pools of newly synthesized β-actin sufficiently concentrated to act as nucleation sites for the polymerization of new actin filaments that, in turn, bias the direction of migration (Katz et al., 2016; Kislauskis et al., 1997; Leung et al., 2006; Shestakova et al., 2001). Our experimental evidence showing the progressive accumulation of newly synthesized β-actin in microdomains over 5 min supports this idea. Moreover, since nascent β-actin lacks post-translational modifications, it may be a particularly potent driver of polymerization (Karakozova et al., 2006; Lin and Holt, 2007; Wang et al., 2001). Newly synthesized glutamate receptors and β-actin also accumulate in hotspots in dendrites in hippocampal neurons in vitro (Kim et al., 2013; Yoon et al., 2016). This contrasts with the more broadly distributed pattern of newly synthesized β-actin seen in axonal growth cones in culture (Ströhl et al., 2017), hinting at potential differences in terms of either nascent β-actin accumulation or the nature of translation (e.g., monosomal versus polysomal). The ability to extend a new branch based on LPS enables "on-site" and "on-demand" provision of the structural substrate needed for new branch formation and synaptogenesis in response to signals from target cell.

Impoverished neuronal arborization is a structural correlate of several neurodevelopmental disorders, such as autism and Down syndrome (Jan and Jan, 2010). However, previous studies have focused on dendrite rather than axon arborization and have not evaluated the contribution of *local* translation. Dysregulated translation, both too much or too little, can have profound effects on both axonal and dendritic branching and synapse formation (Chihara et al., 2007; Jaworski et al., 2005; Santini et al., 2013). The demonstration here that axonal arborization is disrupted by a loss of β-actin translation implicates RNA localization and local translation broadly in wiring the nervous system and raises the possibility that axonal, as well as dendritic, arborization defects underlie some neurodevelopmental disorders.

STAR★METHODS

Detailed methods are provided in the online version of this paper and include the following:

- KEY RESOURCES TABLE
- CONTACT FOR REAGENT AND RESOURCE SHARING
- EXPERIMENTAL MODEL AND SUBJECT DETAILS
  - *Xenopus laevis* Embryos Maintenance
- METHOD DETAILS
  - DNA Constructs and Morpholino
  - Electroporation
  - In Vivo Imaging
  - Blastomere Injection
  - RNA Extraction
  - Biotin-RNA Pull-Down Assay (Low Stringency)
  - Biotin-RNA Pull-Down Assay (High Stringency)
  - RNA Analysis
  - RT-qPCR
  - Mitochondrial and Genomic DNA Extraction
  - Biotin-DNA Pull-Down Assay and DNA Analysis
  - Retinal Explant Culture and Imaging
  - Protein Synthesis Inhibitor Incubation
  - In Vivo Puromycin-Based Translation Assay
  - Western Blot
  - DiI Injection, Ventral and Lateral Imaging Preparations
  - In Vitro Transcription
  - In Vivo FRAP of β-actin Translation Reporter
- QUANTIFICATION AND STATISTICAL ANALYSIS
  - General Definition and Statistics
  - RNA, RT-qPCR, and DNA Analyses
  - RNA and Mitochondrial Dynamics
  - RNA and Mitochondria Colocalization Analysis
  - In Vivo Puromycin-Based Translation Assay
  - Western Blot Analysis
  - Axon Bundle Length and Width Analyses
  - Branching Dynamics
  - Axon Navigation
  - Axon Arbor Complexity Analysis
  - FRAP Analyses

SUPPLEMENTAL INFORMATION

Supplemental Information includes seven figures and eleven movies and can be found with this article online at http://dx.doi.org/10.1016/j.neuron.2017.07.016.

AUTHOR CONTRIBUTIONS

H.H.-W.W. and C.E.H. conceived the project and wrote the manuscript. H.H.-W.W. designed the experiments. H.H.-W.W. performed and analyzed experiments, except for those presented in Figures 7 and 8 (H.H.-W.W. and F.S.),

Figure S1F (H.H.-W.W. and C.G.R.), Figure S3F (J.-M.C.), and Figures S5J–S5M (J.Q.L.). R.C., B.T.-B., R.F.L., and C.F.K. provided research tools. W.A.H. and C.E.H. supervised the project.

ACKNOWLEDGMENTS

We thank M. Coleman for sharing the mito-GFP construct; A. Mele and J.K. Mooslehner for technical assistance; and X. Almeida, A. Bellon, and V. Urbančič for critical reading of the manuscript. This work was supported by Cambridge Trust, Croucher Foundation, Sir Edward Youde Memorial Fund (H.H.-W.W.), Gates Cambridge (J.Q.L.), Fundação para a Ciência e Tecnologia (C.M.R.), Wellcome Trust Senior Investigator Award (100329/Z/12/Z) (W.A.H.), EPSRC Grant (EP/H018301/1), MRC Grant (MR/K015850/1 and MR/K02292X/1), Wellcome Trust (089703/Z/09/Z) (C.F.K.), Wellcome Trust Programme Grant (085314/Z/08/Z), and ERC Advanced Grant (322817) (C.E.H.).

Received: January 29, 2017
Revised: June 9, 2017
Accepted: July 14, 2017
Published: August 3, 2017

REFERENCES

Aakalu, G., Smith, W.B., Nguyen, N., Jiang, C., and Schuman, E.M. (2001). Dynamic visualization of local protein synthesis in hippocampal neurons. Neuron *30*, 489–502.

Alsina, B., Vu, T., and Cohen-Cory, S. (2001). Visualizing synapse formation in arborizing optic axons in vivo: dynamics and modulation by BDNF. Nat. Neurosci. *4*, 1093–1101.

Antecol, M.H., Darveau, A., Sonenberg, N., and Mukherjee, B.B. (1986). Altered biochemical properties of actin in normal skin fibroblasts from individuals predisposed to dominantly inherited cancers. Cancer Res. *46*, 1867–1873.

Bassell, G.J., Zhang, H., Byrd, A.L., Femino, A.M., Singer, R.H., Taneja, K.L., Lifshitz, L.M., Herman, I.M., and Kosik, K.S. (1998). Sorting of beta-actin mRNA and protein to neurites and growth cones in culture. J. Neurosci. *18*, 251–265.

Baudet, M.L., Zivraj, K.H., Abreu-Goodger, C., Muldal, A., Armisen, J., Blenkiron, C., Goldstein, L.D., Miska, E.A., and Holt, C.E. (2011). miR-124 acts through CoREST to control onset of Sema3A sensitivity in navigating retinal growth cones. Nat. Neurosci. *15*, 29–38.

Beach, D.L., Salmon, E.D., and Bloom, K. (1999). Localization and anchoring of mRNA in budding yeast. Curr. Biol. *9*, 569–578.

Bellon, A., Iyer, A., Bridi, S., Lee, F.C., Ovando-Vázquez, C., Corradi, E., Longhi, S., Roccuzzo, M., Strohbuecker, S., Naik, S., et al. (2017). miR-182 regulates Slit2-mediated axon guidance by modulating the local translation of a specific mRNA. Cell Rep. *18*, 1171–1186.

Bertrand, E., Chartrand, P., Schaefer, M., Shenoy, S.M., Singer, R.H., and Long, R.M. (1998). Localization of ASH1 mRNA particles in living yeast. Mol. Cell *2*, 437–445.

Brittis, P.A., Lu, Q., and Flanagan, J.G. (2002). Axonal protein synthesis provides a mechanism for localized regulation at an intermediate target. Cell *110*, 223–235.

Campbell, D.S., and Holt, C.E. (2001). Chemotropic responses of retinal growth cones mediated by rapid local protein synthesis and degradation. Neuron *32*, 1013–1026.

Chia, P.H., Chen, B., Li, P., Rosen, M.K., and Shen, K. (2014). Local F-actin network links synapse formation and axon branching. Cell *156*, 208–220.

Chien, C.B., Rosenthal, D.E., Harris, W.A., and Holt, C.E. (1993). Navigational errors made by growth cones without filopodia in the embryonic Xenopus brain. Neuron *11*, 237–251.

Chihara, T., Luginbuhl, D., and Luo, L. (2007). Cytoplasmic and mitochondrial protein translation in axonal and dendritic terminal arborization. Nat. Neurosci. *10*, 828–837.

Cohen-Cory, S., and Fraser, S.E. (1995). Effects of brain-derived neurotrophic factor on optic axon branching and remodelling in vivo. Nature *378*, 192–196.

Colak, D., Ji, S.J., Porse, B.T., and Jaffrey, S.R. (2013). Regulation of axon guidance by compartmentalized nonsense-mediated mRNA decay. Cell *153*, 1252–1265.

Condeelis, J., and Singer, R.H. (2005). How and why does beta-actin mRNA target? Biol. Cell *97*, 97–110.

Cosker, K.E., Fenstermacher, S.J., Pazyra-Murphy, M.F., Elliott, H.L., and Segal, R.A. (2016). The RNA-binding protein SFPQ orchestrates an RNA regulon to promote axon viability. Nat. Neurosci. *19*, 690–696.

Courchet, J., Lewis, T.L., Jr., Lee, S., Courchet, V., Liou, D.Y., Aizawa, S., and Polleux, F. (2013). Terminal axon branching is regulated by the LKB1-NUAK1 kinase pathway via presynaptic mitochondrial capture. Cell *153*, 1510–1525.

Das, T., Payer, B., Cayouette, M., and Harris, W.A. (2003). In vivo time-lapse imaging of cell divisions during neurogenesis in the developing zebrafish retina. Neuron *37*, 597–609.

Dong, X., Shen, K., and Bülow, H.E. (2015). Intrinsic and extrinsic mechanisms of dendritic morphogenesis. Annu. Rev. Physiol. *77*, 271–300.

Donnelly, C.J., Park, M., Spillane, M., Yoo, S., Pacheco, A., Gomes, C., Vuppalanchi, D., McDonald, M., Kim, H.H., Merianda, T.T., et al. (2013). Axonally synthesized β-actin and GAP-43 proteins support distinct modes of axonal growth. J. Neurosci. *33*, 3311–3322.

Drinjakovic, J., Jung, H., Campbell, D.S., Strochlic, L., Dwivedy, A., and Holt, C.E. (2010). E3 ligase Nedd4 promotes axon branching by downregulating PTEN. Neuron *65*, 341–357.

Eng, H., Lund, K., and Campenot, R.B. (1999). Synthesis of beta-tubulin, actin, and other proteins in axons of sympathetic neurons in compartmented cultures. J. Neurosci. *19*, 1–9.

Falk, J., Drinjakovic, J., Leung, K.M., Dwivedy, A., Regan, A.G., Piper, M., and Holt, C.E. (2007). Electroporation of cDNA/Morpholinos to targeted areas of embryonic CNS in Xenopus. BMC Dev. Biol. *7*, 107.

Frangi, A.F., Niessen, W.J., Vincken, K.L., and Viergever, M.A. (1998). Multiscale vessel enhancement filtering. In Medical Image Computing and Computer-Assisted Intervention—MICCAI'98, W.M. Wells, A. Colchester, and S.L. Delp, eds. (Springer), pp. 130–137.

Harris, W.A., Holt, C.E., and Bonhoeffer, F. (1987). Retinal axons with and without their somata, growing to and arborizing in the tectum of Xenopus embryos: a time-lapse video study of single fibres in vivo. Development *101*, 123–133.

Hörnberg, H., Wollerton-van Horck, F., Maurus, D., Zwart, M., Svoboda, H., Harris, W.A., and Holt, C.E. (2013). RNA-binding protein Hermes/RBPMS inversely affects synapse density and axon arbor formation in retinal ganglion cells in vivo. J. Neurosci. *33*, 10384–10395.

Hu, B., Nikolakopoulou, A.M., and Cohen-Cory, S. (2005). BDNF stabilizes synapses and maintains the structural complexity of optic axons in vivo. Development *132*, 4285–4298.

Jan, Y.N., and Jan, L.Y. (2010). Branching out: mechanisms of dendritic arborization. Nat. Rev. Neurosci. *11*, 316–328.

Jaworski, J., Spangler, S., Seeburg, D.P., Hoogenraad, C.C., and Sheng, M. (2005). Control of dendritic arborization by the phosphoinositide-3'-kinase-Akt-mammalian target of rapamycin pathway. J. Neurosci. *25*, 11300–11312.

Job, C., and Eberwine, J. (2001). Identification of sites for exponential translation in living dendrites. Proc. Natl. Acad. Sci. USA *98*, 13037–13042.

Kalil, K., and Dent, E.W. (2014). Branch management: mechanisms of axon branching in the developing vertebrate CNS. Nat. Rev. Neurosci. *15*, 7–18.

Kalous, A., Stake, J.I., Yisraeli, J.K., and Holt, C.E. (2014). RNA-binding protein Vg1RBP regulates terminal arbor formation but not long-range axon navigation in the developing visual system. Dev. Neurobiol. *74*, 303–318.

Karakozova, M., Kozak, M., Wong, C.C., Bailey, A.O., Yates, J.R., 3rd, Mogilner, A., Zebroski, H., and Kashina, A. (2006). Arginylation of beta-actin regulates actin cytoskeleton and cell motility. Science *313*, 192–196.

Katz, Z.B., English, B.P., Lionnet, T., Yoon, Y.J., Monnier, N., Ovryn, B., Bathe, M., and Singer, R.H. (2016). Mapping translation 'hot-spots' in live cells by tracking single molecules of mRNA and ribosomes. eLife *5*, 5.

Kim, T.K., Sul, J.Y., Helmfors, H., Langel, U., Kim, J., and Eberwine, J. (2013). Dendritic glutamate receptor mRNAs show contingent local hotspot-dependent translational dynamics. Cell Rep. *5*, 114–125.

Kislauskis, E.H., Zhu, X., and Singer, R.H. (1997). Beta-actin messenger RNA localization and protein synthesis augment cell motility. J. Cell Biol. *136*, 1263–1270.

Konopacki, F.A., Wong, H.H., Dwivedy, A., Bellon, A., Blower, M.D., and Holt, C.E. (2016). ESCRT-II controls retinal axon growth by regulating DCC receptor levels and local protein synthesis. Open Biol. *6*, 150218.

Leung, K.M., van Horck, F.P., Lin, A.C., Allison, R., Standart, N., and Holt, C.E. (2006). Asymmetrical beta-actin mRNA translation in growth cones mediates attractive turning to netrin-1. Nat. Neurosci. *9*, 1247–1256.

Lin, A.C., and Holt, C.E. (2007). Local translation and directional steering in axons. EMBO J. *26*, 3729–3736.

Longair, M.H., Baker, D.A., and Armstrong, J.D. (2011). Simple Neurite Tracer: open source software for reconstruction, visualization and analysis of neuronal processes. Bioinformatics *27*, 2453–2454.

Manitt, C., Nikolakopoulou, A.M., Almario, D.R., Nguyen, S.A., and Cohen-Cory, S. (2009). Netrin participates in the development of retinotectal synaptic connectivity by modulating axon arborization and synapse formation in the developing brain. J. Neurosci. *29*, 11065–11077.

Marshak, S., Nikolakopoulou, A.M., Dirks, R., Martens, G.J., and Cohen-Cory, S. (2007). Cell-autonomous TrkB signaling in presynaptic retinal ganglion cells mediates axon arbor growth and synapse maturation during the establishment of retinotectal synaptic connectivity. J. Neurosci. *27*, 2444–2456.

Miura, K., Rueden, C., Hiner, M., Schindelin, J., and Rietdorf, J. (2014). ImageJ Plugin CorrectBleach V2.0.2 (Zenodo). http://dx.doi.org/10.5281/zenodo.596358.

Meyer, M.P., and Smith, S.J. (2006). Evidence from in vivo imaging that synaptogenesis guides the growth and branching of axonal arbors by two distinct mechanisms. J. Neurosci. *26*, 3604–3614.

Nieuwkoop, P.D., and Faber, J. (1967). Normal Table of Xenopus laevis (Daudin): A Systematical and Chronological Survey of the Development from the Fertilized Egg Till the End of Metamorphosis (North-Holland Pub. Co.).

O'Rourke, N.A., Cline, H.T., and Fraser, S.E. (1994). Rapid remodeling of retinal arbors in the tectum with and without blockade of synaptic transmission. Neuron *12*, 921–934.

Pan, L., Zhang, Y.Q., Woodruff, E., and Broadie, K. (2004). The Drosophila fragile X gene negatively regulates neuronal elaboration and synaptic differentiation. Curr. Biol. *14*, 1863–1870.

Piper, M., Anderson, R., Dwivedy, A., Weinl, C., van Horck, F., Leung, K.M., Cogill, E., and Holt, C. (2006). Signaling mechanisms underlying Slit2-induced collapse of Xenopus retinal growth cones. Neuron *49*, 215–228.

Piper, M., Lee, A.C., van Horck, F.P., McNeilly, H., Lu, T.B., Harris, W.A., and Holt, C.E. (2015). Differential requirement of F-actin and microtubule cytoskeleton in cue-induced local protein synthesis in axonal growth cones. Neural Dev. *10*, 3.

Poggi, L., Vitorino, M., Masai, I., and Harris, W.A. (2005). Influences on neural lineage and mode of division in the zebrafish retina in vivo. J. Cell Biol. *171*, 991–999.

Preibisch, S., Saalfeld, S., Schindelin, J., and Tomancak, P. (2010). Software for bead-based registration of selective plane illumination microscopy data. Nat. Methods *7*, 418–419.

Roque, C.G., Wong, H.H., Lin, J.Q., and Holt, C.E. (2016). Tumor protein Tctp regulates axon development in the embryonic visual system. Development *143*, 1134–1148.

Ruthazer, E.S., Li, J., and Cline, H.T. (2006). Stabilization of axon branch dynamics by synaptic maturation. J. Neurosci. *26*, 3594–3603.

Santini, E., Huynh, T.N., MacAskill, A.F., Carter, A.G., Pierre, P., Ruggero, D., Kaphzan, H., and Klann, E. (2013). Exaggerated translation causes synaptic and behavioural aberrations associated with autism. Nature *493*, 411–415.

Schindelin, J., Arganda-Carreras, I., Frise, E., Kaynig, V., Longair, M., Pietzsch, T., Preibisch, S., Rueden, C., Saalfeld, S., Schmid, B., et al. (2012). Fiji: an open-source platform for biological-image analysis. Nat. Methods *9*, 676–682.

Schmidt, E.K., Clavarino, G., Ceppi, M., and Pierre, P. (2009). SUnSET, a nonradioactive method to monitor protein synthesis. Nat. Methods *6*, 275–277.

Shestakova, E.A., Singer, R.H., and Condeelis, J. (2001). The physiological significance of beta -actin mRNA localization in determining cell polarity and directional motility. Proc. Natl. Acad. Sci. USA *98*, 7045–7050.

Shigeoka, T., Jung, H., Jung, J., Turner-Bridger, B., Ohk, J., Lin, J.Q., Amieux, P.S., and Holt, C.E. (2016). Dynamic axonal translation in developing and mature visual circuits. Cell *166*, 181–192.

Spillane, M., Ketschek, A., Donnelly, C.J., Pacheco, A., Twiss, J.L., and Gallo, G. (2012). Nerve growth factor-induced formation of axonal filopodia and collateral branches involves the intra-axonal synthesis of regulators of the actin-nucleating Arp2/3 complex. J. Neurosci. *32*, 17671–17689.

Spillane, M., Ketschek, A., Merianda, T.T., Twiss, J.L., and Gallo, G. (2013). Mitochondria coordinate sites of axon branching through localized intra-axonal protein synthesis. Cell Rep. *5*, 1564–1575.

Ströhl, F., Lin, J.Q., Laine, R.F., Wong, H.H., Urbančič, V., Cagnetta, R., Holt, C.E., and Kaminski, C.F. (2017). Single molecule translation imaging visualizes the dynamics of local β-actin synthesis in retinal axons. Sci. Rep. *7*, 709.

Tatavarty, V., Ifrim, M.F., Levin, M., Korza, G., Barbarese, E., Yu, J., and Carson, J.H. (2012). Single-molecule imaging of translational output from individual RNA granules in neurons. Mol. Biol. Cell *23*, 918–929.

Tcherkezian, J., Brittis, P.A., Thomas, F., Roux, P.P., and Flanagan, J.G. (2010). Transmembrane receptor DCC associates with protein synthesis machinery and regulates translation. Cell *141*, 632–644.

Tucker, B., Richards, R.I., and Lardelli, M. (2006). Contribution of mGluR and Fmr1 functional pathways to neurite morphogenesis, craniofacial development and fragile X syndrome. Hum. Mol. Genet. *15*, 3446–3458.

Wang, J., Boja, E.S., Tan, W., Tekle, E., Fales, H.M., English, S., Mieyal, J.J., and Chock, P.B. (2001). Reversible glutathionylation regulates actin polymerization in A431 cells. J. Biol. Chem. *276*, 47763–47766.

Witte, S., Stier, H., and Cline, H.T. (1996). In vivo observations of timecourse and distribution of morphological dynamics in Xenopus retinotectal axon arbors. J. Neurobiol. *31*, 219–234.

Wu, B., Eliscovich, C., Yoon, Y.J., and Singer, R.H. (2016). Translation dynamics of single mRNAs in live cells and neurons. Science *352*, 1430–1435.

Yao, J., Sasaki, Y., Wen, Z., Bassell, G.J., and Zheng, J.Q. (2006). An essential role for beta-actin mRNA localization and translation in Ca2+-dependent growth cone guidance. Nat. Neurosci. *9*, 1265–1273.

Yoon, Y.J., Wu, B., Buxbaum, A.R., Das, S., Tsai, A., English, B.P., Grimm, J.B., Lavis, L.D., and Singer, R.H. (2016). Glutamate-induced RNA localization and translation in neurons. Proc. Natl. Acad. Sci. USA *113*, E6877–E6886.

STAR★METHODS

KEY RESOURCES TABLE

REAGENT or RESOURCE	SOURCE	IDENTIFIER
Antibodies		
Alexa488 conjugated anti-puromycin	Millipore	Cat# MABE343-AF488; RRID: AB_2566826
Rabbit anti-β-actin	Abcam	Cat# ab8227; RRID: AB_2305186
Rabbit anti-β-catenin	Sigma-Aldrich	Cat# C2206; RRID: AB_476831
HRP-conjugated secondary antibodies	Abcam	Cat# ab97080; RRID: AB_10679808
Chemicals, Peptides, and Recombinant Proteins		
Cycloheximide	Sigma-Aldrich	Cat# C4859
Anisomycin	Sigma-Aldrich	Cat# A9789
Cy5-UTP	PerkinElmer	Cat# NEL583001EA
Biotin-11-UTP	PerkinElmer	Cat# NEL543001EA
1,1'-Dioctadecyl-3,3,3',3'-Tetramethylindocarbocyanine Perchlorate (DiI)	Thermo Fisher Scientific	Cat# D282
Critical Commercial Assays		
RNeasy Mini Kit	QIAGEN	Cat# 74104
SuperScript III First-Strand Synthesis System	Thermo Fisher Scientific	Cat# 18080051
QuikChange II Site-directed Mutagenesis Kit	Agilent Technologies	Cat# 200555
QIAshredder	QIAGEN	Cat# 79654
RNA 6000 Pico Kit	Agilent Technologies	Cat# 5067-1513
QuantiTect SYBR Green PCR kit	QIAGEN	Cat# 204141
QIAamp DNA Mini Kit	QIAGEN	Cat# 51304
QIAquick PCR Purification Kit	QIAGEN	Cat# 28104
High Sensitivity DNA Kit	Agilent Technologies	Cat# 5067-4626
mMESSAGE mMACHINE SP6 Transcription Kit	Thermo Fisher Scientific	Cat# AM1340
Poly(A) Tailing Kit	Thermo Fisher Scientific	Cat# AM1350
Experimental Models: Organisms/Strains		
X. laevis	Nasco	https://www.enasco.com/product/LM00715MX/; https://www.enasco.com/product/LM00535MX/
Oligonucleotides		
Morpholino: β-actin MO 5'-CAATATCGTCTTCCATTGTGATCTG-3'	Leung et al., 2006; Gene Tools	Xenbase, RRID: SCR_003280: XB-MORPHOLINO-17249112
Morpholino: Control MO 5'-CCTCTTACCTCAGTTACAATTTATA-3'	Gene Tools	N/A
Primer: *actb* 5' UTR, Venus and the linker 5'-TACTCGGATCCGGCTCAGTGACCCGCCCGCATAGAAAGGAGACAGTCTGTGTGCGTCCAACCCTCAGATCACAATGGTTAGTAAGGGCG-3' 5'-GTATGAATTCAAGCTTTTTGTAAAGTTCATCC-3'	Sigma-Aldrich	N/A
Primer: linker, *actb* CDS, *actb* 3' UTR 5'-GCTTGAATTCAAAATGGAAGACGATATTG-3' 5'-CGTAGCGGCCGCGTGAAACAACATAAGT-3'	Sigma-Aldrich	N/A
Primer: morpholino insensitive *actb* 5'-CAACCCTCAGATCACAATGGAGGATGACATAGCCGCACTGGTCGTTG-3'	Sigma-Aldrich	N/A

(*Continued on next page*)

Continued

REAGENT or RESOURCE	SOURCE	IDENTIFIER
Primers for *rps13* RT-qPCR: 5'-CTTCAAACTGGCCA AGAAGG-3' 5'-GGCCAGAGCCTTAGACTTGA-3'	Sigma-Aldrich	N/A
Primers for *actb* RT-qPCR: 5'-TACTCTTTTGTTGGC GCTTG-3' 5'-GGGCAACACTGAGAGGGTAG-3'	Sigma-Aldrich	N/A
Primers for *sdha* RT-qPCR: 5'-AGACTCAACATGCAG AAGACCA-3' 5'-TCCATTGCAGAATTGATGACAC-3'	Sigma-Aldrich	N/A
Recombinant DNA		
pCS2+mGFP	Das et al., 2003	N/A
pCS2+mRFP	Poggi et al., 2005	N/A
Mito-GFP	Michael Coleman (Department of Clinical Neuroscience, University of Cambridge, UK)	N/A
pCS2+MO insensitive β-actin/mGFP dual promoter construct	This paper	N/A
pCS2+Venus-β-actin	This paper	N/A
pCS2+Venus	This paper	N/A
Software and Algorithms		
Fiji	Schindelin et al., 2012	Fiji, RRID: SCR_002285
Descriptor-based registration	Preibisch et al., 2010	http://imagej.net/SPIM_Registration
Simple Neurite Tracer	Longair et al., 2011	https://imagej.net/Simple_Neurite_Tracer
Colocalization test	Tony Collins	https://imagej.net/Colocalization_Test
Bleach Correction	Miura et al., 2014	https://imagej.net/Bleach_Correction
Volocity	v.6.3.1	Volocity 3D Image Analysis Software, RRID: SCR_002668
GraphPad PRISM	v.6.01	GraphPad Prism, RRID: SCR_002798
MATLAB	v.R2015b	MATLAB, RRID: SCR_001622
Batch Analysis of Mean and SD. Evolution	This paper; Cambridge repository server	https://doi.org/10.17863/CAM.9542

CONTACT FOR REAGENT AND RESOURCE SHARING

Further information and requests for resources and reagents should be directed to and will be fulfilled by the Lead Contact, Christine E. Holt (ceh33@cam.ac.uk).

EXPERIMENTAL MODEL AND SUBJECT DETAILS

Xenopus laevis Embryos Maintenance

Xenopus laevis embryos obtained from in vitro fertilization were raised in 0.1X Modified Barth's saline (MBS; 8.8mM NaCl, 0.1mM KCl, 82 μM $MgSO_4$, 0.24mM $NaHCO_3$, 0.1mM HEPES, 33 μM $Ca(NO_3)_2$, 41 μM $CaCl_2$) at 14-22°C, and staged according to the table of Nieuwkoop and Faber (Nieuwkoop and Faber, 1967). This research has been regulated under the Animals (Scientific Procedures) Act 1986 Amendment Regulations 2012 following ethical review by the University of Cambridge Animal Welfare and Ethical Review Body (AWERB).

METHOD DETAILS

DNA Constructs and Morpholino

The plasmids pCS2+mGFP (Das et al., 2003) and pCS2+mRFP (Poggi et al., 2005) were obtained as previously described. Mitochondria-targeted GFP (mito-GFP) was a gift from Michael Coleman (Department of Clinical Neuroscience, University of Cambridge, UK).

Total mRNA extracted from Stage 32 embryos using RNeasy Mini Kit (QIAGEN) was reverse transcribed into cDNA library with SuperScript III First-Strand Synthesis System (ThermoFisher) using Oligo(dT) as primer. To obtain the Venus-β-actin fusion construct, full-length *actb* including 5'UTR, coding sequence and 3'UTR obtained from the cDNA library by PCR was cloned into pCS2+ vector with the constitutive CMV promoter. The monomeric Venus coding sequence was inserted between *actb* 5'UTR and the coding sequence, followed by a short linker (KLEFK). To construct the Venus control plasmid, *actb* 5'UTR, the coding sequence and the majority of the 3'UTR were deleted from the Venus-β-actin construct except the last 46 nucleotides, leaving the polyadenylation signal sequence and a short stretch of poly(A) in place. For the morpholino insensitive β-actin/mGFP dual promoter construct, full-length *actb* was cloned into the multiple cloning site of pCS2+ plasmid. The β-actin morpholino targeted sequence at the first 16bp of the coding sequence was mutated with QuikChange II Site-directed Mutagenesis Kit (Agilent Technologies) with the primer (5'-CAACCCTCAGATCACAATGGAGGATGACATAGCCGCACTGGTCGTTG-3'). The β-actin amino acid sequence encoded remained the same and only the codons were changed. mGFP driven by the eIF1α constitutive promoter was cloned immediately upstream of the CMV promoter in reverse orientation.

The β-actin antisense morpholino (5'-CAATATCGTCTTCCATTGTGATCTG-3') as previously described (Leung et al., 2006) and the control antisense morpholino (5'-CCTCTTACCTCAGTTACAATTTATA-3') conjugated to fluorescein at the 3' end were supplied by Gene Tools.

Electroporation

Targeted eye electroporation was performed as previously described (Falk et al., 2007). Stage 28 embryos were anesthetized in 0.4mg/ml MS222 in 1X MBS. The retinal primordium was injected with electroporation mixture, followed by electric pulses of 50ms duration at 1000ms intervals, delivered at 18V (please refer to the list below for the mixture and the number of electric pulses delivered for each experiment). The embryos were recovered and raised in 0.1X MBS until the desired embryonic stage for experiment.

Electroporation mixtures and number of electric pulses delivered:

1) RNA dynamics (Figures 1 and S1): 5mM Cy5-UTP (PerkinElmer), 1 μg/μl of pCS2+mGFP; 4 pulses.
2) Mitochondria dynamics (Figure S2): 1 μg/μl of pCS2+mito-GFP, 1 μg/μl of pCS2+mRFP; 4 pulses.
3) Cy5-RNA and mito-GFP colocalization (Figure S3): 5mM Cy5-UTP, 1 μg/μl of pCS2+mito-GFP, 1 μg/μl of pCS2+mRFP; 4 pulses.
3) Axon navigation (Figures 3 and S6): 1 μg/μl of pCS2+mGFP or 1 μg/μl of pCS2+mRFP, (+/– 0.5mM control/β-actin MO); 8 pulses.
4) Mature axon arbor visualization (Figures 4A–4G): 0.5 μg/μl of pCS2+mGFP, 0.5mM control/β-actin MO; 1 pulse.
5) Axon branching dynamics (Figures 2, 4H–4M, 5, and S4): 1 μg/μl of pCS2+mGFP (or 1 μg/μl pCS2+mGFP/MO resistant β-actin dual promoter construct cDNA was used in Figure 4J for rescue experiment) or 1 μg/μl of pCS2+mRFP, (+/– 0.5mM control/0.5mM β-actin MO/1μM MO resistant β-actin mRNA); 4 pulses.
6) FRAP experiments (Figures 6, 7, 8, and S7): 1 μg/μl of pCS2+mRFP, 1 μg/μl of pCS2+Venus/ pCS2+Venus-β-actin, (+/– 5mM Cy5-UTP); 4 pulses.

For the pathway (Stages 35/36-37/38; Figure S6) and tectum (Stages 41-43; Figures 5A–5E, Stages 35/36-37/38; Figures 5F–5J) electroporation, the lateral surface of the hemisphere of the brain contralateral to the eye labeled with mRFP (electroporated at Stage28 as described above) was exposed by careful removal of overlying eye and epidermis (Chien et al., 1993). 8X 18V electric pulses of 50ms duration at 1000ms intervals were delivered immediately after the 1mM control/β-actin MO was locally ejected at the vicinity of the target area. The procedure was repeated once to ensure efficient delivery of the MO. For the local rescue experiment (Figure S4), 0.5mM β-actin MO (+/– 1μM β-actin MO resistant β-actin mRNA) was added to the electroporation mix.

In Vivo Imaging

Embryos were lightly anaesthetized with 0.4mg/ml MS222 in 1X MBS. The lateral surface of the brain contralateral to the electroporated eye was exposed by removal of the overlying epidermis and the contralateral eye (Chien et al., 1993). The electroporated eyes were also surgically removed to prevent somal contribution of proteins in Figures 2, 3, 5A–5E, 6, S4B–S4G, S6A–S6F, and S7. Embryos were mounted in an oxygenated chamber created with Permanox slides (Sigma-Aldrich) and Gene Frame (ThermoFisher), and bathed in 1X MBS with 0.1mg/ml MS222, for visualization with fluorescence microscopy. Imaging related to axonal branching was performed using 40X (NA 1.25) or 60X UPLSAPO objectives (NA 1.3) with a PerkinElmer Spinning Disk UltraVIEW ERS, Olympus IX81 inverted spinning disk confocal microscope. Imaging of axon navigation in the optic tract was performed with Plan Fluor 20X (NA 0.5) using a Nikon Eclipse TE2000-U inverted microscope. Z stack intervals of 1-2μm were employed for acquiring images with Volocity (PerkinElmer).

Blastomere Injection

Embryos were injected at the 4-cell stage in the dorsal animal blastomeres. Injections were performed using glass capillary needles (1.0 mm outer diameter (OD) x 0.5 mm internal diameter (ID), Harvard Apparatus) and a pressurized microinjector (Picospritzer,

General Valve). For the culture experiment shown in Figure S3F, Cy5-UTP was injected at 100μM and Mito-GFP was injected at a concentration of [50ng/μl] in a total volume of 5nl. For biotin-RNA/DNA pull-down experiments, 5nl of 5mM biotin-11-UTP (PerkinElmer) per blastomere was used. For experiments shown in Figure S5, 5nl of [2 μg/μl] of control/β-actin MO were injected into each blastomere.

RNA Extraction

Stages 40/41 embryos were anaesthetized in 0.4mg/ml MS222 in 1X MBS, and decapitated to harvest total RNA using the QIAshredder and RNeasy Mini Kit (QIAGEN) from whole heads. RNA was extracted following manufacturer's instructions with on-column DNase digestion. The RNA Integrity Numbers (RINs) for control and biotin-UTP samples were 9.4 ± 0.4 and 9.6 ± 0.2, respectively. These values indicate that the extracted RNA samples were of high quality.

Biotin-RNA Pull-Down Assay (Low Stringency)

150 μL of Streptavidin Mag Sepharose (GE Healthcare) slurry was transferred into two separate Eppendorf tubes. The beads were washed three times with 1ml of buffer L1 (1X PBS pH7.4, 0.02% Tween-20). The beads were then incubated in 1125 μL of buffer L1 and 125 μL of 5X Denhardt's Solution (Sigma-Aldrich) for 1 hr, and subsequently washed three times in buffer L2 (0.3X SSPE, 1mM EDTA, 0.05% Tween-20). An equal amount (~40%, same amount as for the high stringency protocol as described below) of the total RNA harvested from 100 whole heads of uninjected control/biotin-11-UTP injected embryos was added to each tube containing the beads and the volume was brought up to 200 μL with buffer L2. The RNA was incubated with the beads at 4°C for 2 hr before being washed three times with buffer L2, six times with buffer L3 (15mM Tris pH7.5, 5mM EDTA) and six times with buffer L4 (15mM Tris pH7.5, 5mM EDTA, 2.5mM EGTA, 1% Triton X-100, 1% NaDOC, 0.1% SDS, 120mM NaCl, 25mM KCl). For competitive elution of the bound molecules, 200 μl of 2.5mM biotin solution was added to the beads and was heated at 95°C for 4 min. The eluents were then purified with the RNeasy-mini kit. To increase the concentration of the RNA samples for bioanalyzer RNA analysis, 15 μL of RNase-free water was used for eluting the RNA from the RNeasy-mini columns, with the eluents from the first round of elution rerun into the column for the second round.

Biotin-RNA Pull-Down Assay (High Stringency)

150 μL of Streptavidin Mag Sepharose slurry was transferred into two separate tubes. The beads were washed three times with 1ml of buffer H1 (1M NaCl, 20mM Tris pH 7.3, 5mM EDTA pH8, 0.1% NP-40). The beads were then incubated in 1ml of buffer H1 for 1 hr. Buffer H1 was removed and an equal amount (~40%, same amount as for the low stringency protocol as described above) of the total RNA harvested from 100 whole heads of uninjected control/biotin-11-UTP injected embryos were added into each tube containing the beads. The volume was brought up to 200 μL with buffer H1. The RNA were incubated with the beads at 4°C for 2 hr before being washed three times with buffer H1, six times with buffer H2 (2mM Tris pH7.3, 0.5mM EDTA pH8, 0.1% NP-40), six times with buffer H3 (4M urea, 10mM Tris pH7.3, 1mM EDTA pH8, 0.1% NP-40) and six times with buffer H4 (2mM Tris pH7.3, 0.5mM EDTA pH8). For elution and purification of the bound molecules, the same procedures as for the low stringency pull-down assay were carried out.

RNA Analysis

Analyses of purified RNA were carried out with the Agilent 2100 Bioanalyzer and RNA 6000 Pico Kit (Agilent Technologies) following the manufacturer's instructions.

RT-qPCR

RNA samples from the streptavidin pull down were reverse transcribed with the SuperScript III First-Strand Synthesis System. Triplicate 10 μL reactions were prepared according to manufacturer's instructions (QuantiTect SYBR Green PCR kit, QIAGEN). Plates were then centrifuged at 1,500 x *g* for 2 min at 4°C before commencing the cycling protocol. The PCR cycling conditions used followed the instructions provided by the manufacturer (Denaturation: 15 s at 94°C; Annealing: 30 s at 57°C; Extension: 30 s at 72°C; Data acquisition: 15 s at 72°C). Real-time PCR runs were performed on a LightCycler 480 (Roche; software release 1.5). The following primers were used; for *rps13*, 5'-CTTCAAACTGGCCAAGAAGG-3' and 5'-GGCCAGAGCCTTAGACTTGA-3'; for *actb*, 5'-TACTCTTT TGTTGGCGCTTG-3' and 5'-GGGCAACACTGAGAGGGTAG-3'; for *sdha*, 5'-AGACTCAACATGCAGAAGACCA-3' and 5'-TCCAT TGCAGAATTGATGACAC-3'.

Mitochondrial and Genomic DNA Extraction

Stages 40/41 embryos were anaesthetized in 0.4mg/ml MS222 in 1X MBS, and decapitated to harvest total DNA using the QIAamp DNA Mini Kit (QIAGEN) from whole heads. DNA was extracted following manufacturer's instructions with RNase A digestion. DNA was fragmented using the NEBNext dsDNA Fragmentase (New England BioLabs) to facilitate biotin-streptavidin binding and electrophoresis.

Biotin-DNA Pull-Down Assay and DNA Analysis

The same buffers and procedures were used as the biotin-RNA pull down experiments for both low and high stringencies. An equal amount of fragmented DNA from the control and biotin-UTP samples was used for the DNA pull down. The eluted solution from the

pull down was purified with the QIAquick PCR Purification Kit (QIAGEN). Analyses of purified DNA were carried out with the Agilent 2100 Bioanalyzer and High Sensitivity DNA Kit (Agilent Technologies) following manufacture instruction.

Retinal Explant Culture and Imaging

50 mm glass-bottom dishes (Matek) were coated with poly-L-lysine (PLL; 10 μg/ml) diluted in double distilled water (ddH$_2$O) overnight at 20°C and then washed 3 times with ddH$_2$O and dried for 1 hr, followed by coating with laminin (10 μg/ml, Sigma) in L-15 medium (GIBCO) for 1 hr at room temperature. Injected stage 33-34 embryos were washed 3 times in 0.1X MBS with 1% Antibiotic-Antimycotic (ThermoFisher). Embryos were then anesthetized with 0.4mg/ml MS222 (60% L-15 in ddH$_2$O and 1% Antibiotic-Antimycotic and MS222, pH 7.6-7.8) and secured on their lateral side with custom made pins on a Sylgard-coated dish. Both eyes were dissected out using dissection pins, washed in 60% L-15, and plated on pre-coated dishes containing 60% L-15 culture medium (60% Phenol Red-free L-15 medium supplemented with 1% Antibiotic-Antimycotic). Cultures were incubated at 20°C for 18 hr before imaging. Cultured axons were imaged for 5 min with 60X UPLSAPO objectives (NA 1.3) with a PerkinElmer Spinning Disk UltraVIEW ERS, Olympus IX81 inverted spinning disk confocal microscope. The example in Figure S3F is a time-lapse sequence captured at 12 frames/min.

Protein Synthesis Inhibitor Incubation

The intact brains of anaesthetized embryos were exposed with the labeled eye removed as described above, followed by incubation in control (0.1mg/ml MS222 in 1X MBS), DMSO, cycloheximide (100 μM) or anisomycin (160 μM) solution for 30min before imaging.

In Vivo Puromycin-Based Translation Assay

Adapted from a previously reported in vitro approach with the use of puromycylation of nascent proteins and immunolabeling of puromycin (Schmidt et al., 2009). Live embryos were incubated in 0.1mg/ml MS222 in 1X MBS with or without (for the "No Puromycylation Control") 10 μg/ml of puromycin (Sigma) for 10 min. The embryos were then washed with 10mg/100ml MS222 in 1X MBS for 5 min to remove unincorporated puromycin prior to fixation with 4% paraformaldehyde in 1X PBS at 4°C overnight. The fixed embryos were washed three times with 1X PBS for 5 min, three times with PBT (0.2% BSA, 0.5% Triton X-100, 1X PBS) for 10 min, once with PBT for 30 min before incubating with 5% heat-inactivated goat serum in PBT for 30 min. Alexa488 conjugated anti-puromycin antibody (Millipore Cat# MABE343-AF488, RRID: AB_2566826; 1:50) was incubated with the embryos at 4°C for overnight. The immunostained embryos were washed three times with PBT for 10 min, once with PBT for 30 min, twice with 1X PBS for 5 min before being mounted in 1X PBS for imaging.

Western Blot

Brains and eyes were harvested from Stages 40/41 embryos that were anesthetized with 0.4mg/ml MS222 in 1X MBS. The tissues were mechanically homogenized with radioimmunoprecipitation assay (RIPA; Sigma) buffer supplemented with protease inhibitor cocktail (Roche) by repeated pipetting, and solubilized by constant rotation at 4°C for 30 min. Remaining non-solubilized material was then pelleted by centrifugation at 13000rpm for 10 min and only the supernatant was then retained. Protein concentration was determined by Bradford assay (Bio-Rad) and spectrophotometry. Bovine serum albumin (BSA; Invitrogen) was used to create a standard curve for protein concentration and for normalizing the concentration between samples. The lysates were resolved by 12% TGX precast gels (BioRad) at constant 20mA, transferred to nitrocellulose membrane (BioRad) at constant 110V and subjected to western blot analysis by incubating with a rabbit anti-β-actin (Abcam Cat# ab8227, RRID:AB_2305186; 1:8000) or rabbit anti-β-catenin (Sigma-Aldrich Cat# C2206, RRID:AB_476831; 1:8000) antibody at 4°C for overnight. The blots were then incubated with HRP-conjugated secondary antibodies (Abcam Cat# ab97080, RRID:AB_10679808; 1:16000) in room temperature for one hour, followed by ECL-based detection (Invitrogen).

DiI Injection, Ventral and Lateral Imaging Preparations

Embryos were fixed in 4% formaldehyde in PBS at 4°C overnight. DiI solution prepared by dissolving 50 mg of DiI powder (ThermoFisher) in 1 ml of ethanol (Sigma-Aldrich) was injected into the eye cavity until completely filled (Roque et al., 2016). The embryos were incubated at 20°C overnight to ensure the dye has diffused to the distal axons before imaging was carried out.

For lateral-view imaging, the brain was dissected and mounted in 1X PBS. The contralateral brain hemisphere was imaged with confocal microscopy. For ventral-view preparation (Figure S5F), the ventral surface of the brain was exposed by carefully removing the epidermis, mesenchymal and endodermal tissue underlying the brain (Konopacki et al., 2016). The exposed brains were then mounted in ventral view with 1X PBS in order to visualize the whole optic path.

In Vitro Transcription

Capped mRNA was in vitro transcribed using mMessage mMachine SP6 Transcription Kit (ThermoFisher), and polyadenylated using Poly(A) Tailing Kit (ThermoFisher).

In Vivo FRAP of β-actin Translation Reporter

Embryos electroporated with mRFP and Venus-Poly(A)/Venus-β-actin cDNAs were raised until Stages 35/36-37/38 (Figure S7), Stages 40/41 (Figure 6) or Stages 41-43 (Figures 7 and 8) and prepared for live imaging.

For Figures 6 and S7, labeled axons were visualized with the 561nm-laser with and photobleached with a 488nm-laser on a PerkinElmer Spinning Disk UltraVIEW ERS, Olympus IX81 inverted spinning disk confocal microscope. The parameters for photobleaching were: "1" for PK cycles; "1" for PK step size; "3000-4000ms" for PK spot period; "2" for PK spot cycles; "Small" for PK spot size; "None" for PK attenuation. Images for both mRFP and Venus were captured immediately before and after photobleaching, followed by 30 s intervals post-photobleaching.

For Figure 7, imaging was performed on a custom-built inverted single-molecule fluorescence microscope built around a commercial microscope frame (Olympus IX73). The illumination laser wavelengths were 488 nm (Coherent Sapphire) for excitation of Venus in combination with a 525/45 emission filter (Semrock) and 561 nm (Cobolt Jive) for excitation of mRFP in combination with a 600/37 emission filter (Semrock). The same dichroic beam splitter (Chroma ZT405/488/561/640rpc) was used for both channels and both laser beams were circularly polarized via a quarter wave plate (Thorlabs AQWP05) to excite fluorescent proteins homogeneously regardless of their orientation. The microscope was equipped with an EM-CCD camera (Andor iXon Ultra 897) with an effective pixel size on the sample of 118 nm. A 1.49 NA oil immersion TIRF objective (Olympus UAPON100XOTIRF) was used. The acquisition protocol comprised an outline imaging step of the axonal arbor prior to photobleaching. Both accumulated Venus-β-actin/Venus and the cell morphology labeled by mRFP were imaged with low irradiance (< $2W/cm^2$) in a sequentially manner. The axon was then photobleached for 20 s with an irradiance of $1.5kW/cm^2$ using the 488 nm wavelength laser and the recovery of Venus fluorescence recorded at 1Hz for 300 s at low irradiance levels (< $2W/cm^2$) to avoid additional photodamage of the fluorescent proteins and ensure survival of the axons. A second axon outline image of the mRFP was taken directly after FRAP imaging was finished. All imaging steps were performed under episcopic illumination. Imaging was done with the full field of view of the EM-CCD camera (512 × 512 $pixel^2$), which corresponds to a region of 60 × 60 μm^2, and an additional EM gain of 200 to ensure high sensitivity.

For Figure 8, the setup as used for Figure 7 was modified to allow simultaneous dual-color imaging onto two identical EMCCD cameras (Andor iXon Ultra 897). The light was split using a TwinCam (CAIRN) housing a dichroic beam splitter (Chroma T565spxr) together with a 525/45 emission filter (Semrock) in the transmission direction to capture Venus fluorescence and a 680/42 emission filter (Semrock) in reflection to capture Cy5 fluorescence. For imaging of mRFP fluorescence, the 680/42 emission filter was replaced by a 600/37 emission filter (Semrock). The imaging sequence used was the same as for Figure 7 but with the additional recording of the Cy5-RNA granules, which were excited at 647nm (MPB VFL-P-200-647) with low irradiance (< 2W/cm2).

QUANTIFICATION AND STATISTICAL ANALYSIS

General Definition and Statistics

A filopodium was defined as a protrusion with a length < 5 μm while a branch was defined as a protrusion with a length > 5 μm (Drinjakovic et al., 2010; Hörnberg et al., 2013; Kalous et al., 2014). Data were analyzed in PRISM 6 (GraphPad). Data are presented as mean and error bars represent SEM 'n' represents the number of axons unless stated otherwise below. *p < 0.01, **p < 0.01, ***p < 0.001. ##p < 0.01, ###p < 0.001. Details of statistic results such as p values, degree of freedom, and U/t/F values are presented in the figure legends.

RNA, RT-qPCR, and DNA Analyses

For RNA analysis, the concentrations of total RNA, 18S rRNA and 28S rRNA in arbitrary units were obtained from the Agilent 2100 Bioanalyzer and RNA 6000 Pico Kit. For RT-qPCR, quantification reads (crossing points; C_p values) were analyzed with the Roche's LightCycler 480 software. Standard curves to calculate the amplification efficiency were run independently from the actual experiments. The readout of the control samples were normalized to the biotin-UTP samples to obtain relative concentrations, which were then compared with two-way ANOVA for each RNA species/category (n = 3 independent experiments). For DNA analysis, the Agilent 2100 Bioanalyzer and High Sensitivity DNA Kit did not yield any measurable signals in both control and biotin-UTP samples (n = 3 independent experiments).

RNA and Mitochondrial Dynamics

Time-lapse images were quantified for 60 min from the first instance that a branch was formed on the terminal 100 μm of GFP/RFP-labeled RGC axons ("n" represents the number of branches). Docking was defined as stationary or oscillatory movements within a distance of the diameter of the RNA granule or the mitochondrion. To assess the association between RNA/mitochondria docking behavior at the axon shaft and protrusion formation, the proportion of protrusions with a docking time longer than 10 s (RNA) or 1 min (mitochondria) prior to filopodial emergence was retrospectively quantified ("n" represents the number of protrusions). These temporal criteria were defined by at least 3 frames of time-lapse imaging (3-6 s/frame for RNA and 30 s/frame for mitochondria). Mitochondria dynamics were captured at a slower rate of 30 s per 3D-stack per frame to prevent phototoxicity as time-lapse imaging was often carried out over periods of > 1 hr. The occurrences of these docking behaviors at random sites were quantified by computing random axon positions at random time points with random number generators. The results from the protrusion-forming positions were compared to random position with paired t test. For RNA trafficking speed and direction, the terminal 70 μm of GFP-labeled

RGC axons was quantified for 1 min ("n" represents the number of RNA granules). RNA granules that moved less than 1 μm were defined as "stationary" and granules that changed the direction of trafficking at least once during the 60 s period of quantification were defined as "bi-directional."

RNA and Mitochondria Colocalization Analysis

High resolution image stacks of Cy5-RNA, mito-GFP and mRFP were imported into Volocity Quantitation package (PerkinElmer). The axons were identified in a three dimensional manner with the 'Find objects' function with mRFP signal. A three dimensional crop was performed to prevent the noise outside of axons in z-layers from being included in the colocalization analysis. The cropped image stack was imported into FIJI (Schindelin et al., 2012) and a 3D mask of axons was created from the mRFP channel with the threshold function. With the use of the FIJI plugin, Colocalization Test, Pearson's correlation coefficient R(obs) was computed for the volume within the 3D mask. Each of the image stacks were individually scrambled with Costes approximation to create 1000 random images for computing the R(rand) values. A paired t test was performed to compare R(rand)>R(obs). For fast time lapse images, 3D crops were created in Volocity Quantitation package as mentioned above. The Costes Pearson's Correlation was measured by Volocity across time (average image intervals = 5.8 ± 0.6 s per z stack). "n" represents the number of embryos.

In Vivo Puromycin-Based Translation Assay

The imaging parameters were kept the same for each round of experiment to allow background-corrected quantitative immunofluorescence comparisons across conditions. One-way ANOVA with Tukey multiple comparisons test was carried out. As an estimation of non-specific incorporation of Alexa488 conjugated anti-puromycin antibody into embryo whole brain, the 99.9% confidence intervals of the "No Puromycylation Control" was 0.016%–0.025% of the control condition (n = 18). "n" represents the number of embryos.

Western Blot Analysis

Developed films from western blot detection were scanned and imported into FIJI. The color was inverted and the background-corrected signals for β-actin and β-catenin were measured. The level of β-actin was normalized to β-catenin to yield a ratiometric readout. Paired t test was used to assess knockdown efficiency of β-actin protein level (n = 5 independent experiments).

Axon Bundle Length and Width Analyses

For lateral view imaging preparation, the optic tract length was defined by a straight line from the optic chiasm to the distal-most axon terminals in the optic tract/tectum (Roque et al., 2016). Ten equally spaced concentric circles (C1-C10) were overlaid on the tract images with the center of the circles overlying the optic chiasm and C10 overlaying the dorsal-posterior tectal boundary. Both the length and width of tract were then normalized to the brain size, which is defined by the straight line from the optic chiasm to the dorsal-posterior tectal boundary. For ventral view imaging, the optic nerve length was measured from the optic nerve head in the eye to the chiasm at the ventral midline of the brain. The length was then divided into 5 sections (S1-S5), which are points where the width of the optic nerve was measured perpendicular to the long axis. Both the length and width of the optic nerve were then normalized to the brain size, which is defined by a straight line from the point of the brain adjacent to the center of the eye to the midline of the brain. Unpaired t test was used to compare the length of the optic tract and the width of different axon sections between control and β-actin morphants. "n" represents the number of embryos.

Branching Dynamics

The number of protrusions added and removed was counted on the terminal 50 μm of mGFP/mRFP-labeled RGC axons for 10 min (imaged at an interval of 30 s). The addition and removal of protrusions were then compared statistically. Paired t test was used for intragroup comparison, unpaired t test (2 groups) or one-way ANOVA with Tukey multiple comparisons test (3 groups) was used for intergroup comparisons.

Axon Navigation

Quantification of axon growth was performed up to one hour of live imaging. Stalling was defined by axon moving less than 5 μm over 1 hr; misprojection was defined by axon navigating aberrantly away from the normal trajectory and death was defined by axon retracting or showing beaded morphology. Fisher's exact test (2 groups) or Chi-square test (3 groups) was used for comparing percentages of axons exhibiting different behaviors. Unpaired t test (2 groups) or one-way ANOVA with Tukey multiple comparisons test (3 groups) were used for axon velocity comparisons.

Axon Arbor Complexity Analysis

3D projections of axon arbors acquired at 40X were semi-automatically traced through the z axis using the Simple Neurite Tracer plugin (Longair et al., 2011) in FIJI. The resulting traces were then analyzed for the number and the length of axon branches as well as the Axon Complexity Index (ACI) (Marshak et al., 2007). These measured parameters were compared using one-way ANOVA with Tukey multiple comparisons test. The proportions of simple (ACI $<$ 1.4) and complex (ACI $\geq$ 1.4) arbors in different groups were compared using Fisher's exact test (Drinjakovic et al., 2010).

FRAP Analyses

For Figures 6 and S7, Fluorescence intensity was measured in the terminal 15 μm of RGC axons from raw images. FRAP was calculated from background-corrected fluorescence intensity by normalizing the change in fluorescence ($F-F_0$) to pre-photobleaching fluorescence (F_p). For Figure 7, the raw data was analyzed using custom-written MATLAB software and FIJI. First, the FRAP time-series was corrected for drift using descriptor-based registration (Preibisch et al., 2010) and successively, both pre-bleaching outline images as well as the post-recovery outline image were aligned to the drift-corrected FRAP image stack via the same registration transformation. Using information from all outline images, the axonal arbors were skeletonized semi-automatically via user-defined end-points and neurite-connection automatically computed using a tracer based on a vesselness filter (Frangi et al., 1998), implemented in the Simple Neurite Tracer FIJI plugin. The skeleton data was divided into the shaft of the axon and the branches, which served as masks for further data analysis. Based on these masks, the recovery of fluorescence was computed for every time point in all branches and the axon shaft for each repeat and experimental condition using custom-written MATLAB software. Axon shaft pixel values as defined by the mask were averaged, yielding a mean value and a standard deviation; pixels from all branches originating from the same axon were also averaged and the standard deviation (SD) was computed. By internally normalizing the SD of branch pixels to the SD of axon shaft pixels, the fluorescence variation index (FVI) was yielded. Extra sum-of-squares F test was used for intergroup comparisons for FRAP and FVI analyses.

For Figure 8, the FRAP and RNA time-series were both corrected for drift as described above. The RNA time-series was corrected for photobleaching with the histogram matching method (Miura et al., 2014). The corrected RNA time-series were compiled into z stacks and computed for the median signals with z-projection, this provides a representation of the dwell time of RNA granules in different position in the axons. For the FRAP time-series, the images were compiled and projected with maximum intensity to obtain the cumulative FRAP. For a quantitative evaluation of correlation between Venus recovery and RNA granule position, the resultant FRAP and RNA images were then used to calculate the Pearson's correlation coefficient R(observed) within the axon outlines with the use of the FIJI plugin Colocalization Test. Each image was individually scrambled with Costes approximation to generate 1000 random images and was used for computing the R(random) for randomly distributed fluorescence. A paired t test was performed to test for statistical significance between the differences of R(observed) and R(random).

Neuron, Volume *95*

Supplemental Information

RNA Docking and Local Translation

Regulate Site-Specific Axon Remodeling In Vivo

Hovy Ho-Wai Wong, Julie Qiaojin Lin, Florian Ströhl, Cláudio Gouveia Roque, Jean-Michel Cioni, Roberta Cagnetta, Benita Turner-Bridger, Romain F. Laine, William A. Harris, Clemens F. Kaminski, and Christine E. Holt

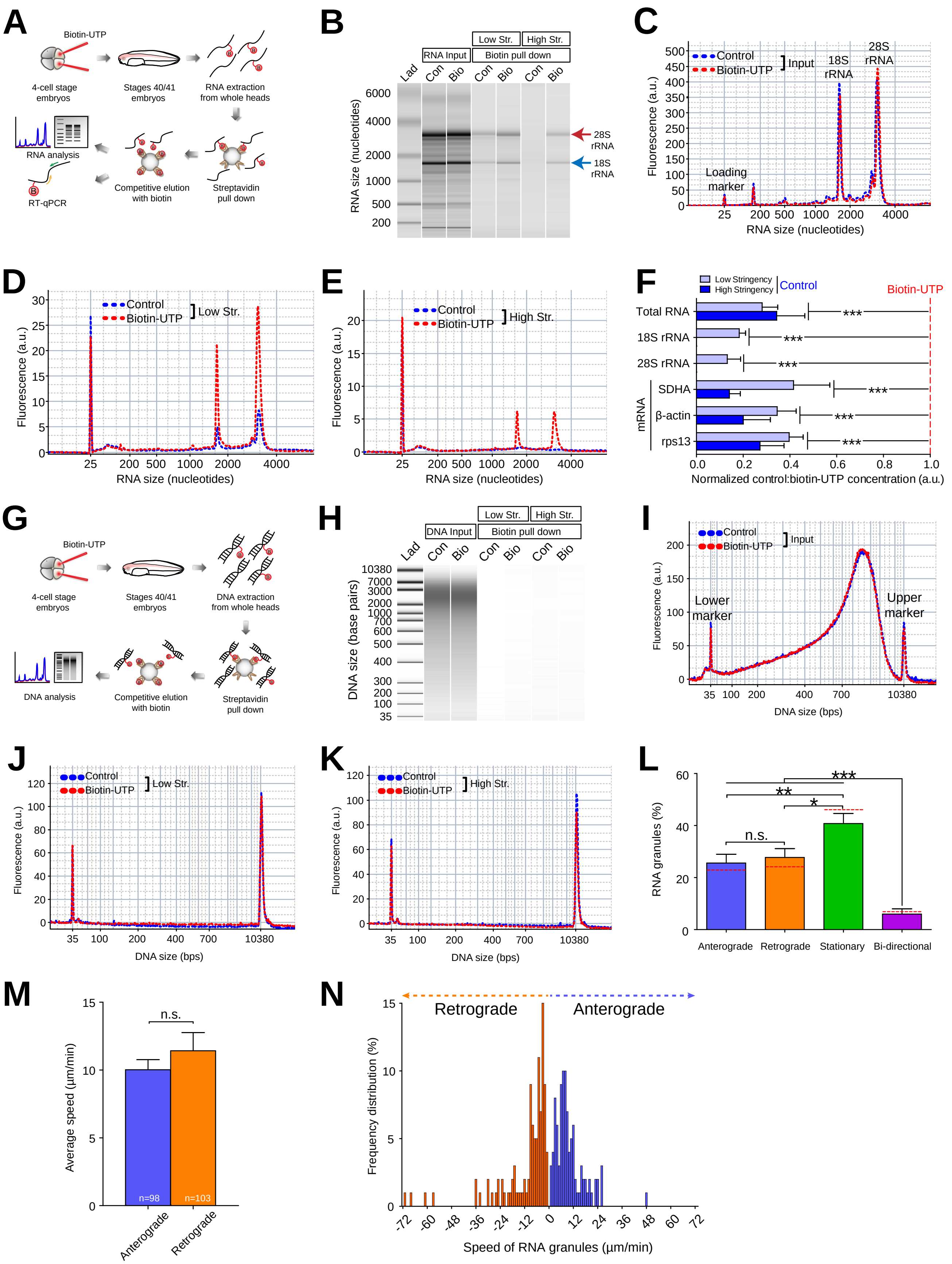
A
Biotin-UTP
4-cell stage embryos
Stages 40/41 embryos
RNA extraction from whole heads
RNA analysis
RT-qPCR
Competitive elution with biotin
Streptavidin pull down
B
Low Str.
High Str.
RNA Input
Biotin pull down
Lad
Con
Bio
RNA size (nucleotides)
28S rRNA
18S rRNA
C
Control
Biotin-UTP
Input
18S rRNA
28S rRNA
Loading marker
Fluorescence (a.u.)
RNA size (nucleotides)
D
Low Str.
E
High Str.
F
Low Stringency
High Stringency
Control
Biotin-UTP
Total RNA
18S rRNA
28S rRNA
SDHA
β-actin
rps13
mRNA
Normalized control:biotin-UTP concentration (a.u.)
G
DNA extraction from whole heads
DNA analysis
H
DNA Input
DNA size (base pairs)
I
Lower marker
Upper marker
DNA size (bps)
J
K
L
RNA granules (%)
n.s.
Anterograde
Retrograde
Stationary
Bi-directional
M
Average speed (µm/min)
n=98
n=103
N
Frequency distribution (%)
Speed of RNA granules (µm/min)

Figure S1

Figure S1. Related to Figure 1. UTP Analogue Incorporates into mRNA and rRNA, but Not DNA, *in vivo*

(A) Introduction of biotin-UTP into the CNS prior to RNA extraction and streptavidin pull down for Bioanalyzer analysis and RT-qPCR. (B) RNA gel image from the Bioanalyzer analysis. Str., Stringency; Lad, ladder; Con, control; Bio, biotin-UTP. (C) Electropherogram showing that the global RNA patterns are similar for the input of control (blue) and biotin-UTP (red) conditions. (D and E) Electropherograms displaying the enrichment of RNA in the biotin-UTP condition with both the low (D) and high (E) stringency pull down protocols. (F) Bar charts presenting the enrichment of total RNA ($F_{1,8}$=99.67, $p<0.0001$), 18S rRNA ($F_{1,8}$=4601, $p<0.0001$), 28S rRNA ($F_{1,8}$=1087, $p<0.0001$), SDHA mRNA ($F_{1,7}$=158.9, $p<0.0001$), β-actin mRNA ($F_{1,8}$=105.4, $p<0.0001$) and rps13 mRNA ($F_{1,8}$=125.1, $p<0.0001$) in the biotin-UTP condition over control condition with both the low and high stringency pull down protocols. (G) Introduction of biotin-UTP into the CNS prior to genomic and mitochondrial DNA extraction and streptavidin pull down for Bioanalyzer analysis. (H) DNA gel image from the Bioanalyzer analysis. (I) Electropherogram showing the global DNA patterns are similar for the input of control (blue) and biotin-UTP (red) conditions. (J and K) Electropherograms showing no obvious DNA peak was detected in either control or biotin-UTP condition with both the low (J) and high (K) stringency pull down protocols. (L) The proportions of RNA motion in 20 individual axons are displayed as bars ($F_{3,76}$=19.7, $p<0.0001$). The proportions of RNA motion pooled together from all RNA granules (n=319) are displayed as red dotted lines. (M) Average speed of anterograde and retrograde trafficking of RNA granules (U=4463, p=0.16, Mann Whitney test). (N) Frequency distribution of the speed of RNA granules trafficked in the anterograde and retrograde directions. Error bars represent SEM. *p<0.05, **p<0.01, ***p<0.001 (two-way ANOVA for (F)) (one-way ANOVA with Tukey multiple comparisons test for (L)).

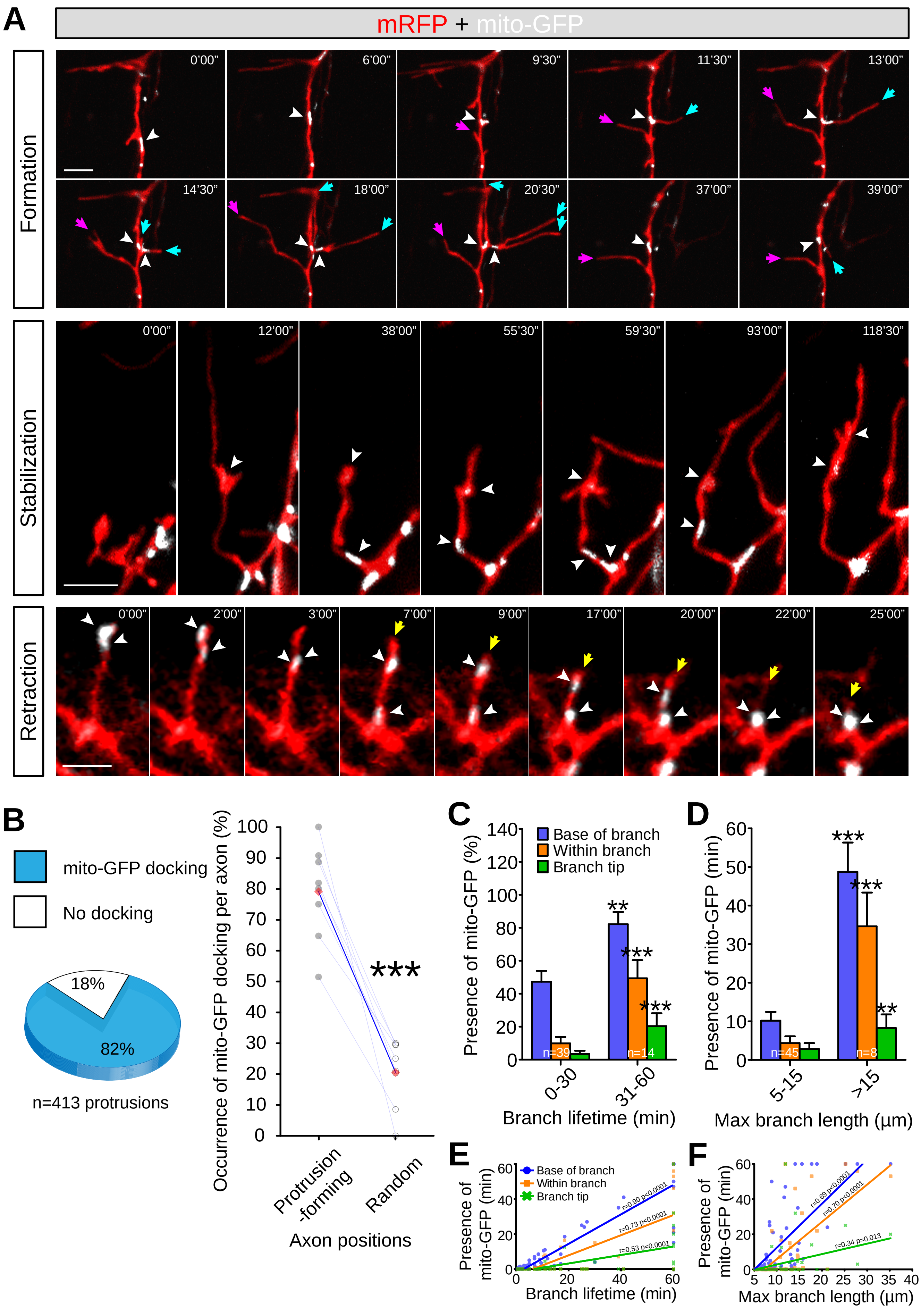
A
mRFP + mito-GFP
Formation
Stabilization
Retraction
B
mito-GFP docking
No docking
18%
82%
n=413 protrusions
Occurrence of mito-GFP docking per axon (%)
***
Protrusion -forming
Random
Axon positions
C
Base of branch
Within branch
Branch tip
Presence of mito-GFP (%)
n=39
n=14
0-30
31-60
Branch lifetime (min)
D
Presence of mito-GFP (min)
n=45
n=8
5-15
>15
Max branch length (µm)
E
Presence of mito-GFP (min)
Branch lifetime (min)
F
Presence of mito-GFP (min)
Max branch length (µm)

Figure S2

Figure S2. Related to Figure 1. Mitochondria Localization at Specific Axonal Sites Correlates with Branching *in vivo*

(A) Time-lapse images illustrating mitochondria labeled with mito-GPF (white arrowheads) docking in RGC axons during branching. (Upper panel) A branch was formed (magenta arrows) shortly after the departure of the mitochondrion. The accumulation of mitochondria at specific axonal sites also promoted the formation of branches (cyan arrows). (Middle panel) Mitochondria moved into branches and to branch tips during protrusion stabilization. (Bottom panel) Branch retraction (yellow arrow) occurred shortly after mitochondria had moved out of the branch. (B) (Left panel) Proportion of protrusions with mitochondria docking at the base ≥1min preceding protrusion formation. (Right panel) Occurrence of mitochondria docking in protrusion-forming or random positions in the same axons (t_7=8.3, p<0.0001, paired t test). Red diamonds represent the averages. (C) Time of mitochondria present at different branch positions (normalized to branch lifetime) were higher in branches with longer lifetime (base of branch: U=146, p=0.0088; within branch: U=116, p=0.0002; branch tip: U=145, p=0.0004). (D) Time of mitochondria present at different branch positions were higher in branches with longer maximal branch length (base of branch: U=26.5, p<0.0001; within branch: U=46, p<0.0001; branch tip: U=95.5, p=0.0044). Error bars represent SEM. **p<0.01, ***p<0.001 (Mann Whitney test for C-D)). (E) Correlation between time of mitochondria present at different branch positions and lifetime of branch (base of branch: r=0.90; within branch: r=0.73; branch tip: r=0.53, p<0.0001 for all groups). (F) Correlation between time of mitochondria present at different branch positions and maximal branch length (base of branch: r=0.69, p<0.0001; within branch: r=0.70, p<0.0001; branch tip: r=0.34, p=0.013, Pearson's correlation for (E and F)). Scale bars, 5μm.

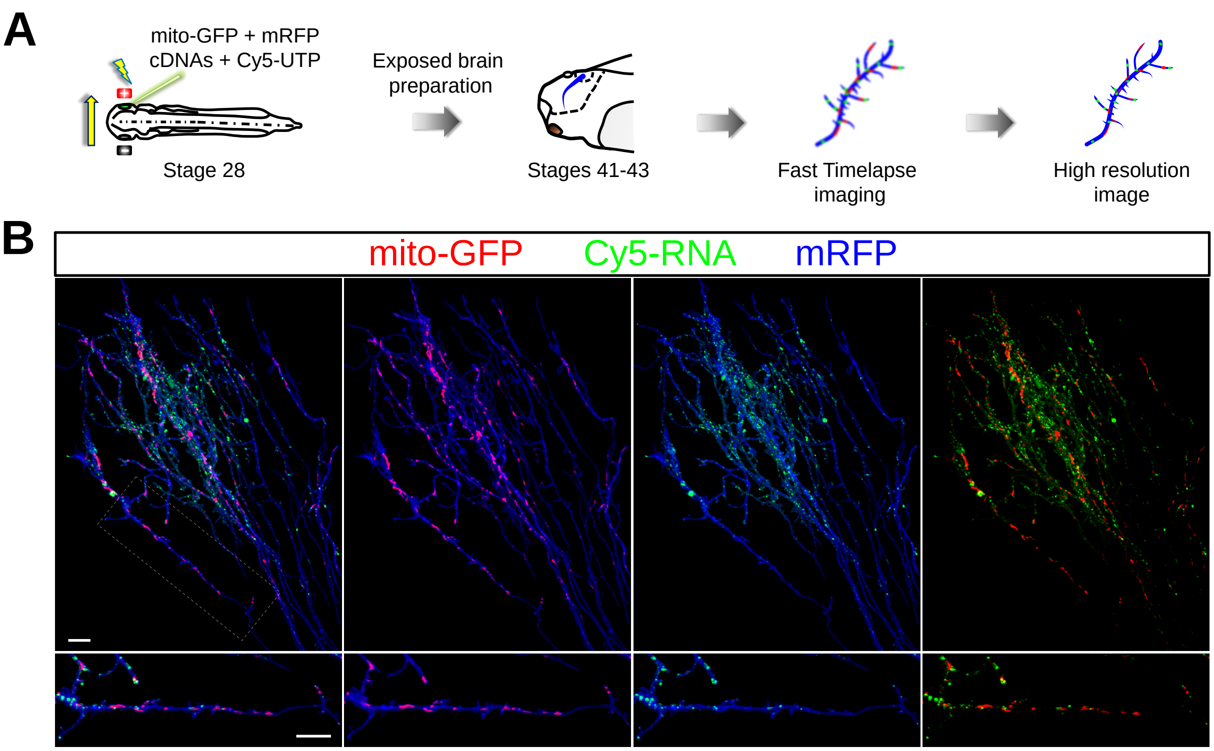

C

		Image scrambling		
Brain #	R(obs)	Iterations	R(rand) mean±sd	R(rand)>R(obs)
1	-0.016	1000	0.468±0.001	1000/1000
2	0.004	1000	0.504±0.001	1000/1000
3	0.113	1000	0.401±0.001	1000/1000
4	0.176	1000	0.368±0.001	1000/1000
5	0.048	1000	0.458±0.001	1000/1000
6	-0.094	1000	0.478±0.001	1000/1000
7	-0.039	1000	0.443±0.001	1000/1000
8	0.058	1000	0.367±0.001	1000/1000
9	-0.033	1000	0.456±0.001	1000/1000
10	0.051	1000	0.440±0.001	1000/1000
11	0.136	1000	0.420±0.001	1000/1000

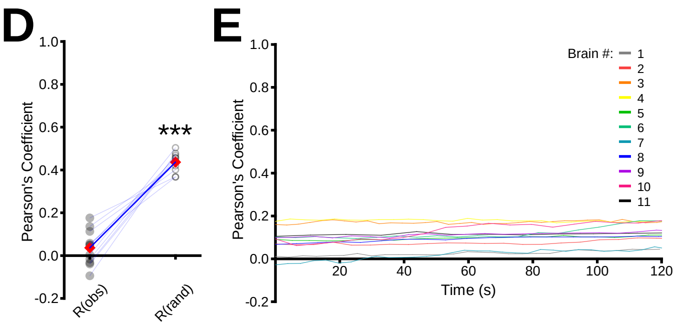

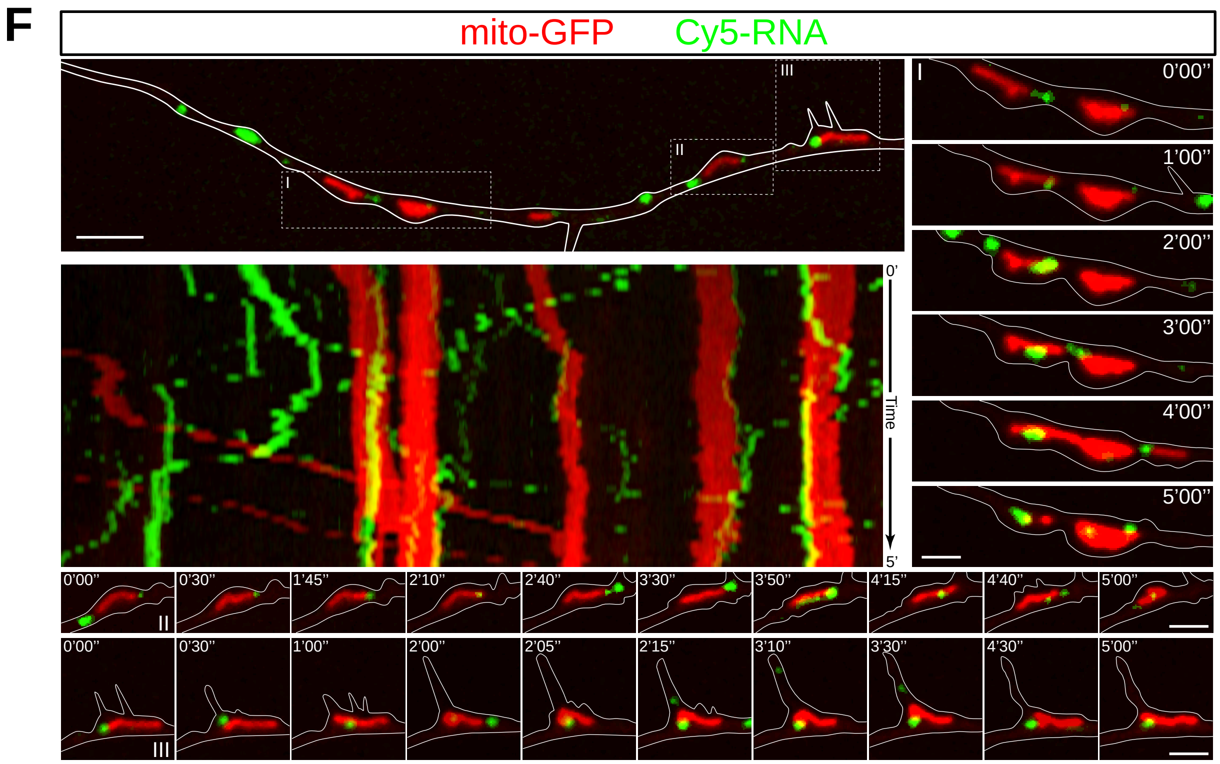

Figure S3

Figure S3. Related to Figure 1. Cy5-RNA and Mitochondria Are Distinct Structures

(A) Schematic of introducing Cy5-UTP, mito-GFP cDNA and mRFP cDNA into the RGCs with targeted eye electroporation. Fast *in vivo* time-lapse imaging of RNA granules, mitochondria and cell morphology was carried out for 2 minutes. This was followed by capturing a high resolution image stack of the axon arbors *in vivo*. (B) A high resolution image stack showing the localization of RNA granules and mitochondria in axon arbors. The RNA granules and mitochondria seldom showed colocalization but are often seen to juxtapose each other. (C) A table showing the Pearson's correlation coefficient (R) for Cy5-RNA and mito-GFP in the high resolution image stacks of 11 brains. Each image stack was individually scrambled to create 1000 random images. In all cases, the R(rand) yielded from the 1000 scrambled images were higher than the observed R(obs), robustly indicating that none of the samples showed a higher colocalization of the Cy5-RNA and mito-GFP than expected from chance. (D) The averages of R(rand) yielded from 1000 scrambled images were significantly higher than their R(obs) from the original high resolution image stacks (t_{10}=11.25, $p<0.0001$, paired t test), supporting that the Cy5-RNA and mitochondria are distinct structures. (E) A line graph illustrating the Costes Pearson's correlation coefficient remains low and similar across time from the time-lapse images (average image intervals= 5.8±0.6s per z-stack), supporting the observed low R(obs) in the high resolution image stack were not "artifacts" from a snapshot. (F) Time-lapse images showing the localizing of RNA granules and mitochondria in an RGC axon of eye explant. The kymograph and time series I, II and III show that Cy5-RNA and mitochondria are distinct structures and often juxtapose each other, reflecting potential interaction may exist. Error bars represent SEM. ***$p<0.001$. Scale bars, 10μm for (B), 5μm for (F) overview image and 2μm for (F) time series I, II and III.

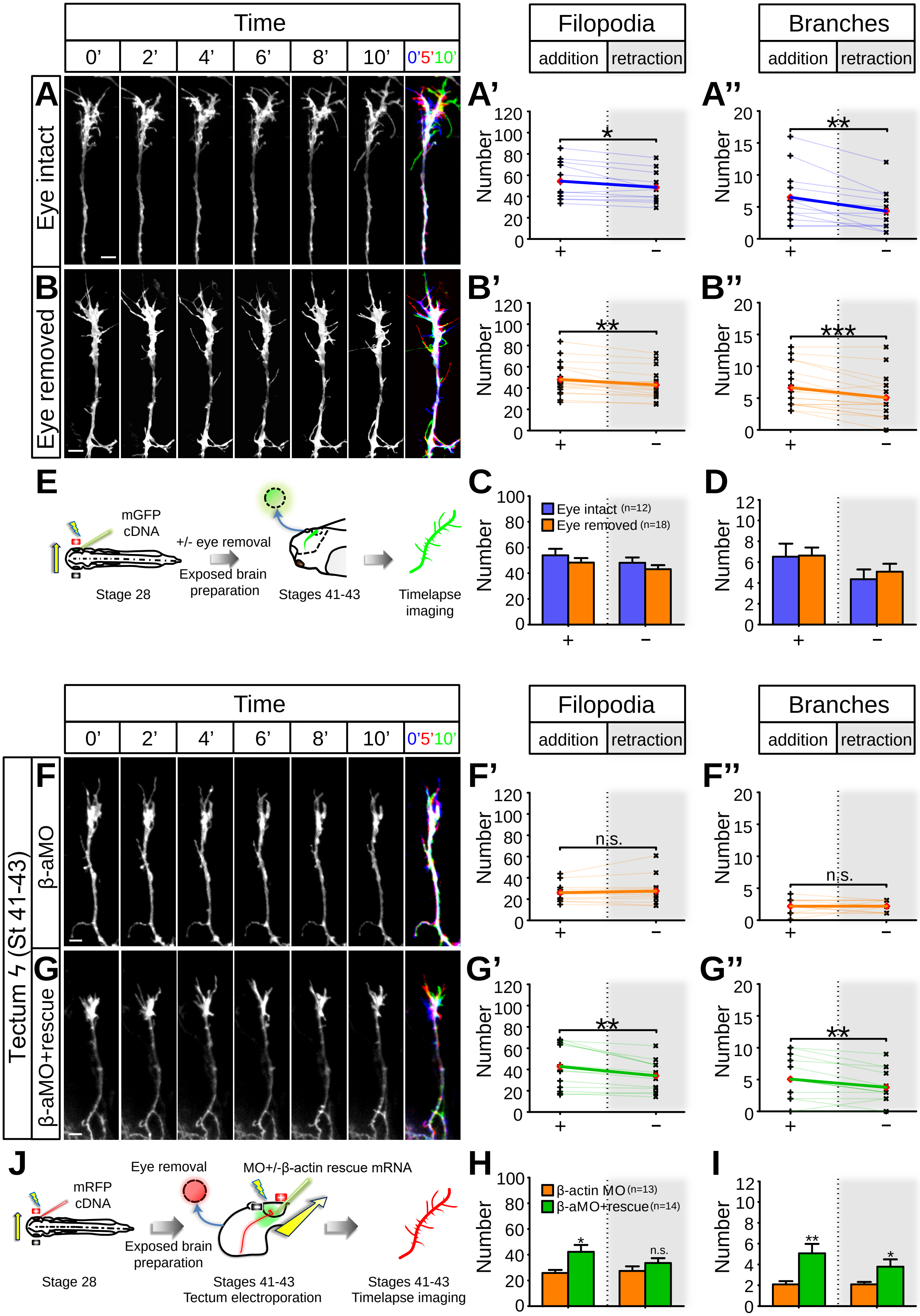

Time
0’
2’
4’
6’
8’
10’
0’5’10’
Filopodia
addition
retraction
Branches
addition
retraction
A
Eye intact
B
Eye removed
A’
A’’
B’
B’’
Number
E
mGFP
cDNA
+/- eye removal
Exposed brain
preparation
Stage 28
Stages 41-43
Timelapse
imaging
C
D
Eye intact (n=12)
Eye removed (n=18)
F
β-aMO
G
β-aMO+rescue
Tectum ϟ (St 41-43)
F’
F’’
G’
G’’
n.s.
J
mRFP
cDNA
Eye removal
MO+/-β-actin rescue mRNA
Exposed brain
preparation
Stage 28
Stages 41-43
Tectum electroporation
Stages 41-43
Timelapse imaging
H
I
β-actin MO (n=13)
β-aMO+rescue (n=14)

Figure S4

Figure S4. Related to Figure 2 and 5. Axonal Branching Dynamics Remains Normal after Eye Removal and Branching Dynamics Defects Resulted from Local Inhibition of β-actin Translation are Rescued by Co-delivery of β-actin mRNA

(A and B) Time-lapse images of axonal branching in embryos with eye (soma of RGC axons) intact (A) and removed (B). (A' and A") Higher number of filopodia and branches were added than removed in embryos with eye intact (filopodia: t_{11}=3.1, p=0.01; branches: t_{11}=3.8, p=0.003). (B' and B") Higher number of filopodia and branches were added than removed in embryos with eye removed filopodia: t_{17}=3.4, p=0.003; branches: t_{17}=4.6, p=0.0002). (C and D) The addition and removal of filopodia (C; addition: t_{28}=0.95, p=0.35; removal: t_{28}=0.97, p=0.34) and branches (D; addition: t_{28}=0.08, p=0.94; removal: t_{28}=0.60, p=0.56) were unaffected after the removal of the eye. (E) Schematic of live imaging experiment on branching dynamics of RGC axons with eye intact/removed. (F and G) Time-lapse images of axon branching in the tectum after local delivery of β-actin MO (+/- β-actin rescue mRNA [0.5µM]) at stages 41-43. (F' and F") No significant differences were observed in the number of filopodia and branches that were added and removed after local delivery of β-actin MO (β-aMO) (addition: t_{12}=1.1, p=0.30; removal: t_{12}=0, p=1). (G' and G") The loss of the addition:removal biases of filopodia and branches after local delivery of β-aMO were rescued with local co-delivery of β-aMO resistant rescue mRNA. (filopodia: t_{13}=3.9, p=0.002; branches: t_{13}=3.3, p=0.005). (H) The addition of filopodia was rescued with β-actin rescue mRNA (t_{25}=2.7, p=0.01). Despite a trend of rescue was seen for the removal of filopodia, it was not statically significant (removal: t_{25}=1.2, p=0.24). (I) The addition and removal of filopodia and branches were rescued with β-actin rescue mRNA (D; addition: t_{25}=3.0, p=0.006; removal: t_{25}=2.2, p=0.04). (J) Schematic showing the local delivery of β-actin MO (+/- β-actin rescue mRNA) into RGC axons by tectum electroporation and imaged immediately thereafter. Electroporated eye was removed to eliminate somatic contribution of cytoplasmic material. Error bars represent SEM. *p<0.01, **p<0.01, ***p<0.001 (paired t test for (A, B, F and G) and unpaired t test for (C, D, H and I). Scale bars, 5µm. Red diamonds represent the averages (A, B, F and G).

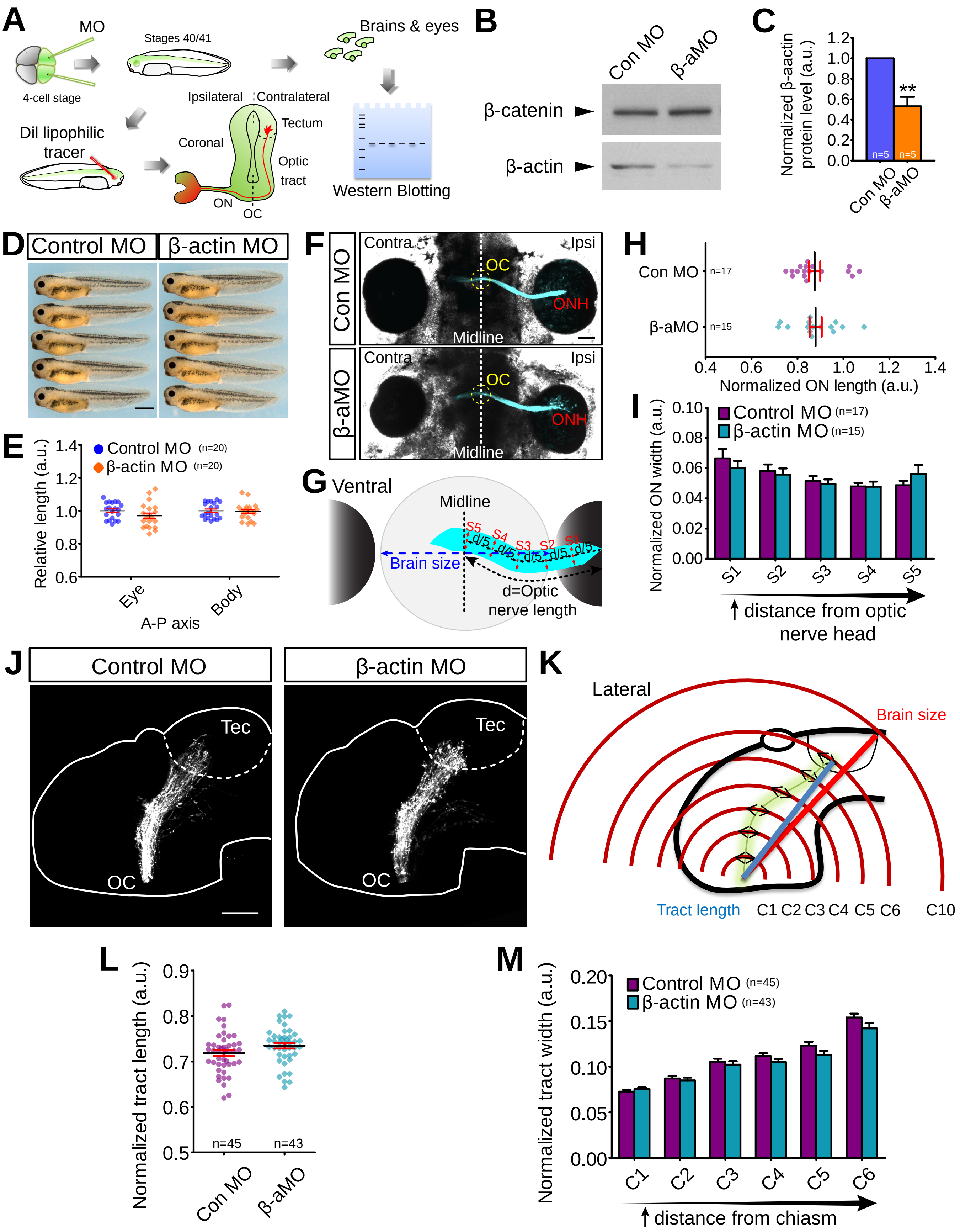
A
MO
4-cell stage
Stages 40/41
Brains & eyes
Dil lipophilic tracer
Ipsilateral
Contralateral
Tectum
Coronal
Optic tract
ON
OC
Western Blotting
B
Con MO
β-aMO
β-catenin
β-actin
C
Normalized β-aactin protein level (a.u.)
n=5
Con MO
β-aMO
D
Control MO
β-actin MO
E
Relative length (a.u.)
Control MO (n=20)
β-actin MO (n=20)
Eye
Body
A-P axis
F
Con MO
β-aMO
Contra
Ipsi
OC
ONH
Midline
G
Ventral
Midline
Brain size
d=Optic nerve length
H
Con MO
β-aMO
n=17
n=15
Normalized ON length (a.u.)
I
Normalized ON width (a.u.)
Control MO (n=17)
β-actin MO (n=15)
distance from optic nerve head
J
Control MO
β-actin MO
Tec
OC
K
Lateral
Brain size
Tract length
C1 C2 C3 C4 C5 C6 C10
L
Normalized tract length (a.u.)
n=45
n=43
Con MO
β-aMO
M
Normalized tract width (a.u.)
Control MO (n=45)
β-actin MO (n=43)
distance from chiasm

Figure S5

Figure S5. Related to Figure 4. Embryo Remains Grossly Normal after β-actin Knockdown

(A) Schematic of MO injection into dorsal animal blastomeres at 4-cell stage. Injected embryos at Stage 41 were then used for anterograde tracing of RGC axons with lipophilic dye or protein harvesting of brain and eyes for western blot analysis. (B) Western blot showing β-actin was markedly reduced in the β-actin knockdown condition compared to control. β-catenin served as a loading control. (C) Normalized ratiometric measure of β-actin knockdown efficiency from western blot analyses (t_4=5.0, p=0.0073, paired t test). (D) Brightfield images showing that the morphology of β-actin morphants and their eyes being grossly normal. (E) Quantification of the length of eye (t_{38}=1.5, p=0.14) and body (t_{38}=0.3, p=0.76) in the anterior-posterior axis displaying no significant differences. (F) Images from ventral perspective of the brain showing the RGC axon bundles projecting from the optic nerve head (ONH) to the optic chiasm (OC) were indiscernible between control and β-actin morphants. (G) Schematic illustrating the quantification of the length and width of optic nerve. The optic nerve length (d) is defined by the length of the optic nerve (ON) from the ONH to the midline. The length was then divided into 5 sections (S1-S5), which are points where the width of the optic nerve was measured perpendicularly to the length axis. Both the length and width of the ON were then normalized to the brain size, which is defined by the straight line from the point of the brain adjacent to the center of the eye to the midline of the brain. (H) Scatter graph showing that the length of the ON from the ONH to the midline did not differ between the control and β-actin morphants (t_{30}=0.1, p=0.91). (I) The ON width didnot differ between the control and β-actin morphants across the length of the ON from the ONH to the midline (S1: t_{30}=0.8, p=0.43; S2: t_{30}=0.4, p=0.70; S3: t_{30}=0.5, p=0.64; S4: t_{30}=0.04, p=0.97; S5: t_{30}=1.2, p=0.24). (J) Images from the lateral perspective of the contralateral hemisphere of brain showing that retinotectal projection from the OC to the optic tectum were indiscernible between control and β-actin morphants. (K) Schematic illustrating the quantification of the length and width of optic tract. The optic tract length is defined by a straight line from the OC to the distal axon terminals of the optic tract. Ten equally spaced concentric circles (C1-C10) were overlaid on the tract images with the center of the circles overlying the OC and C10 overlaying the dorsal-posterior tectal boundary. Both the length and width of tract were then normalized to the brain size, which is defined by the straight line from the OC to the dorsal-posterior tectal boundary. (L) Scatter graph showing that the length of the optic tract did not differ between the control and β-actin morphants (t_{86}=1.7, p=0.086). (M) The optic tract width did not differ between the control and β-actin morphants across the length of the optic tract (C1: t_{86}=1.1, p=0.27; C2: t_{86}=0.5, p=0.62; C3: $t8_6$=0.6, p=0.56; C4: t_{86}=1.4, p=0.17; C5: t_{86}=1.7, p=0.10; C6: t_{86}=1.7, p=0.092). Error bars represent SEM. **p<0.01 (unpaired t test for (E, H, I, L and M)). Scale bars: 1mm for (D) and 100μm for (F and J).

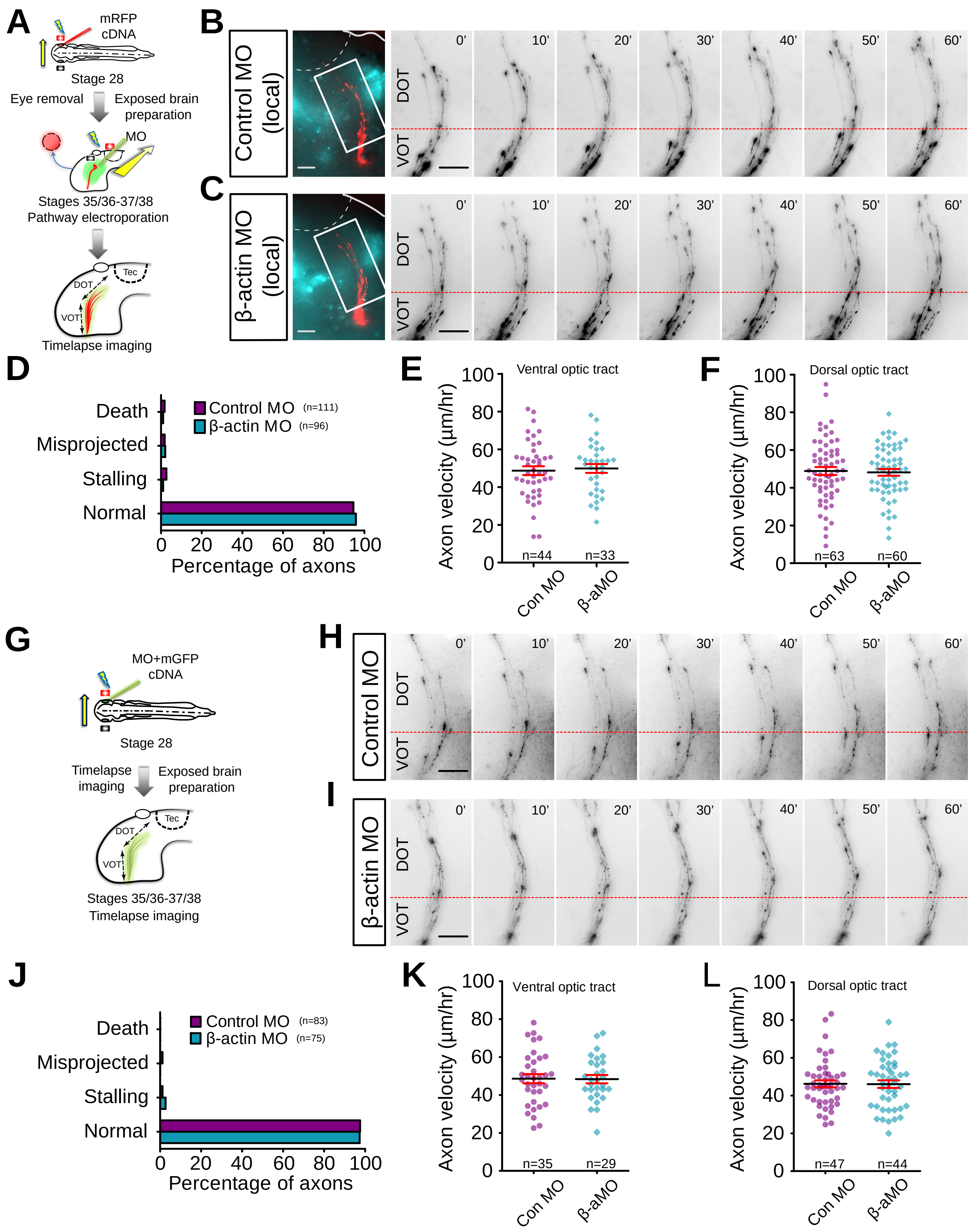
A
mRFP
cDNA
Stage 28
Eye removal
Exposed brain
preparation
MO
Stages 35/36-37/38
Pathway electroporation
Tec
DOT
VOT
Timelapse imaging
B
Control MO
(local)
C
β-actin MO
(local)
0'
10'
20'
30'
40'
50'
60'
DOT
VOT
D
Death
Misprojected
Stalling
Normal
Control MO (n=111)
β-actin MO (n=96)
0 20 40 60 80 100
Percentage of axons
E
Ventral optic tract
Axon velocity (µm/hr)
n=44
n=33
Con MO
β-aMO
F
Dorsal optic tract
n=63
n=60
G
MO+mGFP
cDNA
Stage 28
Timelapse
imaging
Exposed brain
preparation
Stages 35/36-37/38
Timelapse imaging
H
Control MO
I
β-actin MO
J
Control MO (n=83)
β-actin MO (n=75)
K
Ventral optic tract
n=35
n=29
L
Dorsal optic tract
n=47
n=44

Figure S6

Figure S6. Related to Figure 5. Local Inhibition of β-actin Translation Does Not Grossly Affect Axon Navigation in the Optic Tract

(A) Schematic showing the local delivery of MO into RGC axons by pathway electroporation. The eye electroporated with mRFP plasmids was removed to eliminate somatic contribution of cytoplasmic material. The embryos were imaged immediately after local delivery of MO to ensure any observed effect would be a result of acute blockage of local β-actin translation. (B and C) Time-lapse images of RGC axons navigating through the ventral optic tract (VOT) and dorsal optic tract (DOT) after local Con MO (B) or β-actin MO (C) delivery. MO is shown in cyan and axons are shown in red on the left. (D) Axon behaviors during navigation were unaffected in axons with local β-actin MO delivery (death: p=1; misprojected: p=1; stalling: p=0.63; normal: p=0.75). (E and F) Axon navigation velocities in both the VOT (E) and DOT (F) were unaffected local β-actin MO delivery. (VOT: t_{75}=0.3, p=0.73; DOT: t_{121}=0.3, p=0.80). (G) Schematic showing eye electroporation and live imaging of axon navigation. (H and I) Time-lapse images of axon navigation through the ventral optic tract (VOT) and dorsal optic tract (DOT) of Con MO- and β-actin MO-positive RGC axons. (J) Axon behaviors during navigation were unaffected in axons from the β-actin MO condition. (misprojected: p=1 stalling: p=0.6; normal: p=1)(K and L) Axon navigation velocities in both the VOT (K) and DOT (L) were unaffected after β-actin knockdown in RGCs (VOT: t_{62}=0.07, p=0.94; DOT: t_{89}=0.09, p=0.95). Error bars represent SEM. (Fisher's exact test for (D and J)). (unpaired t test for (E, F, K and L)). Scale bars, 50μm for (B, C, H and I).

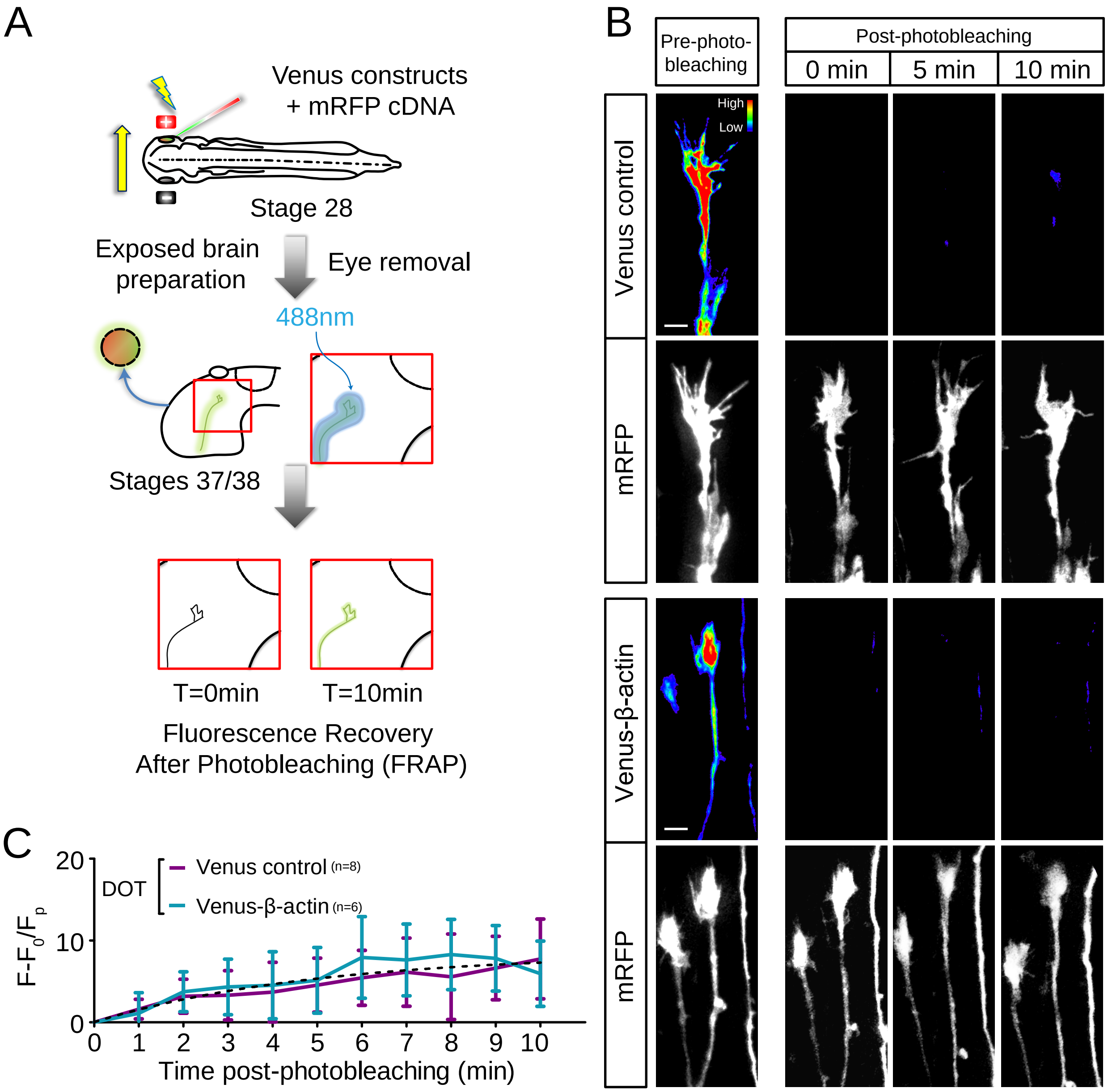

A
Venus constructs
+ mRFP cDNA
Stage 28
Exposed brain
preparation
Eye removal
488nm
Stages 37/38
T=0min
T=10min
Fluorescence Recovery
After Photobleaching (FRAP)
B
Pre-photo-
bleaching
Post-photobleaching
0 min
5 min
10 min
High
Low
Venus control
mRFP
Venus-β-actin
mRFP
C
DOT
Venus control (n=8)
Venus-β-actin (n=6)
F-F0/Fp
20
10
0
0 1 2 3 4 5 6 7 8 9 10
Time post-photobleaching (min)

Figure S7

Figure S7. Related to Figure 6. Local β-actin Synthesis Is Not Detected in Growth Cones Navigating the Optic Tract

(A) Experimental design of fluorescence recovery after photobleaching experiment of Venus-constructs in the optic tract *in vivo*. Electroporated eye was removed to eliminate somatic contribution of cytoplasmic material. OT, optic tract. (B) Fluorescence heatmaps illustrating that limited recoveries were detected with both the Venus control and Venus-β-actin constructs. (C) Line graph showing the recovery of Venus signals after photobleaching over the course of 10 minutes. Dotted line represents least-squares fit to a single-exponential function; note that a single model is highly adequate for fitting both conditions (Venus control vs. Venus-β-actin: $F_{3,148}$=0.15, p=0.93, Extra sum-of-squares F test). Error bars represent SEM. Scale bars, 5μm.
